# *Chlamydomonas reinhardtii* LFO1 Is an IsdG Family Heme Oxygenase

**DOI:** 10.1128/mSphere.00176-17

**Published:** 2017-08-16

**Authors:** Lisa J. Lojek, Allison J. Farrand, Jennifer H. Wisecaver, Crysten E. Blaby-Haas, Brian W. Michel, Sabeeha S. Merchant, Antonis Rokas, Eric P. Skaar

**Affiliations:** a Department of Pathology, Microbiology, & Immunology, Vanderbilt University Medical Center, Nashville, Tennessee, USA; b Graduate Program in Microbiology & Immunology, Vanderbilt University, Nashville, Tennessee, USA; c Department of Biological Sciences, Vanderbilt University, Nashville, Tennessee, USA; d Department of Chemistry and Biochemistry, University of California, Los Angeles, Los Angeles, California, USA; e Department of Chemistry and Biochemistry, University of Denver, Denver, Colorado, USA; University of Iowa

**Keywords:** *Chlamydomonas*, bilin, heme, heme degradatrion, iron, monooxygenases

## Abstract

Heme is essential for respiration across all domains of life. However, heme accumulation can lead to toxicity if cells are unable to either degrade or export heme or its toxic by-products. Under aerobic conditions, heme degradation is performed by heme oxygenases, enzymes which utilize oxygen to cleave the tetrapyrrole ring of heme. The HO-1 family of heme oxygenases has been identified in both bacterial and eukaryotic cells, whereas the IsdG family has thus far been described only in bacteria. We identified a hypothetical protein in the eukaryotic green alga *Chlamydomonas reinhardtii*, which encodes a protein containing an antibiotic biosynthesis monooxygenase (ABM) domain consistent with those associated with IsdG family members. This protein, which we have named LFO1, degrades heme, contains similarities in predicted secondary structures to IsdG family members, and retains the functionally conserved catalytic residues found in all IsdG family heme oxygenases. These data establish LFO1 as an IsdG family member and extend our knowledge of the distribution of IsdG family members beyond bacteria. To gain further insight into the distribution of the IsdG family, we used the LFO1 sequence to identify 866 IsdG family members, including representatives from all domains of life. These results indicate that the distribution of IsdG family heme oxygenases is more expansive than previously appreciated, underscoring the broad relevance of this enzyme family.

**IMPORTANCE** This work establishes a protein in the freshwater alga *Chlamydomonas reinhardtii* as an IsdG family heme oxygenase. This protein, LFO1, exhibits predicted secondary structure and catalytic residues conserved in IsdG family members, in addition to a chloroplast localization sequence. Additionally, the catabolite that results from the degradation of heme by LFO1 is distinct from that of other heme degradation products. Using LFO1 as a seed, we performed phylogenetic analysis, revealing that the IsdG family is conserved in all domains of life. Additionally, *C. reinhardtii* contains two previously identified HO-1 family heme oxygenases, making *C. reinhardtii* the first organism shown to contain two families of heme oxygenases. These data indicate that *C. reinhardtii* may have unique mechanisms for regulating iron homeostasis within the chloroplast.

## Abstract (precis)

This work establishes a protein in the freshwater alga *Chlamydomonas reinhardtii* as an IsdG family heme oxygenase. This protein, LFO1, exhibits predicted secondary structure and catalytic residues conserved in IsdG family members, in addition to a chloroplast localization sequence. Additionally, the catabolite that results from the degradation of heme by LFO1 is distinct from that of other heme degradation products. Using LFO1 as a seed, we performed phylogenetic analysis, revealing that the IsdG family is conserved in all domains of life. Additionally, *C. reinhardtii* contains two previously identified HO-1 family heme oxygenases, making *C. reinhardtii* the first organism shown to contain two families of heme oxygenases. These data indicate that *C. reinhardtii* may have unique mechanisms for regulating iron homeostasis within the chloroplast.

## INTRODUCTION

Heme is essential for a variety of cellular processes, including serving as an enzymatic cofactor for catalase and acting as an electron acceptor in the electron transport chain (1–5). Additionally, many cells express enzymes to degrade heme. These enzymes, known as heme oxygenases, utilize oxygen to cleave the porphyrin ring of heme to release free iron and secondary catabolites. While many organisms encode heme oxygenases, there is diversity in enzyme structure and catabolite production that may reflect a diversity of function. The first identified family of heme oxygenases is the HO-1 family, which is found in both eukaryotic and bacterial cells (5–10). The members of the HO-1 family degrade heme to biliverdin, carbon monoxide, and free iron (5). The members of a second family of heme oxygenases, known as the IsdG family, degrade heme to staphylobilin, formaldehyde, and free iron (11, 12). Recent work identified MhuD, a protein with significant secondary structure similarities and conserved catalytic residues with respect to IsdG family members (13). However, MhuD degrades heme to distinct products, namely, mycobilin and free iron (14). Finally, an oxygen-independent heme-degrading enzyme, ChuW, was discovered in *Escherichia coli*. ChuW uses radical catalysis to degrade heme to the small molecule anaerobilin and free iron (15).

The IsdG family has been characterized in only a limited number of bacterial species from diverse lineages (16). These include the Gram-positive *Firmicutes* (16–20) and *Actinobacteria* (14) and the Gram-negative *Alphaproteobacteria* (21). All of the enzymes within this family have conserved secondary structures and catalytic residues and contain an antibiotic biosynthesis monooxygenase (ABM) domain (22, 23). Additionally, many of the IsdG family heme oxygenases are also selectively expressed under low-iron conditions. Among the seven experimentally characterized proteins within the IsdG family, the amino acid sequence identities range from 20% to 60% (16). Phylogenetic analysis has revealed 22 unique IsdG family heme oxygenases, all within bacterial species (16). However, due to the low sequence identity between the IsdG family members and the diversity of the bacterial species in which they were identified, we hypothesized that the IsdG family extends beyond this small number of species.

Previous work in *Chlamydomonas reinhardtii* identified a gene, *C. reinhardtii* 07.g312300 (Cre07.g312300), that is more highly expressed under iron-limited conditions than under iron-replete conditions (24). Analysis of the putative protein encoded by this gene identified an ABM domain and specific secondary structure motifs. Alignment of these structural characteristics with heme oxygenases led us to hypothesize that this putative protein, which we have named LFO1 (for low-Fe-responsive oxygenase 1), belongs to the IsdG family of heme oxygenases. Here we describe the characterization of LFO1 as a functional heme oxygenase that requires catalytic residues conserved among IsdG family members for activity. Degradation of heme by LFO1 leads to a product with chromatographic properties that are unique in comparison to those of other identified heme catabolites. Using the LFO1 protein sequence, we queried the entirety of known protein sequences for additional IsdG family heme oxygenases. Through this analysis, we identified 866 unique proteins from across the tree of life, significantly expanding knowledge of the taxonomic distribution of the members of the IsdG family.

## RESULTS

### LFO1 exhibits structural similarity to IsdG family heme oxygenases.

**FIG 1:**
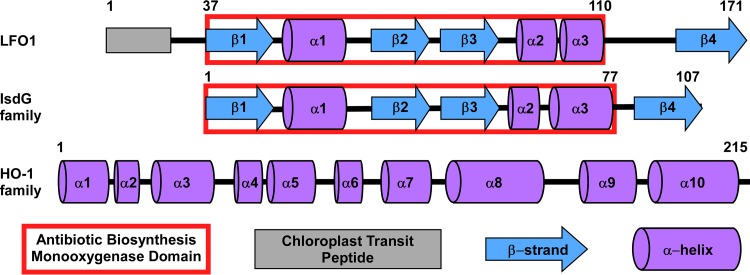
The LFO1 secondary structure is more similar to the secondary structures of members of the IsdG family of heme oxygenases than to those of members of the HO-1 family. The predicted structure of LFO1 is shown in comparison to that of a prototypical IsdG family member, IsdG from *S. aureus*, and in comparison to that of a HO-1 family heme oxygenase, HmuO from *C. diphtheriae* (32). The blue arrows represent β-strands, the purple cylinders represent α-helices, and the gray rectangle represents the chloroplast transit peptide in LFO1. The red outline represents the predicted antibiotic biosynthesis monooxygenase (ABM) domain.

*C. reinhardtii* Cre07.g312300 was identified in an RNA sequencing experiment as a transcript that is more highly expressed under iron-limited conditions than under iron-replete conditions (25). Cre07.g312300 is a nuclear gene that encodes a hypothetical protein of approximately 18 kDa (26). Pfam analysis of Cre07.g312300 protein domains identified a putative antibiotic biosynthesis monooxygenase (ABM) domain (27, 28) (Fig. 1, red box). Due to its similarity to genes encoding monooxygenases, we named the gene *Lfo1* (for low-Fe-responsive oxgenase 1). Secondary structure predictions indicated a secondary structure pattern (Fig. 1) that consists of a β-, α-, β-, β-, α-, α-, β-motif (22). In addition, there is a 45-amino-acid predicted unstructured region between α-helix 3 and β-strand 4 (see Fig. 3). The sequence of this region predominantly consists of serine (S), glycine (G), and histidine (H) residues. The N terminus of LFO1 consists of a predicted chloroplast transit peptide, with a PredAlgo *C* score of 2.32 (Fig. 1) (29), where a *C* score above 0.41 predicts that the protein localizes to the chloroplast.

The predicted secondary structure of LFO1 is very similar to that of the IsdG family of heme oxygenases (Fig. 1) (22). IsdG family heme oxygenases generally have low sequence identity; however, they have highly conserved secondary structures (22, 23), and all of the IsdG family members contain an ABM domain (Fig. 1). The secondary structure of IsdG family heme oxygenases is quite different from the secondary structure seen in HO-1 family heme oxygenases (Fig. 1), which is composed of 10 α-helices (10, 30–32). These characteristics led us to hypothesize that LFO1 is an IsdG family heme oxygenase.

### LFO1 binds and degrades heme.

**FIG 2:**
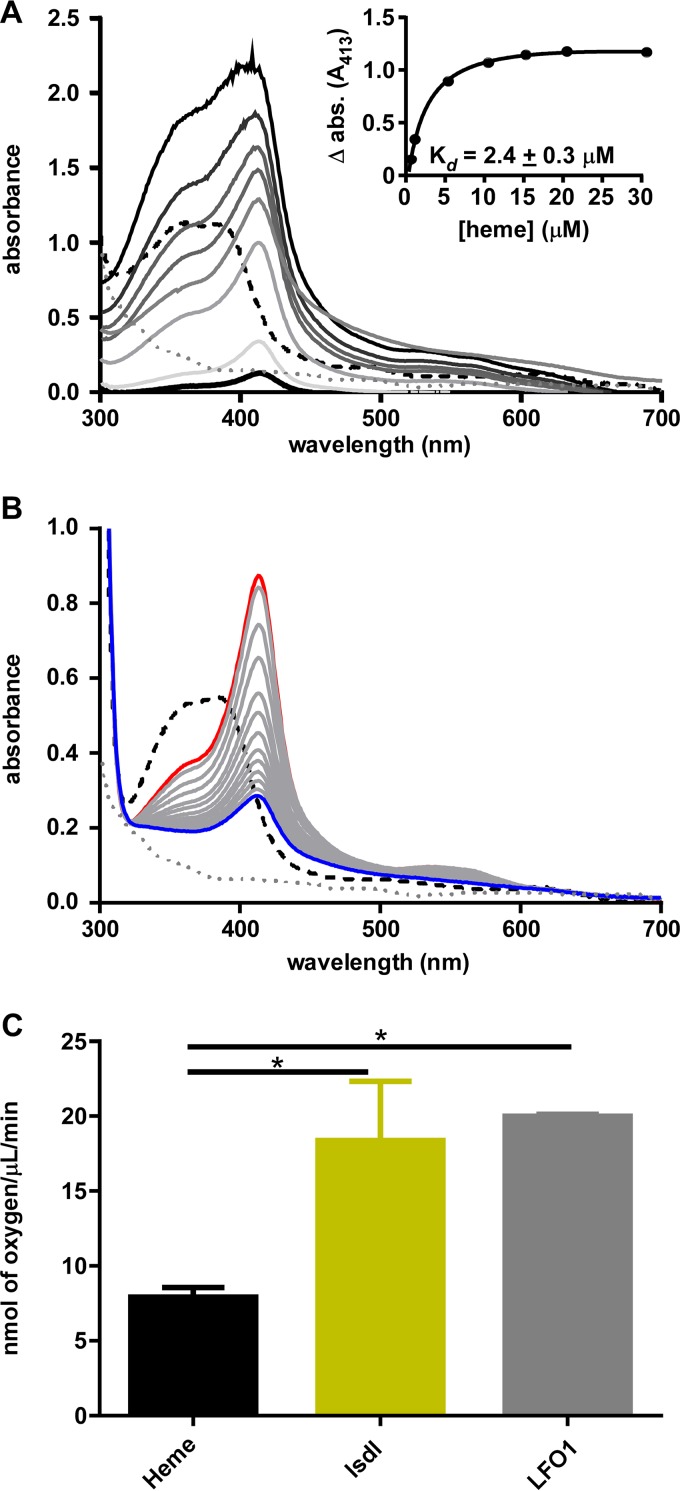
LFO1 binds and degrades heme *in vitro*. (A) Absorption spectra of heme binding to LFO1. Increasing concentrations (0.5 to 30 μM) of heme were added to 10 μM protein. The spectrum corresponding to 10 μM heme is shown with a dashed black line, and that corresponding to LFO1 alone is shown with a dotted gray line, whereas the protein incubated with heme is shown with solid lines, with increasing heme concentrations leading to an increase in peak height. The inset displays the change in absorbance (Δ abs.) at 413 nm for LFO1 bound to heme minus heme alone corresponding to heme concentrations of 1 to 30 μM. (B) Heme degradation reactions with LFO1. LFO1 (10 μM) was incubated with equimolar heme, and then ascorbic acid was added as the reducing agent. The spectrum was monitored from 0 min (red line) to 60 min (blue line), with data obtained every 5 min (gray lines). (C) The rates of oxygen consumption for heme alone, Isdl, and LFO1 were monitored via oxygraph. The rates of oxygen utilization for Isdl and LFO1 were significantly different from those seen with heme alone. *, *P* < 0.05.

Heme has a distinct visible spectrum that peaks at ~390 nm (Fig. 2A, black dashed line). Upon complexation with heme binding proteins, this peak shifts to what is known as a Soret peak, with an absorption maximum of around 400 to 450 nm (33, 34). Heme oxygenase members of both the IsdG and HO-1 families typically exhibit a Soret peak of 405 to 413 nm (35, 36). Incubating heme with LFO1 leads to a shift in the peak to 413 nm (Fig. 2A), consistent with LFO1 binding heme. The ratio of binding of LFO1 to heme is 1:1, based on the inflection point having been at 10 μM heme when increasing concentrations of heme were added to LFO1 (Fig. 2A, inset). The binding affinity of LFO1 to heme was calculated at a dissociation constant (*K*_*d*_) value of 2.4 ± 0.3 μM (Fig. 2A, inset). The binding affinity of LFO1 is in the range of binding affinities calculated for various heme oxygenases, which range from 0.84 + 0.2 μM (37) to 5.0 + 1.5 μM (17). These data demonstrate that LFO1 binds heme with characteristics consistent with those of other IsdG family heme oxygenases.

**FIG 3:**
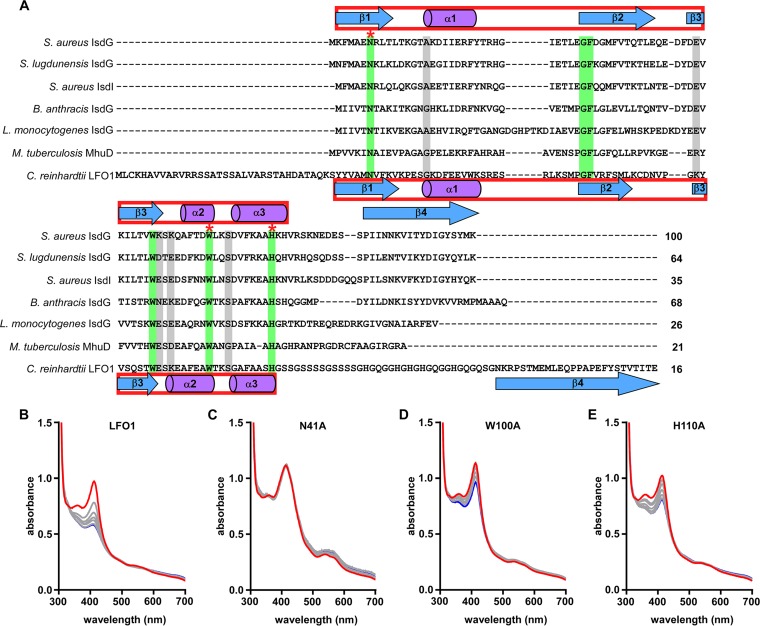
IsdG family catalytic residues are conserved in LFO1 and are required for heme degradation. (A) Alignment of the full-length sequences of six functionally characterized IsdG family heme oxygenases with LFO1. Identical residues are highlighted in green, and similar residues are highlighted in gray. The IsdG family catalytic residues are indicated with stars. Solved secondary structures for *S. aureus* IsdG are displayed above the alignment, and the predicted secondary structure for LFO1 is displayed below the alignment. Percent identity of each sequence compared to that of *S. aureus* IsdG is shown at the end of the sequence. *S. aureus*, *Staphylococcus aureus*; *S. lugdunensis*, *Staphylococcus lugdunensis*; *B. anthracis*, *Bacillus anthracis*; *L. monocytogenes*, *Listeria monocytogenes*; *M. tuberculosis*, *Mycobacterium tuberculosis*; *C. reinhardtii*, *Chlamydomonas reinhardtii*. (B to E) Heme degradation reactions, in the presence of catalase, were performed with wild-type LFO1 (B) or with LFO1 with point mutations in each of the conserved catalytic residues N41A (C), W100A (D), and H110A (E). The spectrum was monitored from 0 min (red line) to 60 min (blue line), with data obtained every 5 min (gray lines).

To determine whether LFO1 degrades heme, LFO1 was incubated with heme and ascorbate and the visible spectra were collected every 10 min for 1 h. Under these conditions, the LFO1-bound heme peak decreased over time, demonstrating that LFO1 degrades heme (Fig. 2B). Using the same reaction conditions, we tested the ability of LFO1 to degrade other metallo-protoporphyrins and found that LFO1 can degrade only heme (see Fig. S2 in the supplemental material). This finding is consistent with data from other heme oxygenases, since iron is required to facilitate the cleavage of the porphyrin ring (38, 39). The heme degradation reaction was also performed in the presence of catalase, which inhibits heme autoxidation. Once again, the 413-nm peak decreased with time (Fig. 3B), indicating that the degradation of heme is due to the catalytic activity of LFO1. These data establish LFO1 as a heme-degrading enzyme.

10.1128/mSphere.00176-17.1 FIG S1  Purification of His-LFO1 from *E. coli*. (A) Coomassie-stained gel of recombinantly expressed 6His-LFO1. (B) Western blot for 6His tag. For both gels, lane 1 represents the cellular lysate, lane 2 represents the column flowthrough, lanes 3 to 11 represent the stepwise decreasing concentrations of urea (from 8 M to 0 M), lane 12 represents a 100 mM imidazole wash, and lane 13 represents a 400 mM imidazole elution. Download FIG S1, TIF file, 0.01 MB. Copyright © 2017 Lojek et al. 2017 Lojek et al. This content is distributed under the terms of the Creative Commons Attribution 4.0 International license.

10.1128/mSphere.00176-17.2 FIG S2  His-LFO1 is unable to degrade noniron protoporphyrin. Data represent results of heme degradation reactions performed with LFO1. A 10 μM concentration of protein was incubated with equal molar concentrations of various noniron protoporphyrins. Top left, cobalt protoporphyrin; top center, copper protoporphyrin; top right, nickel protoporphyrin; bottom left, manganese protoporphyrin; bottom center, zinc protoporphyrin; bottom right, gallium protoporphyrin. Ascorbic acid was added as the reducing agent. The spectrum was monitored from 0 min (red dashed line) to 60 min (blue line). The dashed black lines represent each protoporphyrin tested alone. Download FIG S2, TIF file, 0.6 MB. Copyright © 2017 Lojek et al. 2017 Lojek et al. This content is distributed under the terms of the Creative Commons Attribution 4.0 International license.

LFO1 contains protein sequence similarities to both antibiotic biosynthesis monooxygenases and members of the IsdG family of heme oxygenases, which have been also been identified as monooxygenases (38, 40). However, other non-oxygen-dependent heme-degrading enzymes have also been recently identified (15). To test whether LFO1 uses oxygen as a substrate in heme degradation, heme degradation reactions were performed in an oxygraph, allowing detection of the rate of oxygen consumption. When LFO1 was bound to heme in the absence of ascorbate, the rate of oxygen consumption was 6.626 ± 0.991 nmol of oxygen/μl/min, whereas after ascorbate was added and degradation of heme commenced, LFO1 utilized 19.994 ± 0.116 nmol of oxygen/μl/min (Fig. 2C). This rate of oxygen incorporation is similar to that of IsdI (18.366 ± 3.951 nmol of oxygen/μl/min) and yet is statistically different from the rate of oxygen incorporation of heme alone (7.904 ± 0.639 nmol of oxygen/μl/min) (Fig. 2C). This shows that LFO1 utilizes oxygen during heme degradation and does so at a rate similar to that seen with other heme monooxygenases.

### IsdG family heme oxygenase catalytic residues are conserved and important for LFO1 function.

In addition to conserved secondary structural characteristics, IsdG family heme oxygenases are also defined by a conserved catalytic triad of amino acids, composed of asparagine (N), tryptophan (W), and histidine (H). These residues are required by all functionally characterized IsdG family heme oxygenases to degrade heme (22). LFO1 was aligned to protein sequences of seven functionally characterized IsdG family members, revealing the presence of the conserved catalytic triad (Fig. 3A). To determine if LFO1 catalyzes heme degradation similarly to other IsdG family heme oxygenases, these residues were individually changed to alanine. Mutation of the conserved asparagine (N41A) (Fig. 3C), tryptophan (W100A) (Fig. 3D), or histidine (H110A) (Fig. 3E) decreased the ability of LFO1 to degrade heme. This finding indicates that the ability of LFO1 to degrade heme is dependent on these residues. Interestingly, there are three additional conserved residues across the IsdG family heme oxygenases that are also contained within LFO1: F69, G70, and W91. When these residues were mutated to alanines, the G70A and W91A mutants were unable to degrade heme (Fig. S3). Therefore, the LFO1 enzyme has both the predicted secondary structure and the conserved triad of catalytic residues of IsdG family heme oxygenases.

10.1128/mSphere.00176-17.3 FIG S3  Two IsdG conserved noncatalytic residues are required for heme degradation by LFO1. Heme degradation reactions were performed in the presence of catalase with wild-type LFO1 or LFO1 with point mutations in each of the conserved residues G69A, F70A, and W91A. The spectrum was monitored from 0 min (red line) to 60 min (blue line), with data obtained every 5 minutes (black lines). Download FIG S3, TIF file, 0.5 MB. Copyright © 2017 Lojek et al. 2017 Lojek et al. This content is distributed under the terms of the Creative Commons Attribution 4.0 International license.

### LFO1 degrades heme to a distinct heme catabolite.

**FIG 4:**
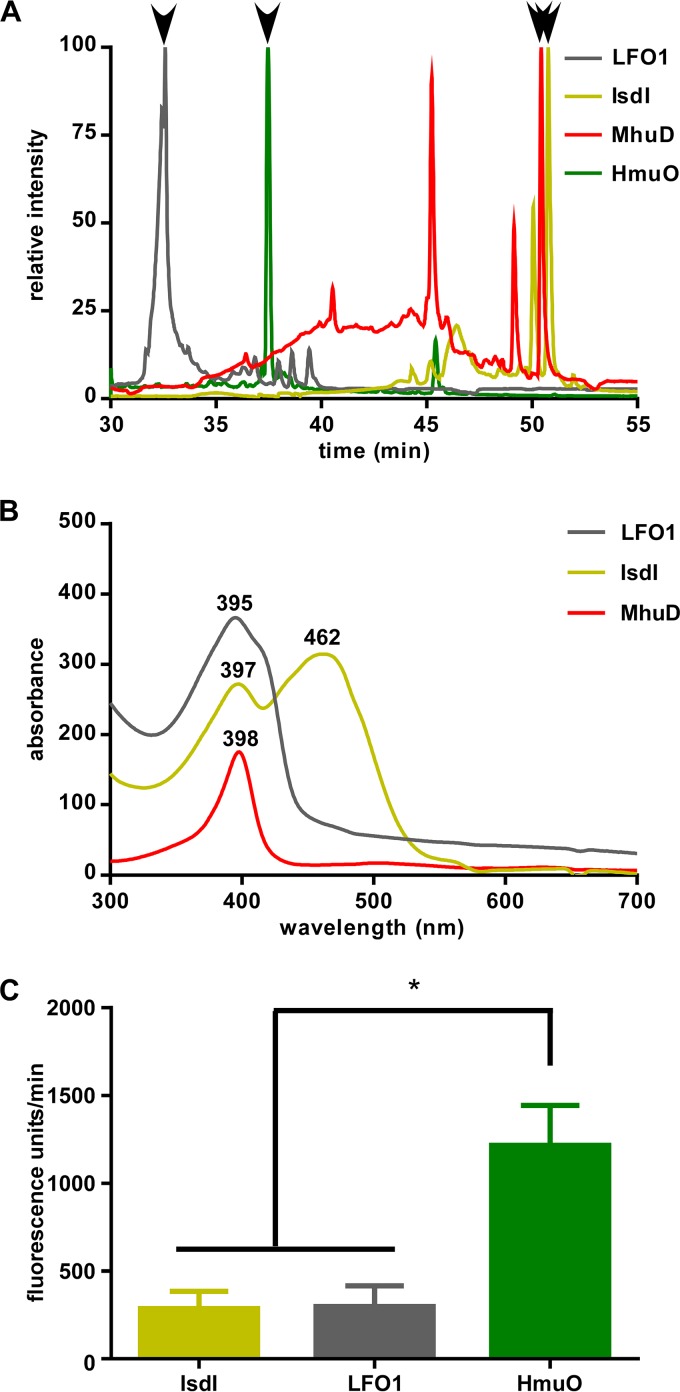
LFO1 degrades heme to a distinct catabolite. (A) HPLC chromatograms of mycobilin from MhuD heme degradation (red), biliverdin from HmuO heme degradation (green), staphylobilin from Isdl heme degradation (yellow), and the LFO1 catabolite (gray) monitored at an absorbance wavelength of 400 nm, graphed with the major peak set to 100. The major peak is indicated for each sample with a black arrow. The predominant peak for the LFO1 catabolite corresponds to a retention time of 32.57 min. (B) Spectra were extracted for each of the samples at the retention times of their peak maxima. The LFO1 catabolite showed a peak absorbance at 395 nm and 32.57 min. (C) Rates of fluorescence (expressed in units per minute) acquired by incubating the heme degradation reaction mixtures of Isdl (yellow), LFO1 (gray), and HmuO (green) in the presence of COP-1, a reaction-based probe which fluoresces selectively in the presence of carbon monoxide (*P* = 0.0330).

To determine if the products of the LFO1 heme degradation reaction were consistent with previously identified heme degradation products, the LFO1 heme degradation reaction was purified and analyzed by high-performance liquid chromatography (HPLC). The retention time and spectrum of the LFO1 heme catabolite were compared to those of staphylobilin, biliverdin, and mycobilin. The retention time of the LFO1 product is ~32.5 min (Fig. 4A), which is distinct from the retention times of known heme oxygenase products. Heme was also run as a standard for comparison; however, heme has a retention time greater than that of all of the heme catabolites (~60 min). Additionally, the LFO1 degradation product had a unique absorbance spectrum in comparison to those of mycobilin and staphylobilin (Fig. 4B).

One of the products of HO-1 family heme degradation is carbon monoxide. Utilizing COP-1, a reaction-based probe which fluoresces selectively in the presence of carbon monoxide (41), the ability of LFO1 to produce carbon monoxide as a result of heme degradation was determined. When HmuO from *Corynebacterium diphtheriae* degraded heme in the presence of COP-1, the rate of fluorescence increase was 1,215.6 ± 213.1 fluorescence units/min. When LFO1 degraded heme in the presence of COP-1, the rate of fluorescence production was significantly less than that seen with HmuO (297.8 ± 117.6 fluorescence units/min) and was more comparable to the rate of fluorescence seen with IsdI (285.3 ± 98.6 fluorescence units/min) (Fig. 4C). This indicates that LFO1 does not release carbon monoxide upon degradation of heme, confirming that LFO1 is not an HO-1 family heme oxygenase.

### The members of the IsdG family of heme oxygenases are distributed across all domains of life.

A phylogenetic tree was previously created using the first identified member of the IsdG family of heme oxygenases, IsdG from *Staphylococcus aureus*, as a seed (16). This tree identified a total of 22 proteins as IsdG family members. However, characterization of LFO1 as an IsdG family member showed that this tree does not sufficiently encapsulate all potential IsdG family heme oxygenases. Our search identified 1,204 sequences homologous to characterized IsdG proteins, 866 of which contained the conserved NWH catalytic triad and are referred to here as IsdG-like proteins (Table S1). The 852 bacterial IsdG-like proteins were distributed across eight bacterial divisions, with the majority found in *Actinobacteria* (317 proteins), *Proteobacteria* (292 proteins), and *Firmicutes* (206 proteins) (Table S1). Our search also identified four archaeal IsdG-like proteins, one from *Crenarchaeota* and three from *Euryarchaeota*. In addition to LFO1 from *C. reinhardtii*, our search identified nine IsdG-like proteins in eukaryotes, all from other photosynthetic species, including the colonial green alga *Volvox cateri*, prasinophyte green algae in the genera *Ostreococcus* and *Micromonas*, diatoms in the genera *Fragilariopsis* and *Thalassiosira*, and the chromerid *Vitrella brassicaformis* (Table S1).

10.1128/mSphere.00176-17.4 Table S1  IsdG family proteins. Download Table S1, DOCX file, 0.2 MB. Copyright © 2017 Lojek et al. 2017 Lojek et al. This content is distributed under the terms of the Creative Commons Attribution 4.0 International license.

**FIG 5:**
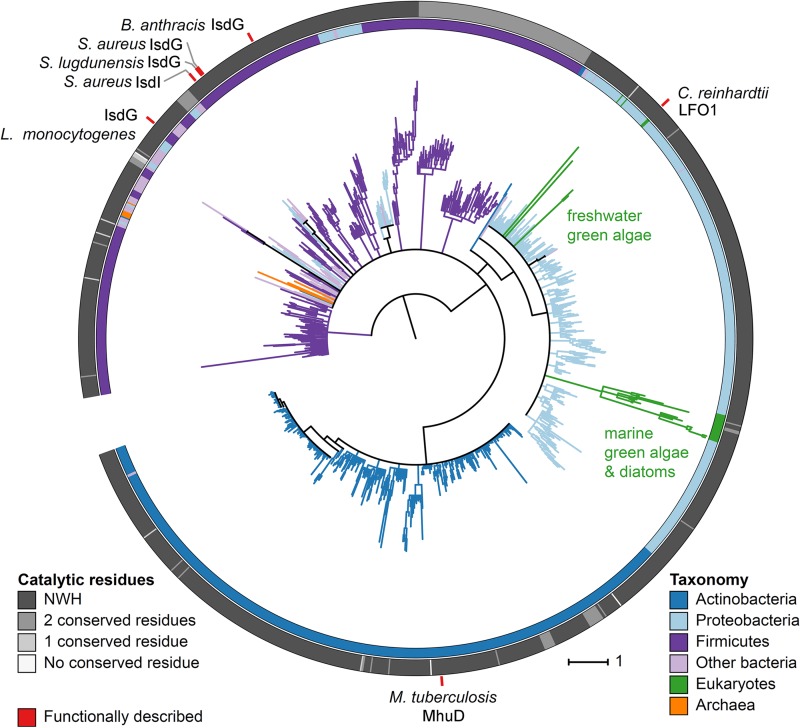
The members of the IsdG family of heme oxygenases are widely distributed in bacteria and are present in all three domains of life. Data represent a maximum likelihood phylogeny of the IsdG heme oxygenase protein family. The tree was rooted at the midpoint and visualized using iTOL version 3.0. Color strips along the tree perimeter correspond to the taxonomy (inner strip) and the number of conserved IsdG family catalytic residues contained within each protein sequence (outer strip). Red tick marks indicate the IsdG family enzymes that were previously functionally characterized.

To determine the evolutionary history of LFO1, we constructed a maximum likelihood phylogeny of IsdGs and homologs (Fig. 5). All the eukaryotic sequences, including the LFO1 sequence, grouped within a large proteobacterial clade of IsdG-like proteins. *C. reinhardtii* and *V. cateri*, the two species of freshwater green algae, grouped with each other but not with the marine prasinophyte green algae, which instead grouped with marine diatoms (Fig. 5). The small number of eukaryotic IsdG-like sequences and the fact that they do not form a single monophyletic group suggest that these sequences may have been acquired from bacteria via horizontal gene transfer. However, the low average level of sequence similarity and minimal length of this protein resulted in a phylogeny with weak branch support, which makes it challenging or even impossible to infer the number and directionality of possible gene transfer events.

Interestingly, there are multiple organisms that were identified by these analyses that have previously been described to contain HO-1 family heme oxygenases. This suggests that *C. reinhardtii* is not unique in harboring two distinct families of heme oxygenases.

## DISCUSSION

The members of the HO-1 family of heme oxygenases degrade heme to the products biliverdin, carbon monoxide, and free iron (5). However, the discovery of the IsdG family indicates that heme can also be degraded to different bilin products (17). These bilin products could function in as-yet-undiscovered light signaling or host-microbe signaling pathways. By continuing to explore this family of heme oxygenases, a protein from *C. reinhardtii*, LFO1, was identified. LFO1 has a predicted secondary structure similar to the conserved secondary structure seen in IsdG family heme oxygenases and distinct from the secondary structure of the HO-1 family (Fig. 1). Functional characterization of LFO1 showed that it is able to both bind and degrade heme (Fig. 2) and that this heme degradation is dependent on the presence of three catalytic residues which are conserved in all IsdG family members (Fig. 3). While HO-1 family heme oxygenases produce biliverdin and carbon monoxide, the members of the IsdG family of heme oxygenases produce multiple products, including staphylobilin and mycobilin. Interestingly, although we were unable to structurally define the product of LFO1-dependent heme catabolism, it appears that LFO1 produces a molecule distinct from the bilin degradation products of previously described heme oxygenases (Fig. 4).

IsdG family heme oxygenases have been functionally characterized in seven bacterial species (13, 16–18, 20, 21), and only 22 enzymes have been predicted to be IsdG family members by phylogenetic analysis (16). However, given the low sequence identity between IsdG family members, it is not surprising that the previous phylogenetic analysis missed enzymes with potentially similar functionalities. Focusing on the secondary structure and the conservation of the catalytic triad allowed identification of LFO1 as a eukaryotic enzyme that belongs to the IsdG family of heme oxygenases. Using the sequence of LFO1 as a seed, a new phylogenetic analysis was completed. This sequence analysis identified 866 species containing IsdG family members, indicating that IsdG heme oxygenases are more widely distributed than previously appreciated (Fig. 5).

*Chlamydomonas reinhardtii* contains HMOX1 and HMOX2, two HO-1 family heme oxygenases (42, 43), and LFO1, an IsdG family heme oxygenase. Interestingly, RNA sequencing data from *C. reinhardtii* showed that *Lfo1* is differentially expressed as a function of iron nutrition, such that *Lfo1* is upregulated under low-iron conditions and downregulated under conditions of iron excess (25). This regulation of LFO1 is similar to that of bacterial IsdG family members, where the heme oxygenases are important for iron scavenging from heme. However, HMOX1 within the chloroplast and HMOX2 within the cytoplasm are not differentially expressed under these conditions (25). Photosynthesis is a highly iron-dependent process, requiring approximately 30 iron atoms for linear electron flow (44). Therefore, many iron-responsive acclimatory processes in *C. reinhardtii* involve the photosynthetic complexes. For instance, if provided a fixed carbon source (acetate), *C. reinhardtii* sacrifices nonessential iron-dependent proteins in the chloroplast, such as cytochrome *b_6_f*, photosystem I (PSI), and ferredoxin to spare iron and recycle this cofactor to essential iron-dependent proteins (45). Cytochrome *b_6_f*, in particular, contains eight hemes per functional dimer (46), suggesting that degradation of this complex during iron-limited photoheterotrophic growth results in a bolus of heme in the chloroplast which must be degraded to prevent heme toxicity and to enable iron recycling. The identification of LFO1 as an iron-regulated heme oxygenase suggests that LFO1 may play a role in heme degradation in order to release its iron to aid in the acclimation of *C. reinhardtii* to low-iron conditions. By producing a degradation product that is unique in comparison to those produced by the members of the HO-1 family, LFO1 would be able to recycle heme during Fe deficiency without eliciting the bilin retrograde signaling pathway that involves HMOX1 in the *Chlamydomonas* chloroplast (43).

## MATERIALS AND METHODS

### Bacterial strains and growth.

Immunoblotting, transformations, plasmid purification, and subcloning were carried out as described previously (47). Anti-His rabbit polyclonal antibody was purchased from Santa Cruz. *Escherichia coli* strain DH5α was used for DNA manipulation, and BL21(DE3) was used for protein expression of LFO1, IsdI, MhuD, and HmuO. The production of BL21(DE3) pET15b.*isdI* was previously described (17). Ampicillin (100 μg/ml) and chloramphenicol (34 μg/ml) were added to the media as required. All strains were incubated at 37°C with shaking.

### Construction of vectors.

To create vectors to be used in expression, *Lfo1* was codon optimized for expression in *E. coli* and was synthesized into pUC (GenScript). *Lfo1* was cleaved from pUC using BamHI-HF and NdeI (New England Biolabs) and ligated into pET15b.

### Expression and purification of LFO1, IsdI, MhuD, and HmuO.

*E. coli* BL21(DE3) pREL strains containing pET15b.*Lfo1*, pET15b.*isdI*, pET15b.*mhuD*, or pET15b.*hmuO* were grown overnight at 37°C in Terrific broth containing 100 μg/ml ampicillin and 34 μg/ml chloramphenicol. The cells were subcultured into fresh medium and grown at 37°C to mid-log phase at an optical density at 600 nm (OD_600_) of 0.4 to 0.6. Upon reaching mid-log growth, the target genes were induced using 0.1 mM isopropyl-1-thiol-d-galactopyranoside (IPTG). Cell growth was continued for approximately 16 h at 16°C. Cells were harvested by centrifugation (6,000 × *g* for 15 min). Cells were resuspended in lysis buffer (8 M urea, phosphate-buffered saline [PBS], 5 mM dithiothreitol [DTT], one Pierce protease inhibitor tablet [Thermo Scientific]) and mixed at room temperature for 30 min. Cells were homogenized using a Dounce homogenizer and passed through an EmulsiFlex homogenizer (Avestin) three times at 20,000 lb/in^2^. After lysis, lysates were mixed at room temperature for 30 min. Lysate was centrifuged at 40,000 × *g* for 1 h and filtered with a 0.22-μM-pore-size filter. The filtered lysate was added to nickel-nitrilotriacetic acid (Ni-NTA) agarose beads and was incubated at room temperature with rotation for 30 min. The lysate and beads were poured into a gravity column. The column was washed with lysis buffer with stepwise decreases in urea from 8 to 0 M. The column was then washed with 10 column volumes of 5 mM DTT–100 mM imidazole–PBS. Proteins were eluted with 400 mM imidazole in lysis buffer in 5 column volumes. Proteins were used immediately after purification.

### Absorption spectroscopy.

All absorption spectra were obtained using a Varian Cary 50BIO. Hemin from bovine was procured from Sigma-Aldrich and resuspended in 0.1 M sodium hydroxide. Hemin binding studies were performed as previously reported (6). Specifically, aliquots of hemin at concentrations of 0.5 to 30 μM were added to the sample cuvette in a reaction mixture containing 10 μM LFO1 and 1 ml of Tris-buffered saline (TBS) and to reference cuvettes containing 1 ml of TBS at room temperature. Each sample was mixed and allowed to incubate in the dark for 5 min, and then the spectrum was collected at 300 to 800 nm with 10-nm steps.

### Hemin degradation assays.

Hemin degradation assays were performed as previously described (17, 35). For the reactions performed with ascorbate, 10 μM LFO1–hemin–TBS was specifically incubated with ascorbate at a final concentration of 10 mM (9). The spectral changes between 300 and 800 nm were recorded every 10 min. The products were extracted and subjected to HPLC as described below. For the reactions performed with catalase, purified recombinant catalase from *Aspergillus niger* (Sigma) was added to all reaction cuvettes at a catalase/hemoprotein ratio equal to 1:2 immediately before the addition of ascorbate.

### Oxygen incorporation.

The consumption of oxygen was determined by using a Gilson model 5/6 oxygraph (Gilson Medical Electronics, Inc., Middleton, WI) set at 24°C and equipped with a Clark electrode and a thermostated cuvette. Triplicate samples of LFO1, IsdI, or no protein at 10 μM were added to TBS with 10 μM heme and 5 μM catalase. A 1-ml volume of each sample was added to the oxygraph, and oxygen consumption was measured for 1 min. Degradation was then initialized by adding 10 mM ascorbate, and oxygen uptake was measured for an additional 4 min. The data corresponding to the velocity of the oxygen consumption were determined from the first 1 min after ascorbate addition and were all within the linear portion of the curve. The rates determined for triplicate samples were averaged, and the averages and standard errors of the means (SEM) were graphed.

### HPLC of the heme degradation reaction product.

HPLC analysis was performed as previously described (12). Specifically, analysis was performed using 95% water–5% acetonitrile–0.1% trifluoroacetic acid (TFA) as the mobile phase with a flow rate of 0.5 ml/min on a Microsorb-MV C_18_ column. After a 10-min equilibration period, a linear 40-min acetonitrile gradient (5% to 80%) was employed and the final concentration was maintained for an additional 20 min. The eluate was monitored using a photodiode array detector from 200 to 900 nm by reverse-phase chromatography on a Varian ProStar HPLC instrument.

### COP-1 fluorescence detection.

COP-1 was utilized as previously described (41). HmuO, IsdI, and LFO1 at 10 μM were preincubated with 10 μM hemin for 5 min. Aliquots of 200 μl of the hemin-bound proteins were transferred into a black-well 96-well plate. Reaction mixtures were incubated with 1 μM COP-1, and background fluorescence was measured at 510 nm. A final concentration of 1 mM ascorbate was then added to each sample, the reaction mixtures were incubated for 5 min, and measurement of the fluorescence at 510 nm was performed every 2 min for 30 min.

### Heme oxygenase phylogenetic analysis.

The 6 functionally characterized IsdG protein sequences from bacteria were queried against the UniProt reference proteomes using phmmer, a member of the HMMER3 software suite (48) (Web server accessed 27 March 2017), and the full-length sequence for each significant hit was downloaded for further analysis (sequence *E* value cutoff, 0.01; hit *E* value cutoff, 0.03). Downloaded sequences were filtered based on sequence composition and to reduce redundancy in the data set using a custom perl script as well as IQ-TREE (49) (Table S1). Eukaryotic sequences containing two ABM domains were manually split into their individual domains and accordingly labeled “CTERM” or “NTERM” (Table S1). Filtered sequences were aligned using MAFFT v7.023b and the E-INS-I strategy (50). The topologies were inferred using maximum likelihood as implemented in IQ-TREE version 1.3.8 and an LG+G4 substitution model (automatically determined to be the best model within IQ-TREE using the Bayesian information criterion) and ultrafast bootstrapping (1,000 replications) (49). The phylogenies were midpoint rooted and visualized using iTOL version 3.0 (51). All trees and alignments are available from the figshare repository (https://doi.org/10.6084/m9.figshare.4810165.v1; last accessed 31 March 2017).
