# Supplementary figures and images for: Chlamydomonas reinhardtii LFO1 Is an IsdG Family Heme Oxygenase
Source: mSphere. 2017 Aug 16;2(4):e00176-17. doi: 10.1128/mSphere.00176-17 (PMC5557675; doi:10.1128/mSphere.00176-17)

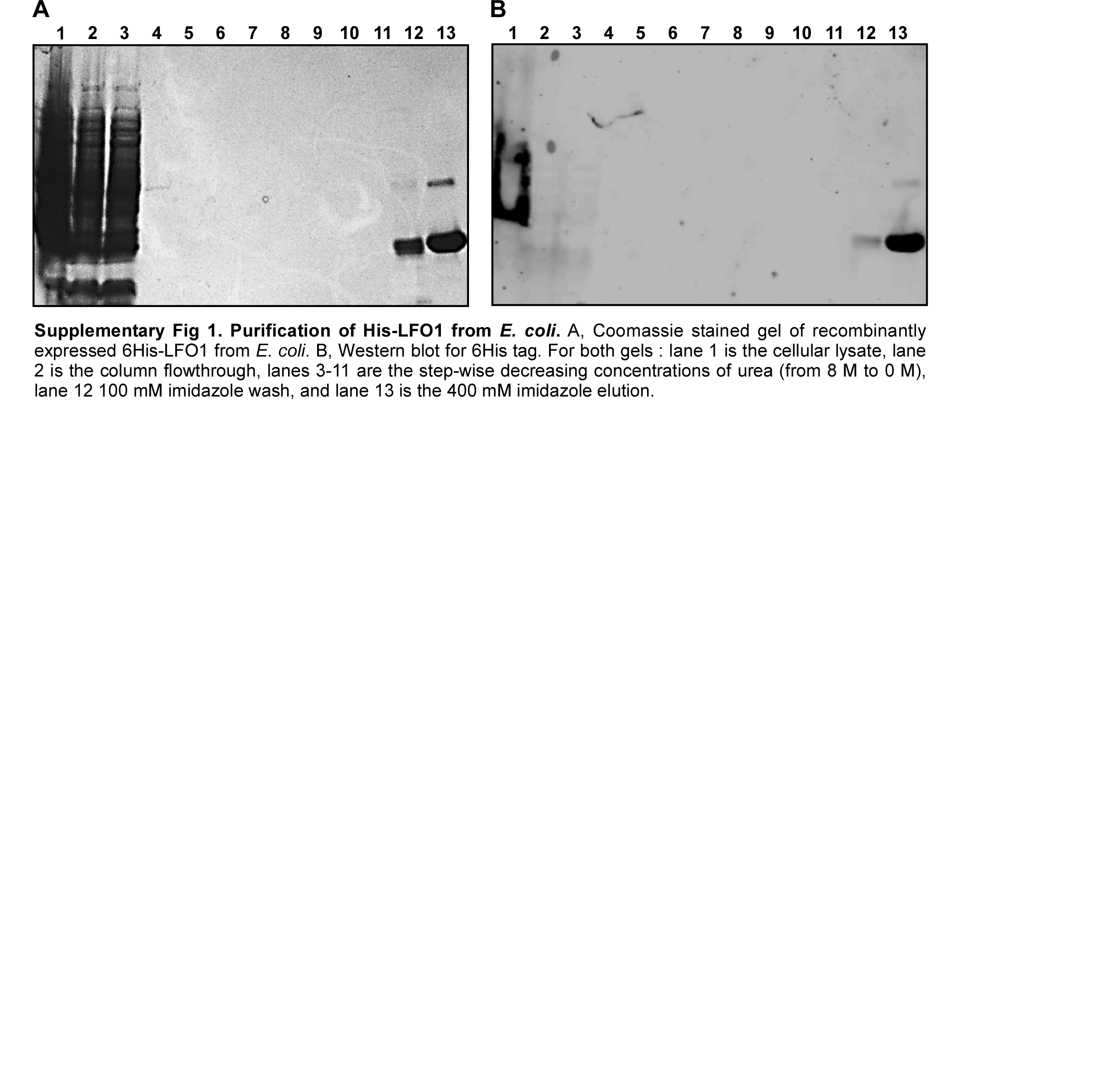

Supplement: FIG S1 [file sph004172341sf1.tif]

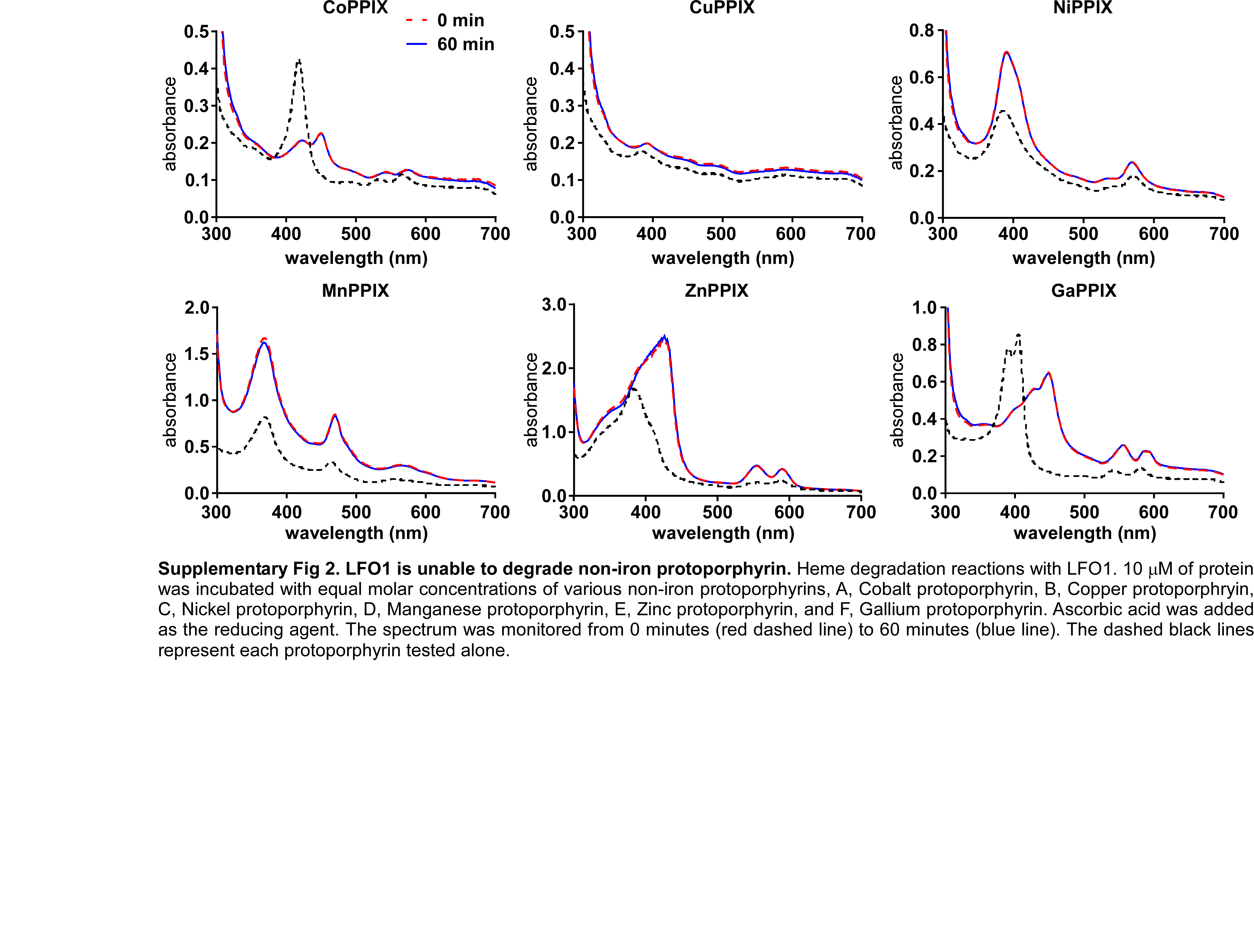

Supplement: FIG S2 [file sph004172341sf2.tif]

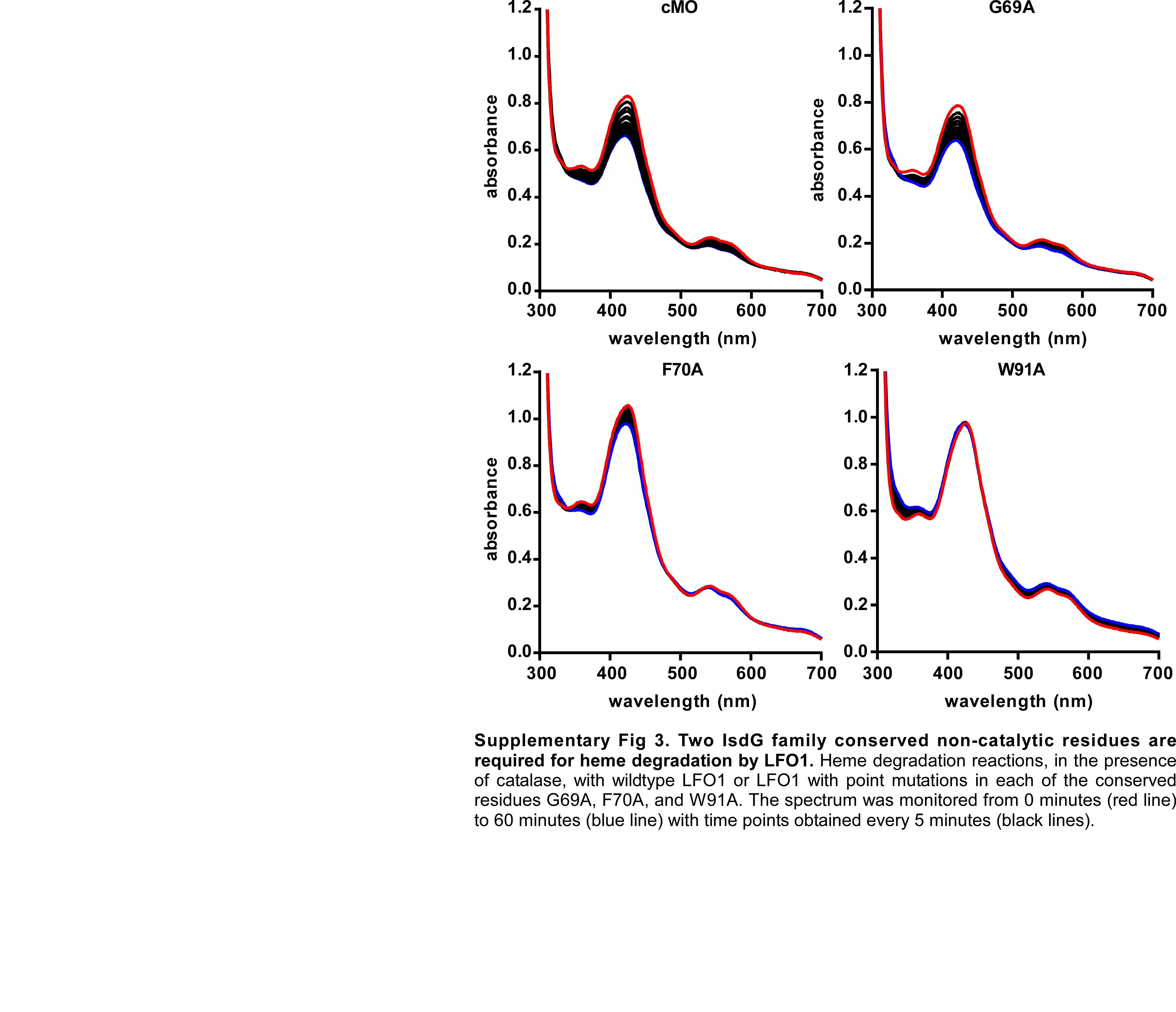

Supplement: FIG S3 [file sph004172341sf3.tif]
