# Supplementary material for: Chlamydomonas reinhardtii LFO1 Is an IsdG Family Heme Oxygenase
Source: mSphere. 2017 Aug 16;2(4):e00176-17. doi: 10.1128/mSphere.00176-17 (PMC5557675; doi:10.1128/mSphere.00176-17)
Supplement: Table S1 [file sph004172341st4.docx]

**Supplementary Table 1. IsdG Family Proteins.**

Sequences identified to be homologous to characterized IsdG family proteins.

| **sequence id** | **domain** | **lineage** | **taxon** | **taxonomy**  **id** | **conserved**  **residues** | **functionally characterized** | **notes** |
| --- | --- | --- | --- | --- | --- | --- | --- |
| MHUD_MYCTU | Bacteria | Actinobacteria | Mycobacterium_  tuberculosis | 83332 | 3(NWH) | MhuD |  |
| X8F931_MYCUL | Bacteria | Actinobacteria | Mycobacterium_  ulcerans | 1809 | 0(---) |  |  |
| A0A0K9EV67_9ACTO | Bacteria | Actinobacteria | Actinomycetales | 2037 | 0(CYP) |  |  |
| D2NSQ3_ROTMD | Bacteria | Actinobacteria | Rothia_  mucilaginosa | 680646 | 1(HYH) |  |  |
| B5HBJ5_STRPR | Bacteria | Actinobacteria | Streptomyces_  pristinaespiralis | 38300 | 1(N--) |  |  |
| A0A0M9ZLZ8_9ACTN | Bacteria | Actinobacteria | Actinobacteria | 201174 | 2(-WH) |  |  |
| A0A0M9YDN3_9ACTN | Bacteria | Actinobacteria | Actinobacteria | 201174 | 2(-WH) |  |  |
| A0A0M8Y9Z8_9ACTN | Bacteria | Actinobacteria | Actinobacteria | 201174 | 2(-WH) |  |  |
| G0HEU7_CORVD | Bacteria | Actinobacteria | Corynebacterium_  variabile | 858619 | 2(-WH) |  |  |
| J9SBJ3_9ACTN | Bacteria | Actinobacteria | Actinobacteria | 201174 | 2(-WH) |  |  |
| C7MAB6_BRAFD | Bacteria | Actinobacteria | Brachybacterium_  faecium | 446465 | 2(NWP) |  |  |
| A0A0D1L2H3_9MYCO | Bacteria | Actinobacteria | Mycobacteriaceae | 1762 | 2(NWP) |  |  |
| U7MIJ1_9CORY | Bacteria | Actinobacteria | Corynebacteriaceae | 1653 | 2(SWH) |  |  |
| G7GLA8_9ACTN | Bacteria | Actinobacteria | Actinobacteria | 201174 | 2(SWH) |  |  |
| U7L1T3_9CORY | Bacteria | Actinobacteria | Corynebacteriaceae | 1653 | 2(TWH) |  |  |
| I6WRW4_PSEPQ | Bacteria | Actinobacteria | Pseudopropionibacterium_propionicum | 767029 | 2(TWH) |  |  |
| A0A0M4GQF6_9ACTO | Bacteria | Actinobacteria | Actinomycetales | 2037 | 2(TWH) |  |  |
| U1RK52_9ACTO | Bacteria | Actinobacteria | Actinomycetales | 2037 | 2(TWH) |  |  |
| E7NDT5_9ACTO | Bacteria | Actinobacteria | Actinomycetales | 2037 | 2(TWH) |  |  |
| F9PJ40_9ACTO | Bacteria | Actinobacteria | Actinomycetales | 2037 | 2(TWH) |  |  |
| F9EHX2_9ACTO | Bacteria | Actinobacteria | Actinomycetales | 2037 | 2(TWH) |  |  |
| J0N3K2_9ACTO | Bacteria | Actinobacteria | Actinomycetales | 2037 | 2(TWH) |  |  |
| F5XN95_MICPN | Bacteria | Actinobacteria | Microlunatus_  phosphovorus | 1032480 | 2(TWH) |  |  |
| A0A096AGV7_9MICC | Bacteria | Actinobacteria | Micrococcaceae | 1268 | 2(TWH) |  |  |
| Q8NMA1_CORGL | Bacteria | Actinobacteria | Corynebacterium_  glutamicum | 196627 | 2(TWH) |  |  |
| A0A0X8JD09_9ACTO | Bacteria | Actinobacteria | Actinomycetales | 2037 | 2(TWH) |  |  |
| A0A023X207_9ACTN | Bacteria | Actinobacteria | Actinobacteria | 201174 | 3(NWH) |  |  |
| A0A151C0Z2_9MICO | Bacteria | Actinobacteria | Micrococcales | 85006 | 3(NWH) |  |  |
| A0A0Q7KDM5_9ACTN | Bacteria | Actinobacteria | Actinobacteria | 201174 | 3(NWH) |  |  |
| E2SE28_9ACTN | Bacteria | Actinobacteria | Actinobacteria | 201174 | 3(NWH) |  |  |
| A0A0Q6FJW8_9ACTN | Bacteria | Actinobacteria | Actinobacteria | 201174 | 3(NWH) |  |  |
| R7XTL6_9ACTN | Bacteria | Actinobacteria | Actinobacteria | 201174 | 3(NWH) |  |  |
| C8XE02_NAKMY | Bacteria | Actinobacteria | Nakamurella_  multipartita | 479431 | 3(NWH) |  |  |
| A8KZJ6_FRASN | Bacteria | Actinobacteria | Frankia_sp | 298653 | 3(NWH) |  |  |
| A0A099J2D7_9MICO | Bacteria | Actinobacteria | Micrococcales | 85006 | 3(NWH) |  |  |
| N1V167_9MICC | Bacteria | Actinobacteria | Micrococcaceae | 1268 | 3(NWH) |  |  |
| A0A0K1JL49_9MICO | Bacteria | Actinobacteria | Micrococcales | 85006 | 3(NWH) |  |  |
| A0A0U2WW81_9MICC | Bacteria | Actinobacteria | Micrococcaceae | 1268 | 3(NWH) |  |  |
| A0A0M2CTM9_9MICC | Bacteria | Actinobacteria | Micrococcaceae | 1268 | 3(NWH) |  |  |
| A0A010NNJ8_9MICC | Bacteria | Actinobacteria | Micrococcaceae | 1268 | 3(NWH) |  |  |
| A0A1E8F3N9_9MICC | Bacteria | Actinobacteria | Micrococcaceae | 1268 | 3(NWH) |  |  |
| A0A0C1RCG9_9ACTN | Bacteria | Actinobacteria | Actinobacteria | 201174 | 3(NWH) |  |  |
| A0A0C1UUQ3_9ACTN | Bacteria | Actinobacteria | Actinobacteria | 201174 | 3(NWH) |  |  |
| A0A0G3V296_9ACTN | Bacteria | Actinobacteria | Actinobacteria | 201174 | 3(NWH) |  |  |
| D1ABQ2_THECD | Bacteria | Actinobacteria | Thermomonospora_  curvata | 471852 | 3(NWH) |  |  |
| D6Y690_THEBD | Bacteria | Actinobacteria | Thermobispora_  bispora | 469371 | 3(NWH) |  |  |
| A0A0Q5VMD4_9ACTN | Bacteria | Actinobacteria | Actinobacteria | 201174 | 3(NWH) |  |  |
| A0A101NH48_9ACTN | Bacteria | Actinobacteria | Actinobacteria | 201174 | 3(NWH) |  |  |
| H2K7F8_STRHJ | Bacteria | Actinobacteria | Streptomyces_  hygroscopicus subsp | 1133850 | 3(NWH) |  |  |
| S5VJM8_STRC3 | Bacteria | Actinobacteria | Streptomyces_  collinus | 1214242 | 3(NWH) |  |  |
| A0A0M8XI27_9ACTN | Bacteria | Actinobacteria | Actinobacteria | 201174 | 3(NWH) |  |  |
| S4N0J3_9ACTN | Bacteria | Actinobacteria | Actinobacteria | 201174 | 3(NWH) |  |  |
| A0A0G3ADB2_9ACTN | Bacteria | Actinobacteria | Actinobacteria | 201174 | 3(NWH) |  |  |
| A0A089XCB7_STRGA | Bacteria | Actinobacteria | Streptomyces_  glaucescens | 1907 | 3(NWH) |  |  |
| A0A0D4DU44_9ACTN | Bacteria | Actinobacteria | Actinobacteria | 201174 | 3(NWH) |  |  |
| A0A0X3XUH2_9ACTN | Bacteria | Actinobacteria | Actinobacteria | 201174 | 3(NWH) |  |  |
| A0A101J6R4_9ACTN | Bacteria | Actinobacteria | Actinobacteria | 201174 | 3(NWH) |  |  |
| K4QXE6_9ACTN | Bacteria | Actinobacteria | Actinobacteria | 201174 | 3(NWH) |  |  |
| A0A101PFB4_9ACTN | Bacteria | Actinobacteria | Actinobacteria | 201174 | 3(NWH) |  |  |
| A0A176L9W5_9ACTN | Bacteria | Actinobacteria | Actinobacteria | 201174 | 3(NWH) |  |  |
| A0A0N0N865_9ACTN | Bacteria | Actinobacteria | Actinobacteria | 201174 | 3(NWH) |  |  |
| A0A086MSV8_9ACTN | Bacteria | Actinobacteria | Actinobacteria | 201174 | 3(NWH) |  |  |
| A0A177HVH0_9ACTN | Bacteria | Actinobacteria | Actinobacteria | 201174 | 3(NWH) |  |  |
| A0A0B5D7R1_9ACTN | Bacteria | Actinobacteria | Actinobacteria | 201174 | 3(NWH) |  |  |
| A0A0X3SKW4_9ACTN | Bacteria | Actinobacteria | Actinobacteria | 201174 | 3(NWH) |  |  |
| A0A0C5GDZ5_9ACTN | Bacteria | Actinobacteria | Actinobacteria | 201174 | 3(NWH) |  |  |
| A0A0N1GRV6_9ACTN | Bacteria | Actinobacteria | Actinobacteria | 201174 | 3(NWH) |  |  |
| A0A1K2FHG3_9ACTN | Bacteria | Actinobacteria | Actinobacteria | 201174 | 3(NWH) |  |  |
| A0A1J4Q6M5_9ACTN | Bacteria | Actinobacteria | Actinobacteria | 201174 | 3(NWH) |  |  |
| A0A1D7VH98_9ACTN | Bacteria | Actinobacteria | Actinobacteria | 201174 | 3(NWH) |  |  |
| J1ZT77_9ACTN | Bacteria | Actinobacteria | Actinobacteria | 201174 | 3(NWH) |  |  |
| A0A059W6D6_STRA9 | Bacteria | Actinobacteria | Streptomyces_  albulus | 68570 | 3(NWH) |  |  |
| A0A0U3NP43_9ACTN | Bacteria | Actinobacteria | Actinobacteria | 201174 | 3(NWH) |  |  |
| D9X4M1_STRVT | Bacteria | Actinobacteria | Streptomyces_  viridochromogenes | 591159 | 3(NWH) |  |  |
| A0A0M8VNK7_9ACTN | Bacteria | Actinobacteria | Actinobacteria | 201174 | 3(NWH) |  |  |
| V6KE10_STRRC | Bacteria | Actinobacteria | Streptomyces_  roseochromogenus subsp | 149682 | 3(NWH) |  |  |
| A0A124I2D5_9ACTN | Bacteria | Actinobacteria | Actinobacteria | 201174 | 3(NWH) |  |  |
| A0A0M3QL09_9ACTN | Bacteria | Actinobacteria | Actinobacteria | 201174 | 3(NWH) |  |  |
| B1VNT2_STRGG | Bacteria | Actinobacteria | Streptomyces_  griseus subsp | 455632 | 3(NWH) |  |  |
| A0A1J4P5W0_9ACTN | Bacteria | Actinobacteria | Actinobacteria | 201174 | 3(NWH) |  |  |
| A0A022MCA4_9ACTN | Bacteria | Actinobacteria | Actinobacteria | 201174 | 3(NWH) |  |  |
| G2GFM1_9ACTN | Bacteria | Actinobacteria | Actinobacteria | 201174 | 3(NWH) |  |  |
| A0A171BH93_9ACTN | Bacteria | Actinobacteria | Actinobacteria | 201174 | 3(NWH) |  |  |
| A0A101T4I1_9ACTN | Bacteria | Actinobacteria | Actinobacteria | 201174 | 3(NWH) |  |  |
| A0A0Q9AQ68_9ACTN | Bacteria | Actinobacteria | Actinobacteria | 201174 | 3(NWH) |  |  |
| D6K8V7_9ACTN | Bacteria | Actinobacteria | Actinobacteria | 201174 | 3(NWH) |  |  |
| A0A0L0JWV0_9ACTN | Bacteria | Actinobacteria | Actinobacteria | 201174 | 3(NWH) |  |  |
| A0A0F4K7B4_9ACTN | Bacteria | Actinobacteria | Actinobacteria | 201174 | 3(NWH) |  |  |
| A0A0F4JK55_9ACTN | Bacteria | Actinobacteria | Actinobacteria | 201174 | 3(NWH) |  |  |
| A0A0M8TCY0_9ACTN | Bacteria | Actinobacteria | Actinobacteria | 201174 | 3(NWH) |  |  |
| D9VU91_9ACTN | Bacteria | Actinobacteria | Actinobacteria | 201174 | 3(NWH) |  |  |
| A0A1E5Q1S4_9ACTN | Bacteria | Actinobacteria | Actinobacteria | 201174 | 3(NWH) |  |  |
| A0A0C1SA86_9ACTN | Bacteria | Actinobacteria | Actinobacteria | 201174 | 3(NWH) |  |  |
| A0A0F4J8X8_9ACTN | Bacteria | Actinobacteria | Actinobacteria | 201174 | 3(NWH) |  |  |
| A0A0F0H9E0_9ACTN | Bacteria | Actinobacteria | Actinobacteria | 201174 | 3(NWH) |  |  |
| A0A0F2T8A5_9ACTN | Bacteria | Actinobacteria | Actinobacteria | 201174 | 3(NWH) |  |  |
| D9WW83_9ACTN | Bacteria | Actinobacteria | Actinobacteria | 201174 | 3(NWH) |  |  |
| G2P6W6_STRVO | Bacteria | Actinobacteria | Streptomyces_  violaceusniger | 68280 | 3(NWH) |  |  |
| D7CAY2_STRBB | Bacteria | Actinobacteria | Streptomyces_  bingchenggensis | 749414 | 3(NWH) |  |  |
| A0A0K9XMS6_9ACTN | Bacteria | Actinobacteria | Actinobacteria | 201174 | 3(NWH) |  |  |
| A0A0W7XAR1_9ACTN | Bacteria | Actinobacteria | Actinobacteria | 201174 | 3(NWH) |  |  |
| A0A0D0PHL4_KITGR | Bacteria | Actinobacteria | Kitasatospora_  griseola | 2064 | 3(NWH) |  |  |
| E4NAL3_KITSK | Bacteria | Actinobacteria | Kitasatospora_setae | 452652 | 3(NWH) |  |  |
| A0A1E7JKE4_9ACTN | Bacteria | Actinobacteria | Actinobacteria | 201174 | 3(NWH) |  |  |
| C9ZFN2_STRSW | Bacteria | Actinobacteria | Streptomyces_scabiei | 680198 | 3(NWH) |  |  |
| A0A0X3W474_9ACTN | Bacteria | Actinobacteria | Actinobacteria | 201174 | 3(NWH) |  |  |
| A0A0A8ESE7_9ACTN | Bacteria | Actinobacteria | Actinobacteria | 201174 | 3(NWH) |  |  |
| A0A0X3UXH7_9ACTN | Bacteria | Actinobacteria | Actinobacteria | 201174 | 3(NWH) |  |  |
| A0A1B6ATY7_9ACTN | Bacteria | Actinobacteria | Actinobacteria | 201174 | 3(NWH) |  |  |
| L8ELN5_STRRM | Bacteria | Actinobacteria | Streptomyces_  rimosus | 1927 | 3(NWH) |  |  |
| A0A0M8YKA7_9ACTN | Bacteria | Actinobacteria | Actinobacteria | 201174 | 3(NWH) |  |  |
| F2RKU8_STRVP | Bacteria | Actinobacteria | Streptomyces_  venezuelae | 953739 | 3(NWH) |  |  |
| A0A1E5PC13_9ACTN | Bacteria | Actinobacteria | Actinobacteria | 201174 | 3(NWH) |  |  |
| S2XVA0_9ACTN | Bacteria | Actinobacteria | Actinobacteria | 201174 | 3(NWH) |  |  |
| L1KME9_9ACTN | Bacteria | Actinobacteria | Actinobacteria | 201174 | 3(NWH) |  |  |
| S3ZP62_9ACTN | Bacteria | Actinobacteria | Actinobacteria | 201174 | 3(NWH) |  |  |
| A0A081XLA8_STRTO | Bacteria | Actinobacteria | Streptomyces_  toyocaensis | 55952 | 3(NWH) |  |  |
| A0A1D2IA20_9ACTN | Bacteria | Actinobacteria | Actinobacteria | 201174 | 3(NWH) |  |  |
| A0A101V2J2_9ACTN | Bacteria | Actinobacteria | Actinobacteria | 201174 | 3(NWH) |  |  |
| A0A1B1MDL0_STRLN | Bacteria | Actinobacteria | Streptomyces_  lincolnensis | 1915 | 3(NWH) |  |  |
| D5ZPK1_9ACTN | Bacteria | Actinobacteria | Actinobacteria | 201174 | 3(NWH) |  |  |
| A0A1A5P3K7_9ACTN | Bacteria | Actinobacteria | Actinobacteria | 201174 | 3(NWH) |  |  |
| A0A0M8QDV9_9ACTN | Bacteria | Actinobacteria | Actinobacteria | 201174 | 3(NWH) |  |  |
| A0A072SDE8_9ACTN | Bacteria | Actinobacteria | Actinobacteria | 201174 | 3(NWH) |  |  |
| A0A0M8VPX9_9ACTN | Bacteria | Actinobacteria | Actinobacteria | 201174 | 3(NWH) |  |  |
| L7FIY7_9ACTN | Bacteria | Actinobacteria | Actinobacteria | 201174 | 3(NWH) |  |  |
| A0A101R434_9ACTN | Bacteria | Actinobacteria | Actinobacteria | 201174 | 3(NWH) |  |  |
| V4IJQ8_9ACTN | Bacteria | Actinobacteria | Actinobacteria | 201174 | 3(NWH) |  |  |
| B5HTJ5_9ACTN | Bacteria | Actinobacteria | Actinobacteria | 201174 | 3(NWH) |  |  |
| I2MXP1_9ACTN | Bacteria | Actinobacteria | Actinobacteria | 201174 | 3(NWH) |  |  |
| E2Q217_STRC2 | Bacteria | Actinobacteria | Streptomyces_  clavuligerus | 443255 | 3(NWH) |  |  |
| A0A066YW54_9ACTN | Bacteria | Actinobacteria | Actinobacteria | 201174 | 3(NWH) |  |  |
| A0A0Q8NNM3_9ACTN | Bacteria | Actinobacteria | Actinobacteria | 201174 | 3(NWH) |  |  |
| A0A0F0HPE0_9PSEU | Bacteria | Actinobacteria | Pseudonocardiales | 85010 | 3(NWH) |  |  |
| A0A0F4JZP8_9ACTN | Bacteria | Actinobacteria | Actinobacteria | 201174 | 3(NWH) |  |  |
| V6L0J0_9ACTN | Bacteria | Actinobacteria | Actinobacteria | 201174 | 3(NWH) |  |  |
| A0A1E7K201_9ACTN | Bacteria | Actinobacteria | Actinobacteria | 201174 | 3(NWH) |  |  |
| A0A0N0YQ55_9ACTN | Bacteria | Actinobacteria | Actinobacteria | 201174 | 3(NWH) |  |  |
| A0A0F7FXV5_9ACTN | Bacteria | Actinobacteria | Actinobacteria | 201174 | 3(NWH) |  |  |
| A0A0F5VPB0_9ACTN | Bacteria | Actinobacteria | Actinobacteria | 201174 | 3(NWH) |  |  |
| A0A0T6LL15_9ACTN | Bacteria | Actinobacteria | Actinobacteria | 201174 | 3(NWH) |  |  |
| A0A132MYA9_9ACTN | Bacteria | Actinobacteria | Actinobacteria | 201174 | 3(NWH) |  |  |
| Q47SB0_THEFY | Bacteria | Actinobacteria | Thermobifida_fusca | 269800 | 3(NWH) |  |  |
| D7B4D0_NOCDD | Bacteria | Actinobacteria | Nocardiopsis_  dassonvillei | 446468 | 3(NWH) |  |  |
| J7L2N7_NOCAA | Bacteria | Actinobacteria | Nocardiopsis_alba | 1205910 | 3(NWH) |  |  |
| D2SDV9_GEOOG | Bacteria | Actinobacteria | Geodermatophilus_  obscurus | 526225 | 3(NWH) |  |  |
| I4F289_9ACTN | Bacteria | Actinobacteria | Actinobacteria | 201174 | 3(NWH) |  |  |
| A0A098Y899_9ACTN | Bacteria | Actinobacteria | Actinobacteria | 201174 | 3(NWH) |  |  |
| H6RVU3_BLASD | Bacteria | Actinobacteria | Blastococcus_  saxobsidens | 1146883 | 3(NWH) |  |  |
| W5XRS5_9CORY | Bacteria | Actinobacteria | Corynebacteriaceae | 1653 | 3(NWH) |  |  |
| K9AJZ4_9MICO | Bacteria | Actinobacteria | Micrococcales | 85006 | 3(NWH) |  |  |
| A0A0B8ZWM3_BRELN | Bacteria | Actinobacteria | Brevibacterium_  linens | 1703 | 3(NWH) |  |  |
| A0A0H0ZQ18_9MICC | Bacteria | Actinobacteria | Micrococcaceae | 1268 | 3(NWH) |  |  |
| E1W0L8_GLUAR | Bacteria | Actinobacteria | Glutamicibacter_  arilaitensis | 861360 | 3(NWH) |  |  |
| M7N521_9MICC | Bacteria | Actinobacteria | Micrococcaceae | 1268 | 3(NWH) |  |  |
| A0A0M4QYZ1_9MICC | Bacteria | Actinobacteria | Micrococcaceae | 1268 | 3(NWH) |  |  |
| A0A0Q4V4V7_9MICO | Bacteria | Actinobacteria | Micrococcales | 85006 | 3(NWH) |  |  |
| A0A0Q5MVP2_9MICO | Bacteria | Actinobacteria | Micrococcales | 85006 | 3(NWH) |  |  |
| A0A0N0KTP0_9MICO | Bacteria | Actinobacteria | Micrococcales | 85006 | 3(NWH) |  |  |
| A0A0Q5L8K0_9MICO | Bacteria | Actinobacteria | Micrococcales | 85006 | 3(NWH) |  |  |
| A0A0Q5CFL3_9MICO | Bacteria | Actinobacteria | Micrococcales | 85006 | 3(NWH) |  |  |
| X5DT36_9CORY | Bacteria | Actinobacteria | Corynebacteriaceae | 1653 | 3(NWH) |  |  |
| C5CAU5_MICLC | Bacteria | Actinobacteria | Micrococcus_luteus | 465515 | 3(NWH) |  |  |
| B2GLK5_KOCRD | Bacteria | Actinobacteria | Kocuria_rhizophila | 378753 | 3(NWH) |  |  |
| A0A0P0EEI4_9MICO | Bacteria | Actinobacteria | Micrococcales | 85006 | 3(NWH) |  |  |
| A0A061LX03_9MICO | Bacteria | Actinobacteria | Micrococcales | 85006 | 3(NWH) |  |  |
| A4F6V2_SACEN | Bacteria | Actinobacteria | Saccharopolyspora_  erythraea | 405948 | 3(NWH) |  |  |
| A0A0A6UJA6_ACTUT | Bacteria | Actinobacteria | Actinoplanes_  utahensis | 1869 | 3(NWH) |  |  |
| I0GZU2_ACTM4 | Bacteria | Actinobacteria | Actinoplanes_  missouriensis | 512565 | 3(NWH) |  |  |
| A0A124G9Z0_9ACTN | Bacteria | Actinobacteria | Actinobacteria | 201174 | 3(NWH) |  |  |
| G8S1R2_ACTS5 | Bacteria | Actinobacteria | Actinoplanes_sp | 134676 | 3(NWH) |  |  |
| A0A073AWL1_9PSEU | Bacteria | Actinobacteria | Pseudonocardiales | 85010 | 3(NWH) |  |  |
| A0A1D8BQV5_9PSEU | Bacteria | Actinobacteria | Pseudonocardiales | 85010 | 3(NWH) |  |  |
| U5VVI0_9ACTN | Bacteria | Actinobacteria | Actinobacteria | 201174 | 3(NWH) |  |  |
| A0A0C1KYM8_9PSEU | Bacteria | Actinobacteria | Pseudonocardiales | 85010 | 3(NWH) |  |  |
| H5X206_9PSEU | Bacteria | Actinobacteria | Pseudonocardiales | 85010 | 3(NWH) |  |  |
| R4TIK2_AMYOR | Bacteria | Actinobacteria | Amycolatopsis_  orientalis | 31958 | 3(NWH) |  |  |
| D9V4Y4_9ACTN | Bacteria | Actinobacteria | Actinobacteria | 201174 | 3(NWH) |  |  |
| I1D750_9PSEU | Bacteria | Actinobacteria | Pseudonocardiales | 85010 | 3(NWH) |  |  |
| C7MQ74_SACVD | Bacteria | Actinobacteria | Saccharomonospora_  viridis | 471857 | 3(NWH) |  |  |
| A0A076N9D7_AMYME | Bacteria | Actinobacteria | Amycolatopsis_  methanolica | 1814 | 3(NWH) |  |  |
| G0G6K5_AMYMS | Bacteria | Actinobacteria | Amycolatopsis_  mediterranei | 713604 | 3(NWH) |  |  |
| W7IMM4_9PSEU | Bacteria | Actinobacteria | Pseudonocardiales | 85010 | 3(NWH) |  |  |
| A0A099D4K4_9ACTN | Bacteria | Actinobacteria | Actinobacteria | 201174 | 3(NWH) |  |  |
| E3J591_FRAIE | Bacteria | Actinobacteria | Frankia_inefficax | 298654 | 3(NWH) |  |  |
| Q0RQ12_FRAAA | Bacteria | Actinobacteria | Frankia_alni | 326424 | 3(NWH) |  |  |
| A0A166S207_9ACTN | Bacteria | Actinobacteria | Actinobacteria | 201174 | 3(NWH) |  |  |
| D3CVB5_9ACTN | Bacteria | Actinobacteria | Actinobacteria | 201174 | 3(NWH) |  |  |
| A0A0P0SJR7_9PSEU | Bacteria | Actinobacteria | Pseudonocardiales | 85010 | 3(NWH) |  |  |
| A0A0M4RCM5_9PSEU | Bacteria | Actinobacteria | Pseudonocardiales | 85010 | 3(NWH) |  |  |
| D6Z9W1_SEGRD | Bacteria | Actinobacteria | Segniliparus_  rotundus | 640132 | 3(NWH) |  |  |
| G7GUC8_9ACTN | Bacteria | Actinobacteria | Actinobacteria | 201174 | 3(NWH) |  |  |
| H0R0A9_9ACTN | Bacteria | Actinobacteria | Actinobacteria | 201174 | 3(NWH) |  |  |
| D0L447_GORB4 | Bacteria | Actinobacteria | Gordonia_  bronchialis | 526226 | 3(NWH) |  |  |
| H5TYZ1_9ACTN | Bacteria | Actinobacteria | Actinobacteria | 201174 | 3(NWH) |  |  |
| V8CUM0_9ACTN | Bacteria | Actinobacteria | Actinobacteria | 201174 | 3(NWH) |  |  |
| H6MUR3_GORPV | Bacteria | Actinobacteria | Gordonia_  polyisoprenivorans | 1112204 | 3(NWH) |  |  |
| K6WHQ8_9ACTN | Bacteria | Actinobacteria | Actinobacteria | 201174 | 3(NWH) |  |  |
| M0QKW1_9ACTN | Bacteria | Actinobacteria | Actinobacteria | 201174 | 3(NWH) |  |  |
| M3TMN0_9ACTN | Bacteria | Actinobacteria | Actinobacteria | 201174 | 3(NWH) |  |  |
| M7A2Z5_9ACTN | Bacteria | Actinobacteria | Actinobacteria | 201174 | 3(NWH) |  |  |
| A0A0Q5QS10_9ACTN | Bacteria | Actinobacteria | Actinobacteria | 201174 | 3(NWH) |  |  |
| U5WSR9_MYCKA | Bacteria | Actinobacteria | Mycobacterium_  kansasii | 1768 | 3(NWH) |  |  |
| X7XTP3_MYCKA | Bacteria | Actinobacteria | Mycobacterium_  kansasii | 1768 | 3(NWH) |  |  |
| B2HJ32_MYCMM | Bacteria | Actinobacteria | Mycobacterium_  marinum | 216594 | 3(NWH) |  |  |
| A0A0I9TCN2_9MYCO | Bacteria | Actinobacteria | Mycobacteriaceae | 1762 | 3(NWH) |  |  |
| A0A1A0MGE0_9MYCO | Bacteria | Actinobacteria | Mycobacteriaceae | 1762 | 3(NWH) |  |  |
| A0A1A2Z5J0_9MYCO | Bacteria | Actinobacteria | Mycobacteriaceae | 1762 | 3(NWH) |  |  |
| A0A1E3TJJ4_MYCSH | Bacteria | Actinobacteria | Mycobacterium_  shimoidei | 29313 | 3(NWH) |  |  |
| A0A1A3PGN1_9MYCO | Bacteria | Actinobacteria | Mycobacteriaceae | 1762 | 3(NWH) |  |  |
| I0RMF7_MYCXE | Bacteria | Actinobacteria | Mycobacterium_  xenopi | 1789 | 3(NWH) |  |  |
| X8C9K0_MYCXE | Bacteria | Actinobacteria | Mycobacterium_  xenopi | 1789 | 3(NWH) |  |  |
| Q743X4_MYCPA | Bacteria | Actinobacteria | Mycobacterium_  paratuberculosis | 262316 | 3(NWH) |  |  |
| X8CLW5_MYCIT | Bacteria | Actinobacteria | Mycobacterium_  intracellulare | 1767 | 3(NWH) |  |  |
| Q9CBI8_MYCLE | Bacteria | Actinobacteria | Mycobacterium_  leprae | 272631 | 3(NWH) |  |  |
| A0A1A3CA02_MYCAS | Bacteria | Actinobacteria | Mycobacterium_  asiaticum | 1790 | 3(NWH) |  |  |
| A0A024K6I8_9MYCO | Bacteria | Actinobacteria | Mycobacteriaceae | 1762 | 3(NWH) |  |  |
| A0A1E3SEN8_9MYCO | Bacteria | Actinobacteria | Mycobacteriaceae | 1762 | 3(NWH) |  |  |
| D5P8M8_9MYCO | Bacteria | Actinobacteria | Mycobacteriaceae | 1762 | 3(NWH) |  |  |
| A0A1A2ZUN1_9MYCO | Bacteria | Actinobacteria | Mycobacteriaceae | 1762 | 3(NWH) |  |  |
| A0A172UIY4_9MYCO | Bacteria | Actinobacteria | Mycobacteriaceae | 1762 | 3(NWH) |  |  |
| A0A1A3T960_9MYCO | Bacteria | Actinobacteria | Mycobacteriaceae | 1762 | 3(NWH) |  |  |
| A0A132T9S5_9MYCO | Bacteria | Actinobacteria | Mycobacteriaceae | 1762 | 3(NWH) |  |  |
| I4BQ47_MYCCN | Bacteria | Actinobacteria | Mycobacterium_  chubuense | 710421 | 3(NWH) |  |  |
| A0A1A1ZB52_9MYCO | Bacteria | Actinobacteria | Mycobacteriaceae | 1762 | 3(NWH) |  |  |
| L0J627_9MYCO | Bacteria | Actinobacteria | Mycobacteriaceae | 1762 | 3(NWH) |  |  |
| A1UMG9_MYCSK | Bacteria | Actinobacteria | Mycobacterium_sp | 189918 | 3(NWH) |  |  |
| G8RSZ0_MYCRN | Bacteria | Actinobacteria | Mycobacterium_  rhodesiae | 710685 | 3(NWH) |  |  |
| A0A1E3RSU3_9MYCO | Bacteria | Actinobacteria | Mycobacteriaceae | 1762 | 3(NWH) |  |  |
| A0A0M2ZEE0_9MYCO | Bacteria | Actinobacteria | Mycobacteriaceae | 1762 | 3(NWH) |  |  |
| W9BKA3_9MYCO | Bacteria | Actinobacteria | Mycobacteriaceae | 1762 | 3(NWH) |  |  |
| A0A132PM29_9MYCO | Bacteria | Actinobacteria | Mycobacteriaceae | 1762 | 3(NWH) |  |  |
| A0R569_MYCS2 | Bacteria | Actinobacteria | Mycobacterium_  smegmatis | 246196 | 3(NWH) |  |  |
| A0A100VXA0_9MYCO | Bacteria | Actinobacteria | Mycobacteriaceae | 1762 | 3(NWH) |  |  |
| G7CF04_MYCT3 | Bacteria | Actinobacteria | Mycobacterium_  thermoresistibile | 1078020 | 3(NWH) |  |  |
| A0A139VMN4_MYCPH | Bacteria | Actinobacteria | Mycobacterium_  phlei | 1771 | 3(NWH) |  |  |
| X5LDQ8_9MYCO | Bacteria | Actinobacteria | Mycobacteriaceae | 1762 | 3(NWH) |  |  |
| A0A0A1FY33_9MYCO | Bacteria | Actinobacteria | Mycobacteriaceae | 1762 | 3(NWH) |  |  |
| A1TG23_MYCVP | Bacteria | Actinobacteria | Mycobacterium_  vanbaalenii | 350058 | 3(NWH) |  |  |
| A0A178M2X9_9MYCO | Bacteria | Actinobacteria | Mycobacteriaceae | 1762 | 3(NWH) |  |  |
| F5YS85_MYCSD | Bacteria | Actinobacteria | Mycobacterium_  sinense | 875328 | 3(NWH) |  |  |
| A0A1B1WC23_9MYCO | Bacteria | Actinobacteria | Mycobacteriaceae | 1762 | 3(NWH) |  |  |
| A0A0D1KY19_9MYCO | Bacteria | Actinobacteria | Mycobacteriaceae | 1762 | 3(NWH) |  |  |
| A0A0T1WFM3_9MYCO | Bacteria | Actinobacteria | Mycobacteriaceae | 1762 | 3(NWH) |  |  |
| A0A0Q2R5J7_MYCGO | Bacteria | Actinobacteria | Mycobacterium_  gordonae | 1778 | 3(NWH) |  |  |
| A0A1A1XBE9_9MYCO | Bacteria | Actinobacteria | Mycobacteriaceae | 1762 | 3(NWH) |  |  |
| K5BIW2_MYCHD | Bacteria | Actinobacteria | Mycobacterium_  hassiacum | 1122247 | 3(NWH) |  |  |
| A0A0J6WCR5_9MYCO | Bacteria | Actinobacteria | Mycobacteriaceae | 1762 | 3(NWH) |  |  |
| A0A0J6WET7_9MYCO | Bacteria | Actinobacteria | Mycobacteriaceae | 1762 | 3(NWH) |  |  |
| A0A0G3IUB8_9MYCO | Bacteria | Actinobacteria | Mycobacteriaceae | 1762 | 3(NWH) |  |  |
| X8E0Y9_9MYCO | Bacteria | Actinobacteria | Mycobacteriaceae | 1762 | 3(NWH) |  |  |
| E9T1H5_RHOHA | Bacteria | Actinobacteria | Rhodococcus_  hoagii | 43767 | 3(NWH) |  |  |
| A0A177YK21_9NOCA | Bacteria | Actinobacteria | Nocardiaceae | 85025 | 3(NWH) |  |  |
| A0A143QD21_9NOCA | Bacteria | Actinobacteria | Nocardiaceae | 85025 | 3(NWH) |  |  |
| A0A164KY00_9NOCA | Bacteria | Actinobacteria | Nocardiaceae | 85025 | 3(NWH) |  |  |
| A0A0D0LDR0_9NOCA | Bacteria | Actinobacteria | Nocardiaceae | 85025 | 3(NWH) |  |  |
| A0A161X6Q5_9NOCA | Bacteria | Actinobacteria | Nocardiaceae | 85025 | 3(NWH) |  |  |
| A0A1A2BIL8_9MYCO | Bacteria | Actinobacteria | Mycobacteriaceae | 1762 | 3(NWH) |  |  |
| W5T8G0_9NOCA | Bacteria | Actinobacteria | Nocardiaceae | 85025 | 3(NWH) |  |  |
| A0A0B8N9M9_9NOCA | Bacteria | Actinobacteria | Nocardiaceae | 85025 | 3(NWH) |  |  |
| K0ERV2_9NOCA | Bacteria | Actinobacteria | Nocardiaceae | 85025 | 3(NWH) |  |  |
| H6RB15_NOCCG | Bacteria | Actinobacteria | Nocardia_  cyriacigeorgica | 1127134 | 3(NWH) |  |  |
| Q0S8B4_RHOJR | Bacteria | Actinobacteria | Rhodococcus_jostii | 101510 | 3(NWH) |  |  |
| C0ZPP4_RHOE4 | Bacteria | Actinobacteria | Rhodococcus_  erythropolis | 234621 | 3(NWH) |  |  |
| A0A059MU50_9NOCA | Bacteria | Actinobacteria | Nocardiaceae | 85025 | 3(NWH) |  |  |
| Q5Z2S2_NOCFA | Bacteria | Actinobacteria | Nocardia_farcinica | 247156 | 3(NWH) |  |  |
| V9XGE0_9NOCA | Bacteria | Actinobacteria | Nocardiaceae | 85025 | 3(NWH) |  |  |
| A0A0K2YHJ0_9NOCA | Bacteria | Actinobacteria | Nocardiaceae | 85025 | 3(NWH) |  |  |
| R7WML8_9NOCA | Bacteria | Actinobacteria | Nocardiaceae | 85025 | 3(NWH) |  |  |
| U5EBD1_NOCAS | Bacteria | Actinobacteria | Nocardia_  asteroides | 1824 | 3(NWH) |  |  |
| A0A137ZYV7_9ACTN | Bacteria | Actinobacteria | Actinobacteria | 201174 | 3(NWH) |  |  |
| F6EGX9_HOYSD | Bacteria | Actinobacteria | Hoyosella_subflava | 443218 | 3(NWH) |  |  |
| G7H1I7_9ACTN | Bacteria | Actinobacteria | Actinobacteria | 201174 | 3(NWH) |  |  |
| M3VAS8_9ACTN | Bacteria | Actinobacteria | Actinobacteria | 201174 | 3(NWH) |  |  |
| L7LGA0_9ACTN | Bacteria | Actinobacteria | Actinobacteria | 201174 | 3(NWH) |  |  |
| A0A0N9N7Q2_9ACTN | Bacteria | Actinobacteria | Actinobacteria | 201174 | 3(NWH) |  |  |
| L7LCV8_9ACTN | Bacteria | Actinobacteria | Actinobacteria | 201174 | 3(NWH) |  |  |
| A0A0Q7B861_9ACTN | Bacteria | Actinobacteria | Actinobacteria | 201174 | 3(NWH) |  |  |
| A0A1A9GRK8_9ACTN | Bacteria | Actinobacteria | Actinobacteria | 201174 | 3(NWH) |  |  |
| A0A0Q9RZY9_9ACTN | Bacteria | Actinobacteria | Actinobacteria | 201174 | 3(NWH) |  |  |
| A0A0Q7YYH5_9ACTN | Bacteria | Actinobacteria | Actinobacteria | 201174 | 3(NWH) |  |  |
| E6J7G2_9ACTN | Bacteria | Actinobacteria | Actinobacteria | 201174 | 3(NWH) |  |  |
| A0A1E3RTM1_9ACTN | Bacteria | Actinobacteria | Actinobacteria | 201174 | 3(NWH) |  |  |
| A0A1J4N8D6_9ACTN | Bacteria | Actinobacteria | Actinobacteria | 201174 | 3(NWH) |  |  |
| A0A0Q8VHZ5_9ACTN | Bacteria | Actinobacteria | Actinobacteria | 201174 | 3(NWH) |  |  |
| A0A0A1DR65_NOCSI | Bacteria | Actinobacteria | Nocardioides_  simplex | 2045 | 3(NWH) |  |  |
| A0A0Q8PZE0_9ACTN | Bacteria | Actinobacteria | Actinobacteria | 201174 | 3(NWH) |  |  |
| A0A1A3JCL4_9MYCO | Bacteria | Actinobacteria | Mycobacteriaceae | 1762 | 3(NWH) |  |  |
| A0A1E3SVF4_9MYCO | Bacteria | Actinobacteria | Mycobacteriaceae | 1762 | 3(NWH) |  |  |
| K0JQ72_SACES | Bacteria | Actinobacteria | Saccharothrix_  espanaensis | 1179773 | 3(NWH) |  |  |
| A0A0M8YK53_9PSEU | Bacteria | Actinobacteria | Pseudonocardiales | 85010 | 3(NWH) |  |  |
| C6WGJ2_ACTMD | Bacteria | Actinobacteria | Actinosynnema_  mirum | 446462 | 3(NWH) |  |  |
| A0A0F0GM07_NOCAE | Bacteria | Actinobacteria | Lechevalieria_  aerocolonigenes | 68170 | 3(NWH) |  |  |
| A0A0M8W999_9NOCA | Bacteria | Actinobacteria | Nocardiaceae | 85025 | 3(NWH) |  |  |
| D5UST7_TSUPD | Bacteria | Actinobacteria | Tsukamurella_  paurometabola | 521096 | 3(NWH) |  |  |
| D9T4K5_MICAI | Bacteria | Actinobacteria | Micromonospora_  aurantiaca | 644283 | 3(NWH) |  |  |
| F4F197_VERMA | Bacteria | Actinobacteria | Verrucosispora_  maris | 263358 | 3(NWH) |  |  |
| F4CLT0_PSEUX | Bacteria | Actinobacteria | Pseudonocardia_  dioxanivorans | 675635 | 3(NWH) |  |  |
| A0A1E4P0F2_9PSEU | Bacteria | Actinobacteria | Pseudonocardiales | 85010 | 3(NWH) |  |  |
| D9QC27_CORP2 | Bacteria | Actinobacteria | Corynebacterium_  pseudotuberculosis | 681645 | 3(NWH) |  |  |
| A0A0B6TK15_9CORY | Bacteria | Actinobacteria | Corynebacteriaceae | 1653 | 3(NWH) |  |  |
| A0A0G3H625_9CORY | Bacteria | Actinobacteria | Corynebacteriaceae | 1653 | 3(NWH) |  |  |
| M1NWI6_9CORY | Bacteria | Actinobacteria | Corynebacteriaceae | 1653 | 3(NWH) |  |  |
| A0A096AGB5_9CORY | Bacteria | Actinobacteria | Corynebacteriaceae | 1653 | 3(NWH) |  |  |
| S5THU3_9CORY | Bacteria | Actinobacteria | Corynebacteriaceae | 1653 | 3(NWH) |  |  |
| A0A076NMS4_9CORY | Bacteria | Actinobacteria | Corynebacteriaceae | 1653 | 3(NWH) |  |  |
| A0A077HJN0_9CORY | Bacteria | Actinobacteria | Corynebacteriaceae | 1653 | 3(NWH) |  |  |
| A0A0G3HBB7_9CORY | Bacteria | Actinobacteria | Corynebacteriaceae | 1653 | 3(NWH) |  |  |
| C0XQ83_9CORY | Bacteria | Actinobacteria | Corynebacteriaceae | 1653 | 3(NWH) |  |  |
| D7WEQ1_9CORY | Bacteria | Actinobacteria | Corynebacteriaceae | 1653 | 3(NWH) |  |  |
| A0A097IEH8_9CORY | Bacteria | Actinobacteria | Corynebacteriaceae | 1653 | 3(NWH) |  |  |
| A0A0K1RER0_9CORY | Bacteria | Actinobacteria | Corynebacteriaceae | 1653 | 3(NWH) |  |  |
| Q8FLN9_COREF | Bacteria | Actinobacteria | Corynebacterium_  efficiens | 196164 | 3(NWH) |  |  |
| Q8NLF1_CORGL | Bacteria | Actinobacteria | Corynebacterium_  glutamicum | 196627 | 3(NWH) |  |  |
| K1E2A9_9MICO | Bacteria | Actinobacteria | Micrococcales | 85006 | 3(NWH) |  |  |
| A0A0Q9K7T9_9MICO | Bacteria | Actinobacteria | Micrococcales | 85006 | 3(NWH) |  |  |
| Q6NFP6_CORDI | Bacteria | Actinobacteria | Corynebacterium_  diphtheriae | 257309 | 3(NWH) |  |  |
| L1MC46_9CORY | Bacteria | Actinobacteria | Corynebacteriaceae | 1653 | 3(NWH) |  |  |
| C7QYN2_JONDD | Bacteria | Actinobacteria | Jonesia_  denitrificans | 471856 | 3(NWH) |  |  |
| A0A0S9Q485_9ACTN | Bacteria | Actinobacteria | Actinobacteria | 201174 | 3(NWH) |  |  |
| A0A0N9HV33_9PSEU | Bacteria | Actinobacteria | Pseudonocardiales | 85010 | 3(NWH) |  |  |
| A0A0Q0UFT1_9CORY | Bacteria | Actinobacteria | Corynebacteriaceae | 1653 | 3(NWH) |  |  |
| A0A172X5D3_9MICO | Bacteria | Actinobacteria | Micrococcales | 85006 | 3(NWH) |  |  |
| A0A175RF64_9MICO | Bacteria | Actinobacteria | Micrococcales | 85006 | 3(NWH) |  |  |
| M2YBQ4_9MICC | Bacteria | Actinobacteria | Micrococcaceae | 1268 | 3(NWH) |  |  |
| D4YL80_9MICO | Bacteria | Actinobacteria | Micrococcales | 85006 | 3(NWH) |  |  |
| A0A0G3H6E2_9CORY | Bacteria | Actinobacteria | Corynebacteriaceae | 1653 | 3(NWH) |  |  |
| W5WP97_9CORY | Bacteria | Actinobacteria | Corynebacteriaceae | 1653 | 3(NWH) |  |  |
| A0A1B7LXS4_9MICC | Bacteria | Actinobacteria | Micrococcaceae | 1268 | 3(NWH) |  |  |
| I7IXM5_9CORY | Bacteria | Actinobacteria | Corynebacteriaceae | 1653 | 3(NWH) |  |  |
| A0A088QGL6_9CORY | Bacteria | Actinobacteria | Corynebacteriaceae | 1653 | 3(NWH) |  |  |
| W9GLG3_9MICO | Bacteria | Actinobacteria | Micrococcales | 85006 | 3(NWH) |  |  |
| W7T2I9_9PSEU | Bacteria | Actinobacteria | Pseudonocardiales | 85010 | 3(NWH) |  |  |
| W5WCM7_9PSEU | Bacteria | Actinobacteria | Pseudonocardiales | 85010 | 3(NWH) |  |  |
| M5AGN8_9ACTN | Bacteria | Actinobacteria | Actinobacteria | 201174 | 3(NWH) |  |  |
| H0EBN1_9ACTN | Bacteria | Actinobacteria | Actinobacteria | 201174 | 3(NWH) |  |  |
| E0DGK3_9CORY | Bacteria | Actinobacteria | Corynebacteriaceae | 1653 | 3(NWH) |  |  |
| A0A075TX84_9CORY | Bacteria | Actinobacteria | Corynebacteriaceae | 1653 | 3(NWH) |  |  |
| E3H3C9_ROTDC | Bacteria | Actinobacteria | Rothia_  dentocariosa | 762948 | 3(NWH) |  |  |
| A4X1G9_SALTO | Bacteria | Actinobacteria | Salinispora_  tropica | 369723 | 3(NWH) |  |  |
| A0A0F0GYW8_NOCAE | Bacteria | Actinobacteria | Lechevalieria_  aerocolonigenes | 68170 | 3(NWH) |  |  |
| A0A0N0A1A8_9NOCA | Bacteria | Actinobacteria | Nocardiaceae | 85025 | 3(NWH) |  |  |
| Q1AWN0_RUBXD | Bacteria | Actinobacteria | Rubrobacter_  xylanophilus | 266117 | na |  | failed iqtree; removed from phylogenetic analysis |
| A0A0Q9TGA2_9ACTN | Bacteria | Actinobacteria | Actinobacteria | 201174 | na |  | failed iqtree; removed from phylogenetic analysis |
| A0A0S9R9W1_9ACTN | Bacteria | Actinobacteria | Actinobacteria | 201174 | na |  | failed iqtree; removed from phylogenetic analysis |
| A0A0B2B830_9ACTN | Bacteria | Actinobacteria | Actinobacteria | 201174 | na |  | failed iqtree; removed from phylogenetic analysis |
| A0A0A0C0N4_9CELL | Bacteria | Actinobacteria | Cellulomonadaceae | 85016 | na |  | failed iqtree; removed from phylogenetic analysis |
| A0A021VYC4_9CELL | Bacteria | Actinobacteria | Cellulomonadaceae | 85016 | na |  | failed iqtree; removed from phylogenetic analysis |
| C7NJ91_KYTSD | Bacteria | Actinobacteria | Kytococcus_  sedentarius | 478801 | na |  | failed iqtree; removed from phylogenetic analysis |
| R4YWA3_9ACTN | Bacteria | Actinobacteria | Actinobacteria | 201174 | na |  | failed iqtree; removed from phylogenetic analysis |
| K6X673_9MICO | Bacteria | Actinobacteria | Micrococcales | 85006 | na |  | failed iqtree; removed from phylogenetic analysis |
| H5USM1_9MICO | Bacteria | Actinobacteria | Micrococcales | 85006 | na |  | failed iqtree; removed from phylogenetic analysis |
| A0A127A6J9_9MICC | Bacteria | Actinobacteria | Micrococcaceae | 1268 | na |  | failed iqtree; removed from phylogenetic analysis |
| A0A0B2AQ08_9MICC | Bacteria | Actinobacteria | Micrococcaceae | 1268 | na |  | failed iqtree; removed from phylogenetic analysis |
| D2AVP2_STRRD | Bacteria | Actinobacteria | Streptosporangium_roseum | 479432 | na |  | failed iqtree; removed from phylogenetic analysis |
| A0A161LNP0_9ACTN | Bacteria | Actinobacteria | Actinobacteria | 201174 | na |  | failed iqtree; removed from phylogenetic analysis |
| W2EJN4_9ACTN | Bacteria | Actinobacteria | Actinobacteria | 201174 | na |  | failed iqtree; removed from phylogenetic analysis |
| B4V3Z5_9ACTN | Bacteria | Actinobacteria | Actinobacteria | 201174 | na |  | failed iqtree; removed from phylogenetic analysis |
| A0A0N0N657_9ACTN | Bacteria | Actinobacteria | Actinobacteria | 201174 | na |  | failed iqtree; removed from phylogenetic analysis |
| A0A0X3S049_9ACTN | Bacteria | Actinobacteria | Actinobacteria | 201174 | na |  | failed iqtree; removed from phylogenetic analysis |
| Q2J6D1_FRACC | Bacteria | Actinobacteria | Frankia_casuarinae | 106370 | na |  | failed iqtree; removed from phylogenetic analysis |
| Q0RCY6_FRAAA | Bacteria | Actinobacteria | Frankia_alni | 326424 | na |  | failed iqtree; removed from phylogenetic analysis |
| A0A166Q9K2_9ACTN | Bacteria | Actinobacteria | Actinobacteria | 201174 | na |  | failed iqtree; removed from phylogenetic analysis |
| D3D6D5_9ACTN | Bacteria | Actinobacteria | Actinobacteria | 201174 | na |  | failed iqtree; removed from phylogenetic analysis |
| A8L2H5_FRASN | Bacteria | Actinobacteria | Frankia_sp | 298653 | na |  | failed iqtree; removed from phylogenetic analysis |
| F8AX51_FRADG | Bacteria | Actinobacteria | Frankia_  symbiont subsp | 656024 | na |  | failed iqtree; removed from phylogenetic analysis |
| A0A126Z9B6_9MICO | Bacteria | Actinobacteria | Micrococcales | 85006 | na |  | failed iqtree; removed from phylogenetic analysis |
| A0A0H5CT56_9PSEU | Bacteria | Actinobacteria | Pseudonocardiales | 85010 | na |  | failed iqtree; removed from phylogenetic analysis |
| A6W7P6_KINRD | Bacteria | Actinobacteria | Kineococcus_  radiotolerans | 266940 | na |  | failed iqtree; removed from phylogenetic analysis |
| A0A0Q8E4B7_9ACTN | Bacteria | Actinobacteria | Actinobacteria | 201174 | na |  | failed iqtree; removed from phylogenetic analysis |
| A0A125Q117_9ACTN | Bacteria | Actinobacteria | Actinobacteria | 201174 | na |  | failed iqtree; removed from phylogenetic analysis |
| A0A0D0VZH7_9ACTN | Bacteria | Actinobacteria | Actinobacteria | 201174 | na |  | failed iqtree; removed from phylogenetic analysis |
| C4RGJ4_9ACTN | Bacteria | Actinobacteria | Actinobacteria | 201174 | na |  | failed iqtree; removed from phylogenetic analysis |
| A0A0M2RLR7_9ACTN | Bacteria | Actinobacteria | Actinobacteria | 201174 | na |  | failed iqtree; removed from phylogenetic analysis |
| I0L0M5_9ACTN | Bacteria | Actinobacteria | Actinobacteria | 201174 | na |  | failed iqtree; removed from phylogenetic analysis |
| A0A136PPU2_9ACTN | Bacteria | Actinobacteria | Actinobacteria | 201174 | na |  | failed iqtree; removed from phylogenetic analysis |
| D1BDF9_SANKS | Bacteria | Actinobacteria | Sanguibacter_keddieii | 446469 | na |  | failed iqtree; removed from phylogenetic analysis |
| A0A0J0V0W2_9ACTN | Bacteria | Actinobacteria | Actinobacteria | 201174 | na |  | failed iqtree; removed from phylogenetic analysis |
| A0A0Q6FGB3_9ACTN | Bacteria | Actinobacteria | Actinobacteria | 201174 | na |  | failed iqtree; removed from phylogenetic analysis |
| A0A0Q7JLE7_9ACTN | Bacteria | Actinobacteria | Actinobacteria | 201174 | na |  | failed iqtree; removed from phylogenetic analysis |
| A0A101SMY0_9ACTN | Bacteria | Actinobacteria | Actinobacteria | 201174 | na |  | failed iqtree; removed from phylogenetic analysis |
| A0A0U3NJK9_9ACTN | Bacteria | Actinobacteria | Actinobacteria | 201174 | na |  | failed iqtree; removed from phylogenetic analysis |
| O54100_STRCO | Bacteria | Actinobacteria | Streptomyces_  coelicolor | 100226 | na |  | failed iqtree; removed from phylogenetic analysis |
| A0A0M8WFP2_9NOCA | Bacteria | Actinobacteria | Nocardiaceae | 85025 | na |  | failed iqtree; removed from phylogenetic analysis |
| A0A0Q4HC32_9MICO | Bacteria | Actinobacteria | Micrococcales | 85006 | na |  | failed iqtree; removed from phylogenetic analysis |
| A0A0X8E3N2_9MICO | Bacteria | Actinobacteria | Micrococcales | 85006 | na |  | failed iqtree; removed from phylogenetic analysis |
| A0A089XKJ5_STRGA | Bacteria | Actinobacteria | Streptomyces_  glaucescens | 1907 | na |  | failed iqtree; removed from phylogenetic analysis |
| D2AY66_STRRD | Bacteria | Actinobacteria | Streptosporangium_  roseum | 479432 | na |  | failed iqtree; removed from phylogenetic analysis |
| A0A0K9XA78_9ACTN | Bacteria | Actinobacteria | Actinobacteria | 201174 | na |  | failed iqtree; removed from phylogenetic analysis |
| D2AY38_STRRD | Bacteria | Actinobacteria | Streptosporangium_  roseum | 479432 | na |  | failed iqtree; removed from phylogenetic analysis |
| D2AY67_STRRD | Bacteria | Actinobacteria | Streptosporangium_  roseum | 479432 | na |  | failed iqtree; removed from phylogenetic analysis |
| W5T828_9NOCA | Bacteria | Actinobacteria | Nocardiaceae | 85025 | na |  | failed iqtree; removed from phylogenetic analysis |
| A0A0K9XL25_9ACTN | Bacteria | Actinobacteria | Actinobacteria | 201174 | na |  | failed iqtree; removed from phylogenetic analysis |
| U5EK13_NOCAS | Bacteria | Actinobacteria | Nocardia_  asteroides | 1824 | na |  | failed iqtree; removed from phylogenetic analysis |
| A0A1E5P2Y9_9ACTN | Bacteria | Actinobacteria | Actinobacteria | 201174 | na |  | failed iqtree; removed from phylogenetic analysis |
| G2G7I6_9ACTN | Bacteria | Actinobacteria | Actinobacteria | 201174 | na |  | failed iqtree; removed from phylogenetic analysis |
| A8L408_FRASN | Bacteria | Actinobacteria | Frankia_sp | 298653 | na |  | failed iqtree; removed from phylogenetic analysis |
| C9ZEK2_STRSW | Bacteria | Actinobacteria | Streptomyces_  scabiei | 680198 | na |  | failed iqtree; removed from phylogenetic analysis |
| A0A1A0TF16_9MYCO | Bacteria | Actinobacteria | Mycobacteriaceae | 1762 | na |  | failed iqtree; removed from phylogenetic analysis |
| D3EZA8_CONWI | Bacteria | Actinobacteria | Conexibacter_  woesei | 469383 | na |  | long branch; removed from final phylogenetic analysis |
| C4LLL6_CORK4 | Bacteria | Actinobacteria | Corynebacterium_  kroppenstedtii | 645127 | na |  | long branch; removed from final phylogenetic analysis |
| K0YNF4_9ACTO | Bacteria | Actinobacteria | Actinomycetales | 2037 | na |  | long branch; removed from final phylogenetic analysis |
| A0A0F0GP16_NOCAE | Bacteria | Actinobacteria | Lechevalieria_  aerocolonigenes | 68170 | na |  | long branch; removed from final phylogenetic analysis |
| A0A0Q9J7K0_9MICO | Bacteria | Actinobacteria | Micrococcales | 85006 | na |  | long branch; removed from final phylogenetic analysis |
| A0A0Q8CKB6_9MICO | Bacteria | Actinobacteria | Micrococcales | 85006 | na |  | long branch; removed from final phylogenetic analysis |
| A0A1E8F4W7_9MICC | Bacteria | Actinobacteria | Micrococcaceae | 1268 | na |  | long branch; removed from final phylogenetic analysis |
| W7T0M6_9PSEU | Bacteria | Actinobacteria | Pseudonocardiales | 85010 | na |  | long branch; removed from final phylogenetic analysis |
| F8B0H0_FRADG | Bacteria | Actinobacteria | Frankia_  symbiont subsp | 656024 | na |  | long branch; removed from final phylogenetic analysis |
| E3IZH0_FRAIE | Bacteria | Actinobacteria | Frankia_inefficax | 298654 | na |  | long branch; removed from final phylogenetic analysis |
| L7F1N0_9ACTN | Bacteria | Actinobacteria | Actinobacteria | 201174 | na |  | long branch; removed from final phylogenetic analysis |
| A0A124G6X0_9ACTN | Bacteria | Actinobacteria | Actinobacteria | 201174 | na |  | long branch; removed from final phylogenetic analysis |
| A0A0M8WNX2_9ACTN | Bacteria | Actinobacteria | Actinobacteria | 201174 | na |  | long branch; removed from final phylogenetic analysis |
| A0A0N0HZV1_9ACTN | Bacteria | Actinobacteria | Actinobacteria | 201174 | na |  | long branch; removed from final phylogenetic analysis |
| G0FY96_AMYMS | Bacteria | Actinobacteria | Amycolatopsis_  mediterranei | 713604 | na |  | long branch; removed from final phylogenetic analysis |
| R4SW22_AMYOR | Bacteria | Actinobacteria | Amycolatopsis_  orientalis | 31958 | na |  | long branch; removed from final phylogenetic analysis |
| B1VTB6_STRGG | Bacteria | Actinobacteria | Streptomyces_  griseus subsp | 455632 | na |  | long branch; removed from final phylogenetic analysis |
| V6UH64_9ACTN | Bacteria | Actinobacteria | Actinobacteria | 201174 | na |  | long branch; removed from final phylogenetic analysis |
| I2N5E4_9ACTN | Bacteria | Actinobacteria | Actinobacteria | 201174 | na |  | long branch; removed from final phylogenetic analysis |
| G2GGS3_9ACTN | Bacteria | Actinobacteria | Actinobacteria | 201174 | na |  | long branch; removed from final phylogenetic analysis |
| A0A197SI33_9ACTN | Bacteria | Actinobacteria | Actinobacteria | 201174 | na |  | long branch; removed from final phylogenetic analysis |
| A0A0A6US84_ACTUT | Bacteria | Actinobacteria | Actinoplanes_  utahensis | 1869 | na |  | long branch; removed from final phylogenetic analysis |
| I0L7D6_9ACTN | Bacteria | Actinobacteria | Actinobacteria | 201174 | na |  | long branch; removed from final phylogenetic analysis |
| A0A0M8YDH2_9ACTN | Bacteria | Actinobacteria | Actinobacteria | 201174 | na |  | long branch; removed from final phylogenetic analysis |
| C1DU99_SULAA | Bacteria | Aquificae | Sulfurihydrogenibium_azorense | 204536 | 1(TYH) |  |  |
| B2V725_SULSY | Bacteria | Aquificae | Sulfurihydrogenibium_sp | 436114 | na |  | failed iqtree; removed from phylogenetic analysis |
| B8LE39_THAPS | Eukaryote | Bacillariophyta | Thalassiosira_  pseudonana | 35128 | 3(NWH) |  | N terminal ABM domain |
| K0TJM3_THAOC | Eukaryote | Bacillariophyta | Thalassiosira_  oceanica | 159749 | 3(NWH) |  | N terminal ABM domain |
| K0TJM3_THAOC | Eukaryote | Bacillariophyta | Thalassiosira_  oceanica | 159749 | 3(NWH) |  | C terminal ABM domain |
| B8LE39_THAPS | Eukaryote | Bacillariophyta | Thalassiosira_  pseudonana | 35128 | 3(NWH) |  | C terminal ABM domain |
| A0A1E7F1Z8_9STRA | Eukaryote | Bacillariophyta | Fragilariopsis_  cylindrus | 635003 | 3(NWH) |  | N terminal ABM domain |
| A0A1E7F1Z8_9STRA | Eukaryote | Bacillariophyta | Fragilariopsis_  cylindrus | 635003 | 3(NWH) |  | C terminal ABM domain |
| A0A098C3W9_9PORP | Bacteria | Bacteroidetes  Chlorobi | Porphyromonadaceae | 171551 | na |  | long branch; removed from final phylogenetic analysis |
| D6THY3_9CHLR | Bacteria | Chloroflexi | Chloroflexi | 200795 | 2(NYH) |  |  |
| A0A0M9UCJ3_9CHLR | Bacteria | Chloroflexi | Chloroflexi | 200795 | 3(NWH) |  |  |
| A9WHC7_CHLAA | Bacteria | Chloroflexi | Chloroflexus_  aurantiacus | 324602 | 3(NWH) |  |  |
| A5UZ74_ROSS1 | Bacteria | Chloroflexi | Roseiflexus_sp | 357808 | 3(NWH) |  |  |
| A0A136LHU6_9CHLR | Bacteria | Chloroflexi | Chloroflexi | 200795 | 3(NWH) |  |  |
| A8I5W8_CHLRE | Eukaryote | Chlorophyta | Chlamydomonas_  reinhardtii | 3055 | 3(NWH) | LFO1 | failed iqtree; but retained eukaryotic sequence |
| A0A090N3X1_OSTTA | Eukaryote | Chlorophyta | Ostreococcus_tauri | 70448 | 2(NWR) |  | C terminal ABM domain |
| A4S0P7_OSTLU | Eukaryote | Chlorophyta | Ostreococcus_  lucimarinus | 436017 | 2(NWR) |  | C terminal ABM domain |
| C1MUT0_MICPC | Eukaryote | Chlorophyta | Micromonas_pusilla | 564608 | 3(NWH) |  | C terminal ABM domain |
| D8U6C7_VOLCA | Eukaryote | Chlorophyta | Volvox_carteri | 3067 | 3(NWH) |  |  |
| C1E6M3_MICCC | Eukaryote | Chlorophyta | Micromonas_  commoda | 296587 | 3(NWH) |  | N terminal ABM domain |
| C1MUT0_MICPC | Eukaryote | Chlorophyta | Micromonas_pusilla | 564608 | 3(NWH) |  | N terminal ABM domain |
| A0A090N3X1_OSTTA | Eukaryote | Chlorophyta | Ostreococcus_tauri | 70448 | 3(NWH) |  | N terminal ABM domain |
| A4S0P7_OSTLU | Eukaryote | Chlorophyta | Ostreococcus_  lucimarinus | 436017 | 3(NWH) |  | N terminal ABM domain |
| C1E6M3_MICCC | Eukaryote | Chlorophyta | Micromonas_  commoda | 296587 | 3(NWH) |  | C terminal ABM domain |
| Q9YCC3_AERPE | Archaea | Crenarchaeota | Aeropyrum_pernix | 272557 | 3(NWH) |  | failed iqtree; but retained archaeal sequence |
| A0A166TTZ3_9CYAN | Bacteria | Cyanobacteria | Cyanobacteria | 1117 | 3(NWH) |  |  |
| A0A0C1VFH2_9CYAN | Bacteria | Cyanobacteria | Cyanobacteria | 1117 | 3(NWH) |  |  |
| B0C735_ACAM1 | Bacteria | Cyanobacteria | Acaryochloris_  marina | 329726 | na |  | failed iqtree; removed from phylogenetic analysis |
| A0A0C1Y524_9CYAN | Bacteria | Cyanobacteria | Cyanobacteria | 1117 | na |  | long branch; removed from final phylogenetic analysis |
| A0A0C2M485_9CYAN | Bacteria | Cyanobacteria | Cyanobacteria | 1117 | na |  | long branch; removed from final phylogenetic analysis |
| A0A0C2QMC1_9CYAN | Bacteria | Cyanobacteria | Cyanobacteria | 1117 | na |  | long branch; removed from final phylogenetic analysis |
| L0A398_DEIPD | Bacteria | Deinococcus  Thermus | Deinococcus_  peraridilitoris | 937777 | 3(NWH) |  |  |
| H8GT32_DEIGI | Bacteria | Deinococcus  Thermus | Deinococcus_  gobiensis | 745776 | 3(NWH) |  |  |
| A0A0Q1AZ35_9DEIO | Bacteria | Deinococcus  Thermus | Deinococcaceae | 183710 | 3(NWH) |  |  |
| A0A0F7JTH1_9DEIO | Bacteria | Deinococcus  Thermus | Deinococcaceae | 183710 | 3(NWH) |  |  |
| Q1IZZ8_DEIGD | Bacteria | Deinococcus  Thermus | Deinococcus_  geothermalis | 319795 | 3(NWH) |  |  |
| F0RN07_DEIPM | Bacteria | Deinococcus  Thermus | Deinococcus_  proteolyticus | 693977 | 3(NWH) |  |  |
| A0A0A7KGV0_9DEIO | Bacteria | Deinococcus  Thermus | Deinococcaceae | 183710 | 3(NWH) |  |  |
| Q1IW94_DEIGD | Bacteria | Deinococcus  Thermus | Deinococcus_  geothermalis | 319795 | 3(NWH) |  |  |
| E8U471_DEIML | Bacteria | Deinococcus  Thermus | Deinococcus_  maricopensis | 709986 | 3(NWH) |  |  |
| D7CVU1_TRURR | Bacteria | Deinococcus  Thermus | Truepera_  radiovictrix | 649638 | 3(NWH) |  |  |
| Q53VV7_THET8 | Bacteria | Deinococcus  Thermus | Thermus_  thermophilus | 300852 | 3(NWH) |  |  |
| D3PM89_MEIRD | Bacteria | Deinococcus  Thermus | Meiothermus_ruber | 504728 | 3(NWH) |  |  |
| D7BHV0_MEISD | Bacteria | Deinococcus  Thermus | Meiothermus_  silvanus | 526227 | 3(NWH) |  |  |
| E4U5B9_OCEP5 | Bacteria | Deinococcus  Thermus | Oceanithermus_  profundus | 670487 | 3(NWH) |  |  |
| F2NKW3_MARHT | Bacteria | Deinococcus  Thermus | Marinithermus_  hydrothermalis | 869210 | 3(NWH) |  |  |
| Q9RX69_DEIRA | Bacteria | Deinococcus  Thermus | Deinococcus_  radiodurans | 243230 | 3(NWH) |  |  |
| F0RKB4_DEIPM | Bacteria | Deinococcus  Thermus | Deinococcus_  proteolyticus | 693977 | 3(NWH) |  |  |
| Q9RXN8_DEIRA | Bacteria | Deinococcus  Thermus | Deinococcus_  radiodurans | 243230 | 3(NWH) |  |  |
| A0A0F7JLG3_9DEIO | Bacteria | Deinococcus  Thermus | Deinococcaceae | 183710 | na |  | failed iqtree; removed from phylogenetic analysis |
| T0LQ28_9EURY | Archaea | Euryarchaeota | Euryarchaeota | 28890 | 3(NWH) |  |  |
| A0A166TA40_9EURY | Archaea | Euryarchaeota | Euryarchaeota | 28890 | 3(NWH) |  |  |
| T0N5V9_9EURY | Archaea | Euryarchaeota | Euryarchaeota | 28890 | 3(NWH) |  |  |
| A0A1M4Q4X2_STAAU | Bacteria | Firmicutes | Staphylococcus_  aureus | 1280 | 3(NWH) | IsdG |  |
| A0A133QBY0_STALU | Bacteria | Firmicutes | Staphylococcus_  lugdunensis | 28035 | 3(NWH) | IsdG |  |
| HDOX_BACAN | Bacteria | Firmicutes | Bacillus_anthracis | 1392 | 3(NWH) | IsdG |  |
| HDOX_LISMO | Bacteria | Firmicutes | Listeria_  monocytogenes serovar 1 | 169963 | 3(NWH) | IsdG |  |
| A0A113JIK9_STAAU | Bacteria | Firmicutes | Staphylococcus_  aureus | 1280 | 3(NWH) | IsdI |  |
| A8FBM3_BACP2 | Bacteria | Firmicutes | Bacillus_pumilus | 315750 | 1(NFN) |  |  |
| A0A0A3IHC6_9BACI | Bacteria | Firmicutes | Bacillaceae | 186817 | 1(QWL) |  |  |
| F5SD85_9BACL | Bacteria | Firmicutes | Bacillales | 1385 | 2(-WH) |  |  |
| A0A1B9AQP0_9BACI | Bacteria | Firmicutes | Bacillaceae | 186817 | 2(-WH) |  |  |
| X0R6N6_9BACI | Bacteria | Firmicutes | Bacillaceae | 186817 | 2(KWH) |  |  |
| A0A094YSI1_BACAO | Bacteria | Firmicutes | Bacillus_  alcalophilus | 1445 | 2(KWH) |  |  |
| A0A081PAW1_9BACL | Bacteria | Firmicutes | Bacillales | 1385 | 2(KWH) |  |  |
| H3SCR7_9BACL | Bacteria | Firmicutes | Bacillales | 1385 | 2(KWH) |  |  |
| C3BLJ9_9BACI | Bacteria | Firmicutes | Bacillaceae | 186817 | 2(KWH) |  |  |
| Q81P32_BACAN | Bacteria | Firmicutes | Bacillus_anthracis | 1392 | 2(KWH) |  |  |
| Q3EP94_BACTI | Bacteria | Firmicutes | Bacillus_  thuringiensis subsp | 1430 | 2(KWH) |  |  |
| Q81C15_BACCR | Bacteria | Firmicutes | Bacillus_cereus | 226900 | 2(KWH) |  |  |
| A0A073JX78_9BACI | Bacteria | Firmicutes | Bacillaceae | 186817 | 2(KWH) |  |  |
| M8DI09_9BACL | Bacteria | Firmicutes | Bacillales | 1385 | 2(KWH) |  |  |
| V9W098_9BACL | Bacteria | Firmicutes | Bacillales | 1385 | 2(KWH) |  |  |
| A0A0M2VWE2_9BACL | Bacteria | Firmicutes | Bacillales | 1385 | 2(KWH) |  |  |
| D3E9W0_GEOS4 | Bacteria | Firmicutes | Geobacillus_sp | 481743 | 2(KWH) |  |  |
| G4HG90_9BACL | Bacteria | Firmicutes | Bacillales | 1385 | 2(KWH) |  |  |
| V9G998_9BACL | Bacteria | Firmicutes | Bacillales | 1385 | 2(KWH) |  |  |
| A0A165Y0L0_9BACI | Bacteria | Firmicutes | Bacillaceae | 186817 | 2(KWH) |  |  |
| A0A0B0IGT8_9BACI | Bacteria | Firmicutes | Bacillaceae | 186817 | 2(KWH) |  |  |
| Q9K649_BACHD | Bacteria | Firmicutes | Bacillus_halodurans | 272558 | 2(KWH) |  |  |
| E5WR99_9BACI | Bacteria | Firmicutes | Bacillaceae | 186817 | 2(KWH) |  |  |
| D3FRS6_BACPE | Bacteria | Firmicutes | Bacillus_  pseudofirmus | 398511 | 2(KWH) |  |  |
| Q5WGM4_BACSK | Bacteria | Firmicutes | Bacillus_clausii | 66692 | 2(KWH) |  |  |
| A0A0M0KZX0_9BACI | Bacteria | Firmicutes | Bacillaceae | 186817 | 2(KWH) |  |  |
| A0A147KBN1_9BACI | Bacteria | Firmicutes | Bacillaceae | 186817 | 2(KWH) |  |  |
| W4ERL6_9BACL | Bacteria | Firmicutes | Bacillales | 1385 | 2(KWH) |  |  |
| A0A077J893_9BACI | Bacteria | Firmicutes | Bacillaceae | 186817 | 2(KWH) |  |  |
| A0A0W7Y8E0_9BACI | Bacteria | Firmicutes | Bacillaceae | 186817 | 2(KWH) |  |  |
| X2GXF4_9BACI | Bacteria | Firmicutes | Bacillaceae | 186817 | 2(KWH) |  |  |
| A0A0N0S3J2_9BACI | Bacteria | Firmicutes | Bacillaceae | 186817 | 2(KWH) |  |  |
| A0A0M9DGZ9_9BACI | Bacteria | Firmicutes | Bacillaceae | 186817 | 2(KWH) |  |  |
| A0A098EP32_9BACL | Bacteria | Firmicutes | Bacillales | 1385 | 2(KWH) |  |  |
| C0Z890_BREBN | Bacteria | Firmicutes | Brevibacillus_brevis | 358681 | 2(KWH) |  |  |
| J3BB78_9BACL | Bacteria | Firmicutes | Bacillales | 1385 | 2(KWH) |  |  |
| W4EQI4_9BACL | Bacteria | Firmicutes | Bacillales | 1385 | 2(NWF) |  |  |
| A0A0Q3WYY5_9BACI | Bacteria | Firmicutes | Bacillaceae | 186817 | 2(NWQ) |  |  |
| F5LH48_9BACL | Bacteria | Firmicutes | Bacillales | 1385 | 2(NYH) |  |  |
| A0A0P9CY60_9BACL | Bacteria | Firmicutes | Bacillales | 1385 | 2(NYH) |  |  |
| A0A0D3VFD1_9BACL | Bacteria | Firmicutes | Bacillales | 1385 | 2(RWH) |  |  |
| C6J0B9_9BACL | Bacteria | Firmicutes | Bacillales | 1385 | 2(RWH) |  |  |
| A0A090ZAP8_PAEMA | Bacteria | Firmicutes | Paenibacillus_  macerans | 44252 | 2(RWH) |  |  |
| A0A098MDH7_9BACL | Bacteria | Firmicutes | Bacillales | 1385 | 2(RWH) |  |  |
| A0A0U2W9R9_9BACL | Bacteria | Firmicutes | Bacillales | 1385 | 2(RWH) |  |  |
| H6NBK8_9BACL | Bacteria | Firmicutes | Bacillales | 1385 | 2(RWH) |  |  |
| A0A1C1A6W3_9BACL | Bacteria | Firmicutes | Bacillales | 1385 | 2(RWH) |  |  |
| A0A0Q9M2T8_9BACL | Bacteria | Firmicutes | Bacillales | 1385 | 2(RWH) |  |  |
| A0A0A2TXC3_9BACL | Bacteria | Firmicutes | Bacillales | 1385 | 2(RWH) |  |  |
| A0A089KXF0_9BACL | Bacteria | Firmicutes | Bacillales | 1385 | 2(RWH) |  |  |
| A0A101XXE6_9BACL | Bacteria | Firmicutes | Bacillales | 1385 | 2(RWH) |  |  |
| A0A0B0HT13_9BACL | Bacteria | Firmicutes | Bacillales | 1385 | 2(RWH) |  |  |
| A0A0A0E7R2_9BACI | Bacteria | Firmicutes | Bacillaceae | 186817 | 2(RWH) |  |  |
| HMOA_BACSU | Bacteria | Firmicutes | Bacillus_subtilis | 224308 | 2(RWH) |  |  |
| V6M971_9BACL | Bacteria | Firmicutes | Bacillales | 1385 | 2(RWH) |  |  |
| A0A0M4FU31_9BACI | Bacteria | Firmicutes | Bacillaceae | 186817 | 2(RWH) |  |  |
| A0A0K9H1J9_9BACI | Bacteria | Firmicutes | Bacillaceae | 186817 | 2(RWH) |  |  |
| A0A1E3L0E2_9BACL | Bacteria | Firmicutes | Bacillales | 1385 | 2(RWH) |  |  |
| A0A172ZIK3_9BACL | Bacteria | Firmicutes | Bacillales | 1385 | 2(RWH) |  |  |
| A0A0X8CQU1_9BACL | Bacteria | Firmicutes | Bacillales | 1385 | 2(RWH) |  |  |
| A0A069DA78_9BACL | Bacteria | Firmicutes | Bacillales | 1385 | 2(RWH) |  |  |
| A0A0A3J467_9BACI | Bacteria | Firmicutes | Bacillaceae | 186817 | 2(RWH) |  |  |
| A0A0M9X0H6_9BACI | Bacteria | Firmicutes | Bacillaceae | 186817 | 2(RWH) |  |  |
| F2F6A5_SOLSS | Bacteria | Firmicutes | Solibacillus_  silvestris | 1002809 | 2(RWH) |  |  |
| U5L5Z8_9BACI | Bacteria | Firmicutes | Bacillaceae | 186817 | 2(RWH) |  |  |
| A0A143HAZ2_9BACL | Bacteria | Firmicutes | Bacillales | 1385 | 2(RWH) |  |  |
| A0A1E7DKB7_9BACI | Bacteria | Firmicutes | Bacillaceae | 186817 | 2(SWH) |  |  |
| A0A0V8HGS6_9BACI | Bacteria | Firmicutes | Bacillaceae | 186817 | 2(SWH) |  |  |
| A0A0N8GH93_9BACI | Bacteria | Firmicutes | Bacillaceae | 186817 | 2(SWH) |  |  |
| A6CK76_9BACI | Bacteria | Firmicutes | Bacillaceae | 186817 | 2(SWH) |  |  |
| A0A0M0G691_9BACI | Bacteria | Firmicutes | Bacillaceae | 186817 | 2(SWH) |  |  |
| A0A0M9GT07_9BACI | Bacteria | Firmicutes | Bacillaceae | 186817 | 2(SWH) |  |  |
| A0A075JNZ2_9BACI | Bacteria | Firmicutes | Bacillaceae | 186817 | 2(SWH) |  |  |
| W4Q1K6_9BACI | Bacteria | Firmicutes | Bacillaceae | 186817 | 2(SWH) |  |  |
| W4QU03_BACA3 | Bacteria | Firmicutes | Bacillus_akibai | 1236973 | 2(SWH) |  |  |
| D3FSS8_BACPE | Bacteria | Firmicutes | Bacillus_  pseudofirmus | 398511 | 2(SWH) |  |  |
| U5LEG5_9BACI | Bacteria | Firmicutes | Bacillaceae | 186817 | 2(SWH) |  |  |
| A0A0A2TAH1_9BACI | Bacteria | Firmicutes | Bacillaceae | 186817 | 2(SWH) |  |  |
| A0A0A5FWU8_9BACI | Bacteria | Firmicutes | Bacillaceae | 186817 | 2(SWH) |  |  |
| I0JQ76_HALH3 | Bacteria | Firmicutes | Halobacillus_  halophilus | 866895 | 2(SWH) |  |  |
| L5N9C9_9BACI | Bacteria | Firmicutes | Bacillaceae | 186817 | 2(SWH) |  |  |
| A0A0B0D545_9BACI | Bacteria | Firmicutes | Bacillaceae | 186817 | 2(SWH) |  |  |
| K2GDC9_9BACI | Bacteria | Firmicutes | Bacillaceae | 186817 | 2(SWH) |  |  |
| I8J6H2_9BACI | Bacteria | Firmicutes | Bacillaceae | 186817 | 2(SWH) |  |  |
| D5DX46_BACMQ | Bacteria | Firmicutes | Bacillus_  megaterium | 545693 | 2(SWH) |  |  |
| A0A060M2B1_9BACI | Bacteria | Firmicutes | Bacillaceae | 186817 | 2(SWH) |  |  |
| A0A0K9HAY5_9BACI | Bacteria | Firmicutes | Bacillaceae | 186817 | 2(SWH) |  |  |
| A0A0J5QS38_9BACI | Bacteria | Firmicutes | Bacillaceae | 186817 | 2(SWH) |  |  |
| A0A061PCL7_9BACL | Bacteria | Firmicutes | Bacillales | 1385 | 2(SWH) |  |  |
| A0A061P2I0_9BACL | Bacteria | Firmicutes | Bacillales | 1385 | 2(SWH) |  |  |
| A0A0F5HMR0_9BACI | Bacteria | Firmicutes | Bacillaceae | 186817 | 2(SWH) |  |  |
| H6NTN3_9BACL | Bacteria | Firmicutes | Bacillales | 1385 | 2(SWH) |  |  |
| A0A172ZGC6_9BACL | Bacteria | Firmicutes | Bacillales | 1385 | 2(SWH) |  |  |
| W9B787_9BACI | Bacteria | Firmicutes | Bacillaceae | 186817 | 2(SWH) |  |  |
| A0A0U2XTX3_9BACL | Bacteria | Firmicutes | Bacillales | 1385 | 2(SWH) |  |  |
| A0A098ELL9_9BACL | Bacteria | Firmicutes | Bacillales | 1385 | 2(SWH) |  |  |
| Q3EZT5_BACTI | Bacteria | Firmicutes | Bacillus_  thuringiensis subsp | 1430 | 3(NWH) |  |  |
| Q81GW8_BACCR | Bacteria | Firmicutes | Bacillus_cereus | 226900 | 3(NWH) |  |  |
| Q81U25_BACAN | Bacteria | Firmicutes | Bacillus_anthracis | 1392 | 3(NWH) |  |  |
| C3BGZ8_9BACI | Bacteria | Firmicutes | Bacillaceae | 186817 | 3(NWH) |  |  |
| A0A073K0P6_9BACI | Bacteria | Firmicutes | Bacillaceae | 186817 | 3(NWH) |  |  |
| Q65LR6_BACLD | Bacteria | Firmicutes | Bacillus_  licheniformis | 279010 | 3(NWH) |  |  |
| HMOB_BACSU | Bacteria | Firmicutes | Bacillus_subtilis | 224308 | 3(NWH) |  |  |
| A0A084GJ82_9BACI | Bacteria | Firmicutes | Bacillaceae | 186817 | 3(NWH) |  |  |
| A0A176J6F3_9BACI | Bacteria | Firmicutes | Bacillaceae | 186817 | 3(NWH) |  |  |
| C5D6M3_GEOSW | Bacteria | Firmicutes | Geobacillus_sp | 471223 | 3(NWH) |  |  |
| A0A160FDJ3_9BACI | Bacteria | Firmicutes | Bacillaceae | 186817 | 3(NWH) |  |  |
| A0A023DET4_9BACI | Bacteria | Firmicutes | Bacillaceae | 186817 | 3(NWH) |  |  |
| A0A160F3A4_9BACI | Bacteria | Firmicutes | Bacillaceae | 186817 | 3(NWH) |  |  |
| A0A094LBK6_9BACI | Bacteria | Firmicutes | Bacillaceae | 186817 | 3(NWH) |  |  |
| B7GF20_ANOFW | Bacteria | Firmicutes | Anoxybacillus_  flavithermus | 491915 | 3(NWH) |  |  |
| A0A0M0LGX8_9BACI | Bacteria | Firmicutes | Bacillaceae | 186817 | 3(NWH) |  |  |
| A0A0V8JPG2_9BACI | Bacteria | Firmicutes | Bacillaceae | 186817 | 3(NWH) |  |  |
| D5DZ10_BACMQ | Bacteria | Firmicutes | Bacillus_  megaterium | 545693 | 3(NWH) |  |  |
| A0A0M9GT57_9BACI | Bacteria | Firmicutes | Bacillaceae | 186817 | 3(NWH) |  |  |
| A0A0H4KSL1_9BACI | Bacteria | Firmicutes | Bacillaceae | 186817 | 3(NWH) |  |  |
| A0A147KAF9_9BACI | Bacteria | Firmicutes | Bacillaceae | 186817 | 3(NWH) |  |  |
| U5L681_9BACI | Bacteria | Firmicutes | Bacillaceae | 186817 | 3(NWH) |  |  |
| A0A1E7DLK5_9BACI | Bacteria | Firmicutes | Bacillaceae | 186817 | 3(NWH) |  |  |
| A0A177LCL9_9BACL | Bacteria | Firmicutes | Bacillales | 1385 | 3(NWH) |  |  |
| A0A0D6ZCH9_9BACI | Bacteria | Firmicutes | Bacillaceae | 186817 | 3(NWH) |  |  |
| S2XZX7_9BACL | Bacteria | Firmicutes | Bacillales | 1385 | 3(NWH) |  |  |
| A6CQP3_9BACI | Bacteria | Firmicutes | Bacillaceae | 186817 | 3(NWH) |  |  |
| A0A0P6W9N5_9BACI | Bacteria | Firmicutes | Bacillaceae | 186817 | 3(NWH) |  |  |
| A0A0V8HI78_9BACI | Bacteria | Firmicutes | Bacillaceae | 186817 | 3(NWH) |  |  |
| A0A0M0GRK0_9BACI | Bacteria | Firmicutes | Bacillaceae | 186817 | 3(NWH) |  |  |
| A0A0C2V9I6_9BACL | Bacteria | Firmicutes | Bacillales | 1385 | 3(NWH) |  |  |
| A0A0B5APR3_9BACL | Bacteria | Firmicutes | Bacillales | 1385 | 3(NWH) |  |  |
| A0A0C2RZU0_9BACL | Bacteria | Firmicutes | Bacillales | 1385 | 3(NWH) |  |  |
| A0A0K9F0X1_9BACI | Bacteria | Firmicutes | Bacillaceae | 186817 | 3(NWH) |  |  |
| X2H0M4_9BACI | Bacteria | Firmicutes | Bacillaceae | 186817 | 3(NWH) |  |  |
| A0A0W7YQ34_9BACI | Bacteria | Firmicutes | Bacillaceae | 186817 | 3(NWH) |  |  |
| A0A0M9DKG4_9BACI | Bacteria | Firmicutes | Bacillaceae | 186817 | 3(NWH) |  |  |
| A0A0M8QEV7_9BACI | Bacteria | Firmicutes | Bacillaceae | 186817 | 3(NWH) |  |  |
| A0A0A3HTA3_9BACI | Bacteria | Firmicutes | Bacillaceae | 186817 | 3(NWH) |  |  |
| A0A0A3IAZ4_9BACI | Bacteria | Firmicutes | Bacillaceae | 186817 | 3(NWH) |  |  |
| A0A150YIB3_9BACI | Bacteria | Firmicutes | Bacillaceae | 186817 | 3(NWH) |  |  |
| A0A087N393_9BACI | Bacteria | Firmicutes | Bacillaceae | 186817 | 3(NWH) |  |  |
| A0A0B4RA39_9BACL | Bacteria | Firmicutes | Bacillales | 1385 | 3(NWH) |  |  |
| A0A0U2Q7B3_9BACL | Bacteria | Firmicutes | Bacillales | 1385 | 3(NWH) |  |  |
| A0A098EMF6_9BACL | Bacteria | Firmicutes | Bacillales | 1385 | 3(NWH) |  |  |
| A0A0F5I5J4_9BACI | Bacteria | Firmicutes | Bacillaceae | 186817 | 3(NWH) |  |  |
| A0A0K9H2T6_9BACI | Bacteria | Firmicutes | Bacillaceae | 186817 | 3(NWH) |  |  |
| A0A0A8JI35_BACSX | Bacteria | Firmicutes | Bacillus_sp | 98228 | 3(NWH) |  |  |
| A0A135WH95_9BACL | Bacteria | Firmicutes | Bacillales | 1385 | 3(NWH) |  |  |
| M7NDU8_9BACL | Bacteria | Firmicutes | Bacillales | 1385 | 3(NWH) |  |  |
| A0A098F640_9BACI | Bacteria | Firmicutes | Bacillaceae | 186817 | 3(NWH) |  |  |
| A0A0M4GFR5_9BACI | Bacteria | Firmicutes | Bacillaceae | 186817 | 3(NWH) |  |  |
| A0A0M2SPC9_9BACI | Bacteria | Firmicutes | Bacillaceae | 186817 | 3(NWH) |  |  |
| N0APC6_9BACI | Bacteria | Firmicutes | Bacillaceae | 186817 | 3(NWH) |  |  |
| A0A0M1NQC8_9BACI | Bacteria | Firmicutes | Bacillaceae | 186817 | 3(NWH) |  |  |
| A0A0A2TAW6_9BACI | Bacteria | Firmicutes | Bacillaceae | 186817 | 3(NWH) |  |  |
| A0A0A2UQ22_9BACI | Bacteria | Firmicutes | Bacillaceae | 186817 | 3(NWH) |  |  |
| A0A0A5FY70_9BACI | Bacteria | Firmicutes | Bacillaceae | 186817 | 3(NWH) |  |  |
| A0A0A5GN86_9BACI | Bacteria | Firmicutes | Bacillaceae | 186817 | 3(NWH) |  |  |
| I0JKB1_HALH3 | Bacteria | Firmicutes | Halobacillus_  halophilus | 866895 | 3(NWH) |  |  |
| A0A0B0D9E7_9BACI | Bacteria | Firmicutes | Bacillaceae | 186817 | 3(NWH) |  |  |
| A0A059NZ95_9BACI | Bacteria | Firmicutes | Bacillaceae | 186817 | 3(NWH) |  |  |
| L5NA23_9BACI | Bacteria | Firmicutes | Bacillaceae | 186817 | 3(NWH) |  |  |
| A0A024QEL8_9BACI | Bacteria | Firmicutes | Bacillaceae | 186817 | 3(NWH) |  |  |
| A0A0L0QTY2_VIRPA | Bacteria | Firmicutes | Virgibacillus_  pantothenticus | 1473 | 3(NWH) |  |  |
| A0A160IIV5_9BACI | Bacteria | Firmicutes | Bacillaceae | 186817 | 3(NWH) |  |  |
| A0A0V8JAY9_9BACI | Bacteria | Firmicutes | Bacillaceae | 186817 | 3(NWH) |  |  |
| I8AJW9_9BACI | Bacteria | Firmicutes | Bacillaceae | 186817 | 3(NWH) |  |  |
| W4Q1R3_9BACI | Bacteria | Firmicutes | Bacillaceae | 186817 | 3(NWH) |  |  |
| A0A0B0IMC5_9BACI | Bacteria | Firmicutes | Bacillaceae | 186817 | 3(NWH) |  |  |
| W4QUC6_BACA3 | Bacteria | Firmicutes | Bacillus_akibai | 1236973 | 3(NWH) |  |  |
| D3FVT5_BACPE | Bacteria | Firmicutes | Bacillus_  pseudofirmus | 398511 | 3(NWH) |  |  |
| A0A0U2PDV0_9BACL | Bacteria | Firmicutes | Bacillales | 1385 | 3(NWH) |  |  |
| A0A098EHV5_9BACL | Bacteria | Firmicutes | Bacillales | 1385 | 3(NWH) |  |  |
| A0A0B4REM9_9BACL | Bacteria | Firmicutes | Bacillales | 1385 | 3(NWH) |  |  |
| A0A172ZLV9_9BACL | Bacteria | Firmicutes | Bacillales | 1385 | 3(NWH) |  |  |
| A0A1E3L037_9BACL | Bacteria | Firmicutes | Bacillales | 1385 | 3(NWH) |  |  |
| A0A0A2U6Y8_9BACL | Bacteria | Firmicutes | Bacillales | 1385 | 3(NWH) |  |  |
| A0A069DIB5_9BACL | Bacteria | Firmicutes | Bacillales | 1385 | 3(NWH) |  |  |
| A0A075LJD0_9BACI | Bacteria | Firmicutes | Bacillaceae | 186817 | 3(NWH) |  |  |
| A0A075JN73_9BACI | Bacteria | Firmicutes | Bacillaceae | 186817 | 3(NWH) |  |  |
| W9AAU2_9BACI | Bacteria | Firmicutes | Bacillaceae | 186817 | 3(NWH) |  |  |
| A0A061P5Z4_9BACL | Bacteria | Firmicutes | Bacillales | 1385 | 3(NWH) |  |  |
| D3FV58_BACPE | Bacteria | Firmicutes | Bacillus_  pseudofirmus | 398511 | 3(NWH) |  |  |
| A0A0J6CT63_9BACI | Bacteria | Firmicutes | Bacillaceae | 186817 | 3(NWH) |  |  |
| A0A1E5LEK5_9BACI | Bacteria | Firmicutes | Bacillaceae | 186817 | 3(NWH) |  |  |
| W7YU30_9BACI | Bacteria | Firmicutes | Bacillaceae | 186817 | 3(NWH) |  |  |
| A0A060M1C3_9BACI | Bacteria | Firmicutes | Bacillaceae | 186817 | 3(NWH) |  |  |
| Q5WHZ8_BACSK | Bacteria | Firmicutes | Bacillus_clausii | 66692 | 3(NWH) |  |  |
| E6TTI9_BACCJ | Bacteria | Firmicutes | Bacillus_  cellulosilyticus | 649639 | 3(NWH) |  |  |
| A0A1D7QWV8_9BACI | Bacteria | Firmicutes | Bacillaceae | 186817 | 3(NWH) |  |  |
| D6XXL8_BACIE | Bacteria | Firmicutes | Bacillus_  selenitireducens | 439292 | 3(NWH) |  |  |
| A0A0D1XEQ0_ANEMI | Bacteria | Firmicutes | Aneurinibacillus_  migulanus | 47500 | 3(NWH) |  |  |
| A0A0X8D6B5_9BACL | Bacteria | Firmicutes | Bacillales | 1385 | 3(NWH) |  |  |
| A0A0Q3WG02_BRECH | Bacteria | Firmicutes | Brevibacillus_  choshinensis | 54911 | 3(NWH) |  |  |
| C0ZEI8_BREBN | Bacteria | Firmicutes | Brevibacillus_brevis | 358681 | 3(NWH) |  |  |
| J2QRN4_9BACL | Bacteria | Firmicutes | Bacillales | 1385 | 3(NWH) |  |  |
| V6M161_9BACL | Bacteria | Firmicutes | Bacillales | 1385 | 3(NWH) |  |  |
| A0A1A5XSH9_9BACL | Bacteria | Firmicutes | Bacillales | 1385 | 3(NWH) |  |  |
| M8DCD8_9BACL | Bacteria | Firmicutes | Bacillales | 1385 | 3(NWH) |  |  |
| A0A074LTF9_9BACL | Bacteria | Firmicutes | Bacillales | 1385 | 3(NWH) |  |  |
| HDOX1_STAA8 | Bacteria | Firmicutes | Staphylococcus_  aureus | 93061 | 3(NWH) |  |  |
| B9DIC2_STACT | Bacteria | Firmicutes | Staphylococcus_  carnosus | 396513 | 3(NWH) |  |  |
| K9ARK7_9STAP | Bacteria | Firmicutes | Staphylococcaceae | 90964 | 3(NWH) |  |  |
| A0A033UIU7_STAAU | Bacteria | Firmicutes | Staphylococcus_  aureus | 1280 | 3(NWH) |  |  |
| H0DF41_9STAP | Bacteria | Firmicutes | Staphylococcaceae | 90964 | 3(NWH) |  |  |
| HDOX2_STAA8 | Bacteria | Firmicutes | Staphylococcus_  aureus | 93061 | 3(NWH) |  |  |
| A0A0A8HSN4_STAHY | Bacteria | Firmicutes | Staphylococcus_  hyicus | 1284 | 3(NWH) |  |  |
| E8SGB4_STAPH | Bacteria | Firmicutes | Staphylococcus_  pseudintermedius | 937773 | 3(NWH) |  |  |
| A0A0A5G003_9BACI | Bacteria | Firmicutes | Bacillaceae | 186817 | 3(NWH) |  |  |
| A0A0X1RW94_9BACL | Bacteria | Firmicutes | Bacillales | 1385 | 3(NWH) |  |  |
| B7GLW7_ANOFW | Bacteria | Firmicutes | Anoxybacillus_  flavithermus | 491915 | 3(NWH) |  |  |
| A0A0B0HN95_9BACI | Bacteria | Firmicutes | Bacillaceae | 186817 | 3(NWH) |  |  |
| A0A165Y103_9BACI | Bacteria | Firmicutes | Bacillaceae | 186817 | 3(NWH) |  |  |
| C6D4S2_PAESJ | Bacteria | Firmicutes | Paenibacillus_sp | 324057 | 3(NWH) |  |  |
| A0A1B8WF14_9BACI | Bacteria | Firmicutes | Bacillaceae | 186817 | 3(NWH) |  |  |
| A0A0Q9L3G6_9BACL | Bacteria | Firmicutes | Bacillales | 1385 | 3(NWH) |  |  |
| A0A0Q4QZ50_9BACL | Bacteria | Firmicutes | Bacillales | 1385 | 3(NWH) |  |  |
| A0A172TIU4_9BACL | Bacteria | Firmicutes | Bacillales | 1385 | 3(NWH) |  |  |
| A0A098ME35_9BACL | Bacteria | Firmicutes | Bacillales | 1385 | 3(NWH) |  |  |
| A0A0E4HII4_9BACL | Bacteria | Firmicutes | Bacillales | 1385 | 3(NWH) |  |  |
| A0A089ISN0_9BACL | Bacteria | Firmicutes | Bacillales | 1385 | 3(NWH) |  |  |
| A0A1B8VUE2_9BACI | Bacteria | Firmicutes | Bacillaceae | 186817 | 3(NWH) |  |  |
| X5A6L5_9BACL | Bacteria | Firmicutes | Bacillales | 1385 | 3(NWH) |  |  |
| A0A101XVJ3_9BACL | Bacteria | Firmicutes | Bacillales | 1385 | 3(NWH) |  |  |
| A0A089LYL2_9BACL | Bacteria | Firmicutes | Bacillales | 1385 | 3(NWH) |  |  |
| V9GA49_9BACL | Bacteria | Firmicutes | Bacillales | 1385 | 3(NWH) |  |  |
| D3EG83_GEOS4 | Bacteria | Firmicutes | Geobacillus_sp | 481743 | 3(NWH) |  |  |
| G4HCL9_9BACL | Bacteria | Firmicutes | Bacillales | 1385 | 3(NWH) |  |  |
| A0A0M2VSB6_9BACL | Bacteria | Firmicutes | Bacillales | 1385 | 3(NWH) |  |  |
| K4ZNN3_PAEAL | Bacteria | Firmicutes | Paenibacillus_alvei | 44250 | 3(NWH) |  |  |
| H3SA54_9BACL | Bacteria | Firmicutes | Bacillales | 1385 | 3(NWH) |  |  |
| W7YNV8_9BACL | Bacteria | Firmicutes | Bacillales | 1385 | 3(NWH) |  |  |
| V9W8P9_9BACL | Bacteria | Firmicutes | Bacillales | 1385 | 3(NWH) |  |  |
| A0A087N2X2_9BACI | Bacteria | Firmicutes | Bacillaceae | 186817 | 3(NWH) |  |  |
| A0A127W3I6_SPOPS | Bacteria | Firmicutes | Sporosarcina_  psychrophila | 1476 | 3(NWH) |  |  |
| A0A0F7HHW9_9STAP | Bacteria | Firmicutes | Staphylococcaceae | 90964 | 3(NWH) |  |  |
| A0A0A1MRH4_9BACI | Bacteria | Firmicutes | Bacillaceae | 186817 | 3(NWH) |  |  |
| A0A075JMY4_9BACI | Bacteria | Firmicutes | Bacillaceae | 186817 | 3(NWH) |  |  |
| A0A0A8JKG5_BACSX | Bacteria | Firmicutes | Bacillus_sp | 98228 | 3(NWH) |  |  |
| W4QJZ4_9BACI | Bacteria | Firmicutes | Bacillaceae | 186817 | 3(NWH) |  |  |
| A0A094XEH3_BACAO | Bacteria | Firmicutes | Bacillus_  alcalophilus | 1445 | 3(NWH) |  |  |
| A0A0J5R0C6_9BACI | Bacteria | Firmicutes | Bacillaceae | 186817 | 3(NWH) |  |  |
| HDOX_BACHD | Bacteria | Firmicutes | Bacillus_halodurans | 272558 | 3(NWH) |  |  |
| HDOX_BACCR | Bacteria | Firmicutes | Bacillus_cereus | 226900 | 3(NWH) |  |  |
| A0A1A5YRT1_9BACL | Bacteria | Firmicutes | Bacillales | 1385 | 3(NWH) |  |  |
| C6CXI8_PAESJ | Bacteria | Firmicutes | Paenibacillus_sp | 324057 | 3(NWH) |  |  |
| A0A0U2W3V3_9BACL | Bacteria | Firmicutes | Bacillales | 1385 | 3(NWH) |  |  |
| A0A081NTU9_9BACL | Bacteria | Firmicutes | Bacillales | 1385 | 3(NWH) |  |  |
| E3E6U8_PAEPS | Bacteria | Firmicutes | Paenibacillus_  polymyxa | 886882 | 3(NWH) |  |  |
| A0A090Z770_PAEMA | Bacteria | Firmicutes | Paenibacillus_  macerans | 44252 | 3(NWH) |  |  |
| A0A172ZKW0_9BACL | Bacteria | Firmicutes | Bacillales | 1385 | 3(NWH) |  |  |
| A0A1E3L9N0_9BACL | Bacteria | Firmicutes | Bacillales | 1385 | 3(NWH) |  |  |
| A0A089KXY8_9BACL | Bacteria | Firmicutes | Bacillales | 1385 | 3(NWH) |  |  |
| W7ZG88_9BACI | Bacteria | Firmicutes | Bacillaceae | 186817 | 3(NWH) |  |  |
| HDOX_BACSK | Bacteria | Firmicutes | Bacillus_clausii | 66692 | 3(NWH) |  |  |
| A0A060LXF3_9BACI | Bacteria | Firmicutes | Bacillaceae | 186817 | 3(NWH) |  |  |
| A0A061PCH7_9BACL | Bacteria | Firmicutes | Bacillales | 1385 | 3(NWH) |  |  |
| A0A0K9GXP1_9BACI | Bacteria | Firmicutes | Bacillaceae | 186817 | 3(NWH) |  |  |
| A0A0D0SMA5_STAGA | Bacteria | Firmicutes | Staphylococcus_  gallinarum | 1293 | 3(NWH) |  |  |
| H0DFD1_9STAP | Bacteria | Firmicutes | Staphylococcaceae | 90964 | 3(NWH) |  |  |
| HDOX_STAEQ | Bacteria | Firmicutes | Staphylococcus_  epidermidis | 176279 | 3(NWH) |  |  |
| A0A0M0L5X0_9BACI | Bacteria | Firmicutes | Bacillaceae | 186817 | 3(NWH) |  |  |
| A0A0U2Z734_9BACL | Bacteria | Firmicutes | Bacillales | 1385 | 3(NWH) |  |  |
| A0A098EHK9_9BACL | Bacteria | Firmicutes | Bacillales | 1385 | 3(NWH) |  |  |
| A0A094WRD1_BACAO | Bacteria | Firmicutes | Bacillus_  alcalophilus | 1445 | 3(NWH) |  |  |
| A0A143HGY7_9BACL | Bacteria | Firmicutes | Bacillales | 1385 | 3(NWH) |  |  |
| W4F3U8_9BACL | Bacteria | Firmicutes | Bacillales | 1385 | 3(NWH) |  |  |
| A0A109Q904_9BACL | Bacteria | Firmicutes | Bacillales | 1385 | 3(NWH) |  |  |
| A0A0A0E503_9BACI | Bacteria | Firmicutes | Bacillaceae | 186817 | 3(NWH) |  |  |
| B9E9V0_MACCJ | Bacteria | Firmicutes | Macrococcus_  caseolyticus | 458233 | 3(NWH) |  |  |
| A0A033UDM6_STAAU | Bacteria | Firmicutes | Staphylococcus_  aureus | 1280 | 3(NWH) |  |  |
| A0A0N0CUB3_9BACI | Bacteria | Firmicutes | Bacillaceae | 186817 | 3(NWH) |  |  |
| A0A0W7Y2H8_9BACI | Bacteria | Firmicutes | Bacillaceae | 186817 | 3(NWH) |  |  |
| A0A0K9FHD9_9BACI | Bacteria | Firmicutes | Bacillaceae | 186817 | 3(NWH) |  |  |
| X2GY49_9BACI | Bacteria | Firmicutes | Bacillaceae | 186817 | 3(NWH) |  |  |
| A0A0A3IG79_9BACI | Bacteria | Firmicutes | Bacillaceae | 186817 | 3(NWH) |  |  |
| F2FAT2_SOLSS | Bacteria | Firmicutes | Solibacillus_  silvestris | 1002809 | 3(NWH) |  |  |
| A0A168Q1M1_9BACL | Bacteria | Firmicutes | Bacillales | 1385 | 3(NWH) |  |  |
| A0A167BAI0_9BACL | Bacteria | Firmicutes | Bacillales | 1385 | 3(NWH) |  |  |
| A0A0M1NS72_9BACI | Bacteria | Firmicutes | Bacillaceae | 186817 | 3(NWH) |  |  |
| B1YG40_EXIS2 | Bacteria | Firmicutes | Exiguobacterium_  sibiricum | 262543 | 3(NWH) |  |  |
| A0A1C0YLB4_9BACL | Bacteria | Firmicutes | Bacillales | 1385 | 3(NWH) |  |  |
| A0A1B9AIK4_9BACI | Bacteria | Firmicutes | Bacillaceae | 186817 | 3(NWH) |  |  |
| A0A0K9GCA9_9BACI | Bacteria | Firmicutes | Bacillaceae | 186817 | 3(NWH) |  |  |
| B9DM71_STACT | Bacteria | Firmicutes | Staphylococcus_  carnosus | 396513 | 3(NWH) |  |  |
| A0A0M8Q3Q9_9BACI | Bacteria | Firmicutes | Bacillaceae | 186817 | 3(NWH) |  |  |
| A0A1C0YDF8_9BACL | Bacteria | Firmicutes | Bacillales | 1385 | 3(NWH) |  |  |
| A0A150YFW9_9BACI | Bacteria | Firmicutes | Bacillaceae | 186817 | 3(NWH) |  |  |
| A0A0R2HW60_CARDV | Bacteria | Firmicutes | Carnobacterium_  divergens | 2748 | 3(NWH) |  |  |
| W7C1E8_9LIST | Bacteria | Firmicutes | Listeriaceae | 186820 | 3(NWH) |  |  |
| W7B0I4_9LIST | Bacteria | Firmicutes | Listeriaceae | 186820 | 3(NWH) |  |  |
| W7CSV2_BROTH | Bacteria | Firmicutes | Brochothrix_  thermosphacta | 2756 | 3(NWH) |  |  |
| K8ELZ5_CARML | Bacteria | Firmicutes | Carnobacterium_  maltaromaticum | 2751 | 3(NWH) |  |  |
| E6LHR8_9ENTE | Bacteria | Firmicutes | Enterococcaceae | 81852 | 3(NWH) |  |  |
| S0KV52_9ENTE | Bacteria | Firmicutes | Enterococcaceae | 81852 | 3(NWH) |  |  |
| C4L0B6_EXISA | Bacteria | Firmicutes | Exiguobacterium_  sp | 360911 | 3(NWH) |  |  |
| B1YGM6_EXIS2 | Bacteria | Firmicutes | Exiguobacterium_  sibiricum | 262543 | 3(NWH) |  |  |
| A0A0N0HFV4_THEVU | Bacteria | Firmicutes | Thermoactinomyces_vulgaris | 2026 | 3(NWH) |  |  |
| F5SIK2_9BACL | Bacteria | Firmicutes | Bacillales | 1385 | 3(NWH) |  |  |
| D7UWG3_LISGR | Bacteria | Firmicutes | Listeria_grayi | 1641 | 3(NWH) |  |  |
| A0A0A8X505_9BACI | Bacteria | Firmicutes | Bacillaceae | 186817 | na |  | failed iqtree; removed from phylogenetic analysis |
| A0A127W1Z9_SPOPS | Bacteria | Firmicutes | Sporosarcina_  psychrophila | 1476 | na |  | failed iqtree; removed from phylogenetic analysis |
| A0A0A3J6N7_9BACI | Bacteria | Firmicutes | Bacillaceae | 186817 | na |  | failed iqtree; removed from phylogenetic analysis |
| A0A0K9GS06_9BACI | Bacteria | Firmicutes | Bacillaceae | 186817 | na |  | failed iqtree; removed from phylogenetic analysis |
| A0A0H4PEM5_9BACI | Bacteria | Firmicutes | Bacillaceae | 186817 | na |  | failed iqtree; removed from phylogenetic analysis |
| A0A0Q3TLX0_9BACI | Bacteria | Firmicutes | Bacillaceae | 186817 | na |  | failed iqtree; removed from phylogenetic analysis |
| K2FL47_9BACI | Bacteria | Firmicutes | Bacillaceae | 186817 | na |  | failed iqtree; removed from phylogenetic analysis |
| A0A179SRN8_9BACI | Bacteria | Firmicutes | Bacillaceae | 186817 | na |  | failed iqtree; removed from phylogenetic analysis |
| A0A0M2PHP3_9BACI | Bacteria | Firmicutes | Bacillaceae | 186817 | na |  | failed iqtree; removed from phylogenetic analysis |
| W9AII1_9BACI | Bacteria | Firmicutes | Bacillaceae | 186817 | na |  | failed iqtree; removed from phylogenetic analysis |
| A0A0M4FVC5_9BACI | Bacteria | Firmicutes | Bacillaceae | 186817 | na |  | failed iqtree; removed from phylogenetic analysis |
| A0A0A1MWT7_9BACI | Bacteria | Firmicutes | Bacillaceae | 186817 | na |  | failed iqtree; removed from phylogenetic analysis |
| Q8ERY1_OCEIH | Bacteria | Firmicutes | Oceanobacillus_  iheyensis | 221109 | na |  | failed iqtree; removed from phylogenetic analysis |
| W1SNN8_9BACI | Bacteria | Firmicutes | Bacillaceae | 186817 | na |  | failed iqtree; removed from phylogenetic analysis |
| G2TP25_BACCO | Bacteria | Firmicutes | Bacillus_coagulans | 1398 | na |  | failed iqtree; removed from phylogenetic analysis |
| A0A0A6VGY2_9BACI | Bacteria | Firmicutes | Bacillaceae | 186817 | na |  | failed iqtree; removed from phylogenetic analysis |
| A0A0M0X659_9BACI | Bacteria | Firmicutes | Bacillaceae | 186817 | na |  | failed iqtree; removed from phylogenetic analysis |
| A0A165YGU6_9BACI | Bacteria | Firmicutes | Bacillaceae | 186817 | na |  | failed iqtree; removed from phylogenetic analysis |
| A0A0L0QMT2_VIRPA | Bacteria | Firmicutes | Virgibacillus_  pantothenticus | 1473 | na |  | failed iqtree; removed from phylogenetic analysis |
| A0A061PEL7_9BACL | Bacteria | Firmicutes | Bacillales | 1385 | na |  | failed iqtree; removed from phylogenetic analysis |
| A0A0D5NJZ5_9BACL | Bacteria | Firmicutes | Bacillales | 1385 | na |  | failed iqtree; removed from phylogenetic analysis |
| A0A0F5R950_9BACL | Bacteria | Firmicutes | Bacillales | 1385 | na |  | failed iqtree; removed from phylogenetic analysis |
| A0A147KBP7_9BACI | Bacteria | Firmicutes | Bacillaceae | 186817 | na |  | failed iqtree; removed from phylogenetic analysis |
| A0A0U2MCR9_9BACL | Bacteria | Firmicutes | Bacillales | 1385 | na |  | failed iqtree; removed from phylogenetic analysis |
| W7YBT6_9BACL | Bacteria | Firmicutes | Bacillales | 1385 | na |  | failed iqtree; removed from phylogenetic analysis |
| X0RKQ6_9BACI | Bacteria | Firmicutes | Bacillaceae | 186817 | na |  | failed iqtree; removed from phylogenetic analysis |
| A0A0Q9Y6W1_9BACI | Bacteria | Firmicutes | Bacillaceae | 186817 | na |  | failed iqtree; removed from phylogenetic analysis |
| A0A0Q9YJS8_9BACI | Bacteria | Firmicutes | Bacillaceae | 186817 | na |  | failed iqtree; removed from phylogenetic analysis |
| A0A075LN66_9BACI | Bacteria | Firmicutes | Bacillaceae | 186817 | na |  | failed iqtree; removed from phylogenetic analysis |
| HDOX_STAS1 | Bacteria | Firmicutes | Staphylococcus_  saprophyticus subsp | 342451 | na |  | failed iqtree; removed from phylogenetic analysis |
| A0A0Q3W0P3_9BACI | Bacteria | Firmicutes | Bacillaceae | 186817 | na |  | failed iqtree; removed from phylogenetic analysis |
| A0A0J5QVM0_9BACI | Bacteria | Firmicutes | Bacillaceae | 186817 | na |  | failed iqtree; removed from phylogenetic analysis |
| H7F935_9LIST | Bacteria | Firmicutes | Listeriaceae | 186820 | na |  | failed iqtree; removed from phylogenetic analysis |
| D7UX71_LISGR | Bacteria | Firmicutes | Listeria_grayi | 1641 | na |  | failed iqtree; removed from phylogenetic analysis |
| R2T3V6_9ENTE | Bacteria | Firmicutes | Enterococcaceae | 81852 | na |  | failed iqtree; removed from phylogenetic analysis |
| A0A1E5GHP0_9ENTE | Bacteria | Firmicutes | Enterococcaceae | 81852 | na |  | failed iqtree; removed from phylogenetic analysis |
| W4QUC0_BACA3 | Bacteria | Firmicutes | Bacillus_akibai | 1236973 | na |  | failed iqtree; removed from phylogenetic analysis |
| A0A167G471_9BACL | Bacteria | Firmicutes | Bacillales | 1385 | na |  | failed iqtree; removed from phylogenetic analysis |
| A0A168J641_9BACL | Bacteria | Firmicutes | Bacillales | 1385 | na |  | failed iqtree; removed from phylogenetic analysis |
| E6TVW7_BACCJ | Bacteria | Firmicutes | Bacillus_  cellulosilyticus | 649639 | na |  | failed iqtree; removed from phylogenetic analysis |
| F5LEQ5_9BACL | Bacteria | Firmicutes | Bacillales | 1385 | na |  | failed iqtree; removed from phylogenetic analysis |
| A0A0D1XUU2_ANEMI | Bacteria | Firmicutes | Aneurinibacillus_  migulanus | 47500 | na |  | failed iqtree; removed from phylogenetic analysis |
| K4ZMN9_PAEAL | Bacteria | Firmicutes | Paenibacillus_alvei | 44250 | na |  | failed iqtree; removed from phylogenetic analysis |
| A0A1A5XRX8_9BACL | Bacteria | Firmicutes | Bacillales | 1385 | na |  | failed iqtree; removed from phylogenetic analysis |
| A0A075RBM5_BRELA | Bacteria | Firmicutes | Brevibacillus_  laterosporus | 1465 | na |  | failed iqtree; removed from phylogenetic analysis |
| A0A0Q3S0D5_9BACI | Bacteria | Firmicutes | Bacillaceae | 186817 | na |  | failed iqtree; removed from phylogenetic analysis |
| A0A0K9FI75_9BACI | Bacteria | Firmicutes | Bacillaceae | 186817 | na |  | failed iqtree; removed from phylogenetic analysis |
| A0A0A8X2K0_9BACI | Bacteria | Firmicutes | Bacillaceae | 186817 | na |  | failed iqtree; removed from phylogenetic analysis |
| W4QEP1_9BACI | Bacteria | Firmicutes | Bacillaceae | 186817 | na |  | failed iqtree; removed from phylogenetic analysis |
| A0A0M2SW87_9BACI | Bacteria | Firmicutes | Bacillaceae | 186817 | na |  | failed iqtree; removed from phylogenetic analysis |
| A0A150L1D3_9BACI | Bacteria | Firmicutes | Bacillaceae | 186817 | na |  | failed iqtree; removed from phylogenetic analysis |
| A0A0M1NRB8_9BACI | Bacteria | Firmicutes | Bacillaceae | 186817 | na |  | failed iqtree; removed from phylogenetic analysis |
| A0A0M0XBX0_9BACI | Bacteria | Firmicutes | Bacillaceae | 186817 | na |  | failed iqtree; removed from phylogenetic analysis |
| A0A0Q3SZK8_BRECH | Bacteria | Firmicutes | Brevibacillus_  choshinensis | 54911 | na |  | failed iqtree; removed from phylogenetic analysis |
| W4Q784_9BACI | Bacteria | Firmicutes | Bacillaceae | 186817 | na |  | failed iqtree; removed from phylogenetic analysis |
| A0A0F5R109_9BACL | Bacteria | Firmicutes | Bacillales | 1385 | na |  | failed iqtree; removed from phylogenetic analysis |
| A0A0K9G9J4_9BACI | Bacteria | Firmicutes | Bacillaceae | 186817 | na |  | failed iqtree; removed from phylogenetic analysis |
| A0A1B9AMJ8_9BACI | Bacteria | Firmicutes | Bacillaceae | 186817 | na |  | failed iqtree; removed from phylogenetic analysis |
| A0A0A3IMY4_9BACI | Bacteria | Firmicutes | Bacillaceae | 186817 | na |  | failed iqtree; removed from phylogenetic analysis |
| A0A0A3HSG0_9BACI | Bacteria | Firmicutes | Bacillaceae | 186817 | na |  | failed iqtree; removed from phylogenetic analysis |
| A0A0K9GYI4_9BACI | Bacteria | Firmicutes | Bacillaceae | 186817 | na |  | failed iqtree; removed from phylogenetic analysis |
| A0A1E7DVL1_9BACI | Bacteria | Firmicutes | Bacillaceae | 186817 | na |  | failed iqtree; removed from phylogenetic analysis |
| E6SMP9_THEM7 | Bacteria | Firmicutes | Thermaerobacter_  marianensis | 644966 | na |  | failed iqtree; removed from phylogenetic analysis |
| A0A084GW15_9BACI | Bacteria | Firmicutes | Bacillaceae | 186817 | na |  | failed iqtree; removed from phylogenetic analysis |
| X0RBC2_9BACI | Bacteria | Firmicutes | Bacillaceae | 186817 | na |  | failed iqtree; removed from phylogenetic analysis |
| A0A0D0SQW0_STAGA | Bacteria | Firmicutes | Staphylococcus_  gallinarum | 1293 | na |  | failed iqtree; removed from phylogenetic analysis |
| Q2FV13_STAA8 | Bacteria | Firmicutes | Staphylococcus_  aureus | 93061 | na |  | failed iqtree; removed from phylogenetic analysis |
| Q5HL14_STAEQ | Bacteria | Firmicutes | Staphylococcus_  epidermidis | 176279 | na |  | failed iqtree; removed from phylogenetic analysis |
| A0A133Q6N2_STALU | Bacteria | Firmicutes | Staphylococcus_  lugdunensis | 28035 | na |  | failed iqtree; removed from phylogenetic analysis |
| A0A0D1VE95_ANEMI | Bacteria | Firmicutes | Aneurinibacillus_  migulanus | 47500 | na |  | failed iqtree; removed from phylogenetic analysis |
| A0A179T016_9BACI | Bacteria | Firmicutes | Bacillaceae | 186817 | na |  | failed iqtree; removed from phylogenetic analysis |
| A0A0M2PMN6_9BACI | Bacteria | Firmicutes | Bacillaceae | 186817 | na |  | failed iqtree; removed from phylogenetic analysis |
| R2SIR2_9ENTE | Bacteria | Firmicutes | Enterococcaceae | 81852 | na |  | failed iqtree; removed from phylogenetic analysis |
| A0A073JXS6_9BACI | Bacteria | Firmicutes | Bacillaceae | 186817 | na |  | failed iqtree; removed from phylogenetic analysis |
| E5WEM3_9BACI | Bacteria | Firmicutes | Bacillaceae | 186817 | na |  | failed iqtree; removed from phylogenetic analysis |
| I3E7G7_BACMT | Bacteria | Firmicutes | Bacillus_  methanolicus | 1471 | na |  | failed iqtree; removed from phylogenetic analysis |
| A0A0A5HM81_9BACI | Bacteria | Firmicutes | Bacillaceae | 186817 | na |  | failed iqtree; removed from phylogenetic analysis |
| A0A075JRI1_9BACI | Bacteria | Firmicutes | Bacillaceae | 186817 | na |  | failed iqtree; removed from phylogenetic analysis |
| A0A0U3W677_9BACI | Bacteria | Firmicutes | Bacillaceae | 186817 | na |  | failed iqtree; removed from phylogenetic analysis |
| A0A0J6CVW4_9BACI | Bacteria | Firmicutes | Bacillaceae | 186817 | na |  | failed iqtree; removed from phylogenetic analysis |
| N4W5X2_9BACI | Bacteria | Firmicutes | Bacillaceae | 186817 | na |  | failed iqtree; removed from phylogenetic analysis |
| A0A0W7YP89_9BACI | Bacteria | Firmicutes | Bacillaceae | 186817 | na |  | failed iqtree; removed from phylogenetic analysis |
| H6NI20_9BACL | Bacteria | Firmicutes | Bacillales | 1385 | na |  | failed iqtree; removed from phylogenetic analysis |
| G2TKN9_BACCO | Bacteria | Firmicutes | Bacillus_coagulans | 1398 | na |  | failed iqtree; removed from phylogenetic analysis |
| A0A176J7V1_9BACI | Bacteria | Firmicutes | Bacillaceae | 186817 | na |  | failed iqtree; removed from phylogenetic analysis |
| A0A0M2STW3_9BACI | Bacteria | Firmicutes | Bacillaceae | 186817 | na |  | failed iqtree; removed from phylogenetic analysis |
| E8SI76_STAPH | Bacteria | Firmicutes | Staphylococcus_  pseudintermedius | 937773 | na |  | failed iqtree; removed from phylogenetic analysis |
| A0A078M7T3_9STAP | Bacteria | Firmicutes | Staphylococcaceae | 90964 | na |  | failed iqtree; removed from phylogenetic analysis |
| A0A090IX75_9BACI | Bacteria | Firmicutes | Bacillaceae | 186817 | na |  | failed iqtree; removed from phylogenetic analysis |
| F5WRX8_ERYRF | Bacteria | Firmicutes | Erysipelothrix_rhusiopathiae | 650150 | na |  | failed iqtree; removed from phylogenetic analysis |
| I0ILF5_LEPFC | Bacteria | Nitrospirae | Leptospirillum_  ferrooxidans | 1162668 | 3(NWH) |  |  |
| J9ZA06_LEPFM | Bacteria | Nitrospirae | Leptospirillum_  ferriphilum | 1048260 | 3(NWH) |  |  |
| A0A0G4GEP9_VITBC | Eukaryote | other_Alveolata | Vitrella_  brassicaformis | 1169540 | 1(TFH) |  | N terminal ABM domain |
| A0A0G4GEP9_VITBC | Eukaryote | other_Alveolata | Vitrella_  brassicaformis | 1169540 | 3(NWH) |  | C terminal ABM domain |
| A0A136P287_9BACT | Bacteria | other_Bacteria | Bacteria | 2 | 2(-WH) |  |  |
| A0A0E3ZHP3_9BACT | Bacteria | other_Bacteria | Bacteria | 2 | 2(SWH) |  |  |
| J1FFB7_9BACT | Bacteria | other_Bacteria | Bacteria | 2 | 2(SWH) |  |  |
| A0A0P0C6U1_9BACT | Bacteria | other_Bacteria | Bacteria | 2 | 2(SWH) |  |  |
| A0A127B3Z1_9BACT | Bacteria | other_Bacteria | Bacteria | 2 | 2(SWH) |  |  |
| W4LDS2_9BACT | Bacteria | other_Bacteria | Bacteria | 2 | 3(NWH) |  |  |
| W4L9X6_9BACT | Bacteria | other_Bacteria | Bacteria | 2 | 3(NWH) |  |  |
| T0XVM2_9BACT | Bacteria | other_Bacteria | Bacteria | 2 | 3(NWH) |  |  |
| C6HXU1_9BACT | Bacteria | other_Bacteria | Bacteria | 2 | 3(NWH) |  |  |
| W4LYY6_9BACT | Bacteria | other_Bacteria | Bacteria | 2 | 3(NWH) |  |  |
| W4LTU4_9BACT | Bacteria | other_Bacteria | Bacteria | 2 | 3(NWH) |  |  |
| D5MMH7_9BACT | Bacteria | other_Bacteria | Bacteria | 2 | 3(NWH) |  |  |
| D5MJI1_9BACT | Bacteria | other_Bacteria | Bacteria | 2 | 3(NWH) |  |  |
| A0A142L7F6_9BACT | Bacteria | other_Bacteria | Bacteria | 2 | 3(NWH) |  |  |
| A0A136L2D3_9BACT | Bacteria | other_Bacteria | Bacteria | 2 | 3(NWH) |  |  |
| A0A191TLC1_9BACT | Bacteria | other_Bacteria | Bacteria | 2 | na |  | failed iqtree; removed from phylogenetic analysis |
| W0RBW4_9BACT | Bacteria | other_Bacteria | Bacteria | 2 | na |  | failed iqtree; removed from phylogenetic analysis |
| F0XXQ6_AURAN | Eukaryote | other_Stramenopiles | Aureococcus_  anophagefferens | 44056 | 2(NWQ) |  | failed iqtree; but retained eukaryotic sequence |
| Q09CQ8_STIAD | Bacteria | Proteobacteria | Stigmatella_  aurantiaca | 378806 | 0(SYK) |  |  |
| A0A0G2ZMH8_9DELT | Bacteria | Proteobacteria | Deltaproteobacteria | 28221 | 1(NYK) |  |  |
| A0A0F6W4E3_9DELT | Bacteria | Proteobacteria | Deltaproteobacteria | 28221 | 1(SYH) |  |  |
| F2J5R3_POLGS | Bacteria | Proteobacteria | Polymorphum_  gilvum | 991905 | 2(-WH) |  |  |
| A0A080KCX7_9GAMM | Bacteria | Proteobacteria | Gammaproteobacteria | 1236 | 2(-WH) |  |  |
| A7BUJ5_9GAMM | Bacteria | Proteobacteria | Gammaproteobacteria | 1236 | 2(SWH) |  |  |
| W6KB90_9PROT | Bacteria | Proteobacteria | Proteobacteria | 1224 | 2(SWH) |  |  |
| A0A0C1ZMS9_9DELT | Bacteria | Proteobacteria | Deltaproteobacteria | 28221 | 2(SWH) |  |  |
| L0DUM1_THIND | Bacteria | Proteobacteria | Thioalkalivibrio_  nitratireducens | 1255043 | 2(SWH) |  |  |
| A0A0S2TDM1_9GAMM | Bacteria | Proteobacteria | Gammaproteobacteria | 1236 | 3(NWH) |  |  |
| Q1ZR24_PHOAS | Bacteria | Proteobacteria | Photobacterium_  angustum | 314292 | 3(NWH) |  |  |
| A0A0D8PM30_9GAMM | Bacteria | Proteobacteria | Gammaproteobacteria | 1236 | 3(NWH) |  |  |
| C5BP80_TERTT | Bacteria | Proteobacteria | Teredinibacter_  turnerae | 377629 | 3(NWH) |  |  |
| Q0VNS0_ALCBS | Bacteria | Proteobacteria | Alcanivorax_  borkumensis | 393595 | 3(NWH) |  |  |
| A0A1A8TF63_9GAMM | Bacteria | Proteobacteria | Gammaproteobacteria | 1236 | 3(NWH) |  |  |
| F2JTU2_MARM1 | Bacteria | Proteobacteria | Marinomonas_  mediterranea | 717774 | 3(NWH) |  |  |
| A0A0F4QLW5_9GAMM | Bacteria | Proteobacteria | Gammaproteobacteria | 1236 | 3(NWH) |  |  |
| A0A0P8AS74_9GAMM | Bacteria | Proteobacteria | Gammaproteobacteria | 1236 | 3(NWH) |  |  |
| A0A085DVL3_9GAMM | Bacteria | Proteobacteria | Gammaproteobacteria | 1236 | 3(NWH) |  |  |
| A0A0D7UV92_9GAMM | Bacteria | Proteobacteria | Gammaproteobacteria | 1236 | 3(NWH) |  |  |
| A0A060B440_9GAMM | Bacteria | Proteobacteria | Gammaproteobacteria | 1236 | 3(NWH) |  |  |
| A0A101D125_9GAMM | Bacteria | Proteobacteria | Gammaproteobacteria | 1236 | 3(NWH) |  |  |
| A0A081G4E7_9GAMM | Bacteria | Proteobacteria | Gammaproteobacteria | 1236 | 3(NWH) |  |  |
| N6WYY9_9ALTE | Bacteria | Proteobacteria | Alteromonadaceae | 72275 | 3(NWH) |  |  |
| Q2BJL2_NEPCE | Bacteria | Proteobacteria | Neptuniibacter_  caesariensis | 207954 | 3(NWH) |  |  |
| A0A136HG02_9GAMM | Bacteria | Proteobacteria | Gammaproteobacteria | 1236 | 3(NWH) |  |  |
| A0A191ZF22_9GAMM | Bacteria | Proteobacteria | Gammaproteobacteria | 1236 | 3(NWH) |  |  |
| A0A0S8A6W6_9GAMM | Bacteria | Proteobacteria | Gammaproteobacteria | 1236 | 3(NWH) |  |  |
| A0A1E2V8Y3_9GAMM | Bacteria | Proteobacteria | Gammaproteobacteria | 1236 | 3(NWH) |  |  |
| A0A084IHJ3_9GAMM | Bacteria | Proteobacteria | Gammaproteobacteria | 1236 | 3(NWH) |  |  |
| U2EQX8_9GAMM | Bacteria | Proteobacteria | Gammaproteobacteria | 1236 | 3(NWH) |  |  |
| A0A1E4L4P8_9PROT | Bacteria | Proteobacteria | Proteobacteria | 1224 | 3(NWH) |  |  |
| A0A0Q5MDC5_9BURK | Bacteria | Proteobacteria | Burkholderiales | 80840 | 3(NWH) |  |  |
| A1TPJ7_ACIAC | Bacteria | Proteobacteria | Acidovorax_citrulli | 397945 | 3(NWH) |  |  |
| A0A1A8T779_9GAMM | Bacteria | Proteobacteria | Gammaproteobacteria | 1236 | 3(NWH) |  |  |
| A0A0C5V1Q7_9GAMM | Bacteria | Proteobacteria | Gammaproteobacteria | 1236 | 3(NWH) |  |  |
| A0A1B1YPY0_9GAMM | Bacteria | Proteobacteria | Gammaproteobacteria | 1236 | 3(NWH) |  |  |
| G2DZS7_9GAMM | Bacteria | Proteobacteria | Gammaproteobacteria | 1236 | 3(NWH) |  |  |
| A1KBW1_AZOSB | Bacteria | Proteobacteria | Azoarcus_sp | 62928 | 3(NWH) |  |  |
| C1DFU6_AZOVD | Bacteria | Proteobacteria | Azotobacter_vinelandii | 322710 | 3(NWH) |  |  |
| I3YF20_THIV6 | Bacteria | Proteobacteria | Thiocystis_violascens | 765911 | 3(NWH) |  |  |
| A0A0U1PZN7_9BURK | Bacteria | Proteobacteria | Burkholderiales | 80840 | 3(NWH) |  |  |
| A0A133XIQ8_9RHOO | Bacteria | Proteobacteria | Rhodocyclales | 206389 | 3(NWH) |  |  |
| Q47G09_DECAR | Bacteria | Proteobacteria | Dechloromonas_  aromatica | 159087 | 3(NWH) |  |  |
| G8QQ13_DECSP | Bacteria | Proteobacteria | Dechlorosoma_suillum | 640081 | 3(NWH) |  |  |
| A0A0F7KP90_9SPHN | Bacteria | Proteobacteria | Sphingomonadales | 204457 | 3(NWH) |  |  |
| V4TC33_9RHIZ | Bacteria | Proteobacteria | Rhizobiales | 356 | 3(NWH) |  |  |
| A8HW19_AZOC5 | Bacteria | Proteobacteria | Azorhizobium_  caulinodans | 438753 | 3(NWH) |  |  |
| A7IN18_XANP2 | Bacteria | Proteobacteria | Xanthobacter_  autotrophicus | 78245 | 3(NWH) |  |  |
| A0A0K2DKR4_9RHIZ | Bacteria | Proteobacteria | Rhizobiales | 356 | 3(NWH) |  |  |
| D7AAL6_STAND | Bacteria | Proteobacteria | Starkeya_novella | 639283 | 3(NWH) |  |  |
| W3RIQ4_9BRAD | Bacteria | Proteobacteria | Bradyrhizobiaceae | 41294 | 3(NWH) |  |  |
| A0A0Q7TDY5_9RHIZ | Bacteria | Proteobacteria | Rhizobiales | 356 | 3(NWH) |  |  |
| J6LJE2_9RHOB | Bacteria | Proteobacteria | Rhodobacterales | 204455 | 3(NWH) |  |  |
| A0A0D1NJE3_BRAEL | Bacteria | Proteobacteria | Bradyrhizobium_elkanii | 29448 | 3(NWH) |  |  |
| A0A120FLP4_9BRAD | Bacteria | Proteobacteria | Bradyrhizobiaceae | 41294 | 3(NWH) |  |  |
| F7QKR5_9BRAD | Bacteria | Proteobacteria | Bradyrhizobiaceae | 41294 | 3(NWH) |  |  |
| B6JAV4_OLICO | Bacteria | Proteobacteria | Oligotropha_  carboxidovorans | 504832 | 3(NWH) |  |  |
| Q3SQ62_NITWN | Bacteria | Proteobacteria | Nitrobacter_  winogradskyi | 323098 | 3(NWH) |  |  |
| A0A163YS83_9BRAD | Bacteria | Proteobacteria | Bradyrhizobiaceae | 41294 | 3(NWH) |  |  |
| A0A0D7ET67_RHOPL | Bacteria | Proteobacteria | Rhodopseudomonas_  palustris | 1076 | 3(NWH) |  |  |
| Q217A5_RHOPB | Bacteria | Proteobacteria | Rhodopseudomonas_  palustris | 316056 | 3(NWH) |  |  |
| A0A0D6JFD2_9RHIZ | Bacteria | Proteobacteria | Rhizobiales | 356 | 3(NWH) |  |  |
| A0A109BCB0_HYPSL | Bacteria | Proteobacteria | Hyphomicrobium_  sulfonivorans | 121290 | 3(NWH) |  |  |
| V5SF37_9RHIZ | Bacteria | Proteobacteria | Rhizobiales | 356 | 3(NWH) |  |  |
| A0A0A8K4K4_9RHIZ | Bacteria | Proteobacteria | Rhizobiales | 356 | 3(NWH) |  |  |
| A0A1E3VZ41_9RHIZ | Bacteria | Proteobacteria | Rhizobiales | 356 | 3(NWH) |  |  |
| A0A1E3WAY3_9RHIZ | Bacteria | Proteobacteria | Rhizobiales | 356 | 3(NWH) |  |  |
| A0A1E3VNC2_9RHIZ | Bacteria | Proteobacteria | Rhizobiales | 356 | 3(NWH) |  |  |
| A0A1E2S159_9RHIZ | Bacteria | Proteobacteria | Rhizobiales | 356 | 3(NWH) |  |  |
| A0A0S2ELA0_9RHIZ | Bacteria | Proteobacteria | Rhizobiales | 356 | 3(NWH) |  |  |
| A0A175RL64_9RHIZ | Bacteria | Proteobacteria | Rhizobiales | 356 | 3(NWH) |  |  |
| A0A0Q5GU57_9RHIZ | Bacteria | Proteobacteria | Rhizobiales | 356 | 3(NWH) |  |  |
| A0A0B1Q3Z9_9RHIZ | Bacteria | Proteobacteria | Rhizobiales | 356 | 3(NWH) |  |  |
| A0A0D2W186_9PROT | Bacteria | Proteobacteria | Proteobacteria | 1224 | 3(NWH) |  |  |
| W9HBV7_9PROT | Bacteria | Proteobacteria | Proteobacteria | 1224 | 3(NWH) |  |  |
| Q0G7X1_9RHIZ | Bacteria | Proteobacteria | Rhizobiales | 356 | 3(NWH) |  |  |
| B2JSL5_PARP8 | Bacteria | Proteobacteria | Paraburkholderia_  phymatum | 391038 | 3(NWH) |  |  |
| A0A0S1Y6U5_9BORD | Bacteria | Proteobacteria | Bordetella | 517 | 3(NWH) |  |  |
| A0A069PU02_9BURK | Bacteria | Proteobacteria | Burkholderiales | 80840 | 3(NWH) |  |  |
| Q13QE3_PARXL | Bacteria | Proteobacteria | Paraburkholderia_  xenovorans | 266265 | 3(NWH) |  |  |
| A0A0Q5P8X5_9BURK | Bacteria | Proteobacteria | Burkholderiales | 80840 | 3(NWH) |  |  |
| B5WNN4_9BURK | Bacteria | Proteobacteria | Burkholderiales | 80840 | 3(NWH) |  |  |
| G8MEM6_9BURK | Bacteria | Proteobacteria | Burkholderiales | 80840 | 3(NWH) |  |  |
| A0A0L0LZP1_9BURK | Bacteria | Proteobacteria | Burkholderiales | 80840 | 3(NWH) |  |  |
| A0A0K9JRN4_9BURK | Bacteria | Proteobacteria | Burkholderiales | 80840 | 3(NWH) |  |  |
| A0A0L1L1W5_9BURK | Bacteria | Proteobacteria | Burkholderiales | 80840 | 3(NWH) |  |  |
| A0A0K9JXR9_9BURK | Bacteria | Proteobacteria | Burkholderiales | 80840 | 3(NWH) |  |  |
| A0A0L0M8L7_9BURK | Bacteria | Proteobacteria | Burkholderiales | 80840 | 3(NWH) |  |  |
| G4MEL6_9BURK | Bacteria | Proteobacteria | Burkholderiales | 80840 | 3(NWH) |  |  |
| A0A0C1ZBA1_9BURK | Bacteria | Proteobacteria | Burkholderiales | 80840 | 3(NWH) |  |  |
| A0A1A5X484_9BURK | Bacteria | Proteobacteria | Burkholderiales | 80840 | 3(NWH) |  |  |
| C5ABZ0_BURGB | Bacteria | Proteobacteria | Burkholderia_glumae | 626418 | 3(NWH) |  |  |
| Q1YJB4_AURMS | Bacteria | Proteobacteria | Aurantimonas_  manganoxydans | 287752 | 3(NWH) |  |  |
| A0A0Q6D2Z4_9RHIZ | Bacteria | Proteobacteria | Rhizobiales | 356 | 3(NWH) |  |  |
| A0A0J6UA14_9RHIZ | Bacteria | Proteobacteria | Rhizobiales | 356 | 3(NWH) |  |  |
| A0A0Q4ZCQ3_9RHIZ | Bacteria | Proteobacteria | Rhizobiales | 356 | 3(NWH) |  |  |
| C5AWA2_METEA | Bacteria | Proteobacteria | Methylobacterium_  extorquens | 272630 | 3(NWH) |  |  |
| A0A0Q4XTJ4_9RHIZ | Bacteria | Proteobacteria | Rhizobiales | 356 | 3(NWH) |  |  |
| A0A0Q6A2F1_9RHIZ | Bacteria | Proteobacteria | Rhizobiales | 356 | 3(NWH) |  |  |
| B8IUS6_METNO | Bacteria | Proteobacteria | Methylobacterium_  nodulans | 460265 | 3(NWH) |  |  |
| B0UI59_METS4 | Bacteria | Proteobacteria | Methylobacterium_sp | 426117 | 3(NWH) |  |  |
| Q0BUX7_GRABC | Bacteria | Proteobacteria | Granulibacter_  bethesdensis | 391165 | 3(NWH) |  |  |
| F6IIG8_9SPHN | Bacteria | Proteobacteria | Sphingomonadales | 204457 | 3(NWH) |  |  |
| G6EA58_9SPHN | Bacteria | Proteobacteria | Sphingomonadales | 204457 | 3(NWH) |  |  |
| H0TIW0_9BRAD | Bacteria | Proteobacteria | Bradyrhizobiaceae | 41294 | 3(NWH) |  |  |
| A4Z0G0_BRASO | Bacteria | Proteobacteria | Bradyrhizobium_sp | 114615 | 3(NWH) |  |  |
| A0A0Q6ABK6_9BRAD | Bacteria | Proteobacteria | Bradyrhizobiaceae | 41294 | 3(NWH) |  |  |
| Q89DL6_BRADU | Bacteria | Proteobacteria | Bradyrhizobium_  diazoefficiens | 224911 | 3(NWH) |  |  |
| A0A0R3E4X8_9BRAD | Bacteria | Proteobacteria | Bradyrhizobiaceae | 41294 | 3(NWH) |  |  |
| U2Y363_9SPHN | Bacteria | Proteobacteria | Sphingomonadales | 204457 | 3(NWH) |  |  |
| D5RKZ4_9PROT | Bacteria | Proteobacteria | Proteobacteria | 1224 | 3(NWH) |  |  |
| H0A543_9PROT | Bacteria | Proteobacteria | Proteobacteria | 1224 | 3(NWH) |  |  |
| A0A0Q8QVI2_9SPHN | Bacteria | Proteobacteria | Sphingomonadales | 204457 | 3(NWH) |  |  |
| A0A0B1ZKY4_9SPHN | Bacteria | Proteobacteria | Sphingomonadales | 204457 | 3(NWH) |  |  |
| G8ANC8_AZOBR | Bacteria | Proteobacteria | Azospirillum_  brasilense | 192 | 3(NWH) |  |  |
| A0A0Q7CZN9_9CAUL | Bacteria | Proteobacteria | Caulobacteraceae | 76892 | 3(NWH) |  |  |
| G7ZF76_AZOL4 | Bacteria | Proteobacteria | Azospirillum_lipoferum | 862719 | 3(NWH) |  |  |
| Q1YLW7_AURMS | Bacteria | Proteobacteria | Aurantimonas_  manganoxydans | 287752 | 3(NWH) |  |  |
| A0A0B3RUT5_9RHOB | Bacteria | Proteobacteria | Rhodobacterales | 204455 | 3(NWH) |  |  |
| A3K8B1_9RHOB | Bacteria | Proteobacteria | Rhodobacterales | 204455 | 3(NWH) |  |  |
| A0A0W7WL00_9RHOB | Bacteria | Proteobacteria | Rhodobacterales | 204455 | 3(NWH) |  |  |
| A0A1B1PSZ2_9RHOB | Bacteria | Proteobacteria | Rhodobacterales | 204455 | 3(NWH) |  |  |
| S9RDS0_9RHOB | Bacteria | Proteobacteria | Rhodobacterales | 204455 | 3(NWH) |  |  |
| Q0FGS4_PELBH | Bacteria | Proteobacteria | Pelagibaca_  bermudensis | 314265 | 3(NWH) |  |  |
| W6KAE6_9PROT | Bacteria | Proteobacteria | Proteobacteria | 1224 | 3(NWH) |  |  |
| K9GMS6_9PROT | Bacteria | Proteobacteria | Proteobacteria | 1224 | 3(NWH) |  |  |
| A0A0D6P616_9PROT | Bacteria | Proteobacteria | Proteobacteria | 1224 | 3(NWH) |  |  |
| A0A0W0A5G2_9PROT | Bacteria | Proteobacteria | Proteobacteria | 1224 | 3(NWH) |  |  |
| A0A0H5BC55_BLAVI | Bacteria | Proteobacteria | Blastochloris_viridis | 1079 | 3(NWH) |  |  |
| A0A192IMR2_9RHIZ | Bacteria | Proteobacteria | Rhizobiales | 356 | 3(NWH) |  |  |
| A0A0Q6EX43_9RHIZ | Bacteria | Proteobacteria | Rhizobiales | 356 | 3(NWH) |  |  |
| A0A0Q6CN13_9RHIZ | Bacteria | Proteobacteria | Rhizobiales | 356 | 3(NWH) |  |  |
| A0A0Q6DQF3_9RHIZ | Bacteria | Proteobacteria | Rhizobiales | 356 | 3(NWH) |  |  |
| U7P0U6_9GAMM | Bacteria | Proteobacteria | Gammaproteobacteria | 1236 | 3(NWH) |  |  |
| A0A0X8HBB1_9GAMM | Bacteria | Proteobacteria | Gammaproteobacteria | 1236 | 3(NWH) |  |  |
| A0A139CBL4_9GAMM | Bacteria | Proteobacteria | Gammaproteobacteria | 1236 | 3(NWH) |  |  |
| W7QPS6_9GAMM | Bacteria | Proteobacteria | Gammaproteobacteria | 1236 | 3(NWH) |  |  |
| A0A086D693_9GAMM | Bacteria | Proteobacteria | Gammaproteobacteria | 1236 | 3(NWH) |  |  |
| A0A172YB90_9GAMM | Bacteria | Proteobacteria | Gammaproteobacteria | 1236 | 3(NWH) |  |  |
| A0A1E4C1S4_9RHIZ | Bacteria | Proteobacteria | Rhizobiales | 356 | 3(NWH) |  |  |
| A0A101W7I8_9PROT | Bacteria | Proteobacteria | Proteobacteria | 1224 | 3(NWH) |  |  |
| A0A086MGR5_9RHIZ | Bacteria | Proteobacteria | Rhizobiales | 356 | 3(NWH) |  |  |
| A0A1B2EFT8_9RHIZ | Bacteria | Proteobacteria | Rhizobiales | 356 | 3(NWH) |  |  |
| B8ER95_METSB | Bacteria | Proteobacteria | Methylocella_silvestris | 395965 | 3(NWH) |  |  |
| A0A0P6VNS7_9RHIZ | Bacteria | Proteobacteria | Rhizobiales | 356 | 3(NWH) |  |  |
| A0A0Q9JH66_9BRAD | Bacteria | Proteobacteria | Bradyrhizobiaceae | 41294 | 3(NWH) |  |  |
| A0A085EW32_9BRAD | Bacteria | Proteobacteria | Bradyrhizobiaceae | 41294 | 3(NWH) |  |  |
| A0A126NYM5_9BRAD | Bacteria | Proteobacteria | Bradyrhizobiaceae | 41294 | 3(NWH) |  |  |
| A0A0Q3KJD4_9BRAD | Bacteria | Proteobacteria | Bradyrhizobiaceae | 41294 | 3(NWH) |  |  |
| A0A117MSU7_9BRAD | Bacteria | Proteobacteria | Bradyrhizobiaceae | 41294 | 3(NWH) |  |  |
| A0A1B3NGM2_9BRAD | Bacteria | Proteobacteria | Bradyrhizobiaceae | 41294 | 3(NWH) |  |  |
| A0A0Q9HCN8_9BRAD | Bacteria | Proteobacteria | Bradyrhizobiaceae | 41294 | 3(NWH) |  |  |
| A0A0N1B5K0_9BRAD | Bacteria | Proteobacteria | Bradyrhizobiaceae | 41294 | 3(NWH) |  |  |
| A0A0Q6KBM8_9BRAD | Bacteria | Proteobacteria | Bradyrhizobiaceae | 41294 | 3(NWH) |  |  |
| A0A177PK25_9RHIZ | Bacteria | Proteobacteria | Rhizobiales | 356 | 3(NWH) |  |  |
| A0A1A6FPJ2_9RHIZ | Bacteria | Proteobacteria | Rhizobiales | 356 | 3(NWH) |  |  |
| J7QVB4_METSZ | Bacteria | Proteobacteria | Methylocystis_sp | 187303 | 3(NWH) |  |  |
| B2IJU2_BEII9 | Bacteria | Proteobacteria | Beijerinckia_  indica subsp | 395963 | 3(NWH) |  |  |
| B8ENU3_METSB | Bacteria | Proteobacteria | Methylocella_silvestris | 395965 | 3(NWH) |  |  |
| K0C859_CYCSP | Bacteria | Proteobacteria | Cycloclasticus_sp | 385025 | 3(NWH) |  |  |
| E3I0H5_RHOVT | Bacteria | Proteobacteria | Rhodomicrobium_  vannielii | 648757 | 3(NWH) |  |  |
| W8SR89_9RHOB | Bacteria | Proteobacteria | Rhodobacterales | 204455 | 3(NWH) |  |  |
| A8LKX8_DINSH | Bacteria | Proteobacteria | Dinoroseobacter_  shibae | 398580 | 3(NWH) |  |  |
| A0A0D6AZS6_RHOSU | Bacteria | Proteobacteria | Rhodovulum_  sulfidophilum | 35806 | 3(NWH) |  |  |
| A0A0N8KDG0_9RHOB | Bacteria | Proteobacteria | Rhodobacterales | 204455 | 3(NWH) |  |  |
| Q3IZM9_RHOS4 | Bacteria | Proteobacteria | Rhodobacter_  sphaeroides | 272943 | 3(NWH) |  |  |
| A0A161GJB1_9RHOB | Bacteria | Proteobacteria | Rhodobacterales | 204455 | 3(NWH) |  |  |
| A0A086YAB1_9RHOB | Bacteria | Proteobacteria | Rhodobacterales | 204455 | 3(NWH) |  |  |
| A0A095WWI7_9GAMM | Bacteria | Proteobacteria | Gammaproteobacteria | 1236 | 3(NWH) |  |  |
| W6W8W8_9RHIZ | Bacteria | Proteobacteria | Rhizobiales | 356 | 3(NWH) |  |  |
| A0A095VFI2_9RHIZ | Bacteria | Proteobacteria | Rhizobiales | 356 | 3(NWH) |  |  |
| A0A081MJY8_9RHIZ | Bacteria | Proteobacteria | Rhizobiales | 356 | 3(NWH) |  |  |
| L0NHG4_9RHIZ | Bacteria | Proteobacteria | Rhizobiales | 356 | 3(NWH) |  |  |
| A0A0M3BFP6_9RHIZ | Bacteria | Proteobacteria | Rhizobiales | 356 | 3(NWH) |  |  |
| A0A0Q5CUX7_9RHIZ | Bacteria | Proteobacteria | Rhizobiales | 356 | 3(NWH) |  |  |
| A0A0Q8BJK4_9RHIZ | Bacteria | Proteobacteria | Rhizobiales | 356 | 3(NWH) |  |  |
| A0A081CQF9_9RHIZ | Bacteria | Proteobacteria | Rhizobiales | 356 | 3(NWH) |  |  |
| A9CHT4_AGRFC | Bacteria | Proteobacteria | Agrobacterium_fabrum | 176299 | 3(NWH) |  |  |
| B9JXJ0_AGRVS | Bacteria | Proteobacteria | Agrobacterium_vitis | 311402 | 3(NWH) |  |  |
| K2QW79_9RHIZ | Bacteria | Proteobacteria | Rhizobiales | 356 | 3(NWH) |  |  |
| A0A0Q7XI56_9RHIZ | Bacteria | Proteobacteria | Rhizobiales | 356 | 3(NWH) |  |  |
| A0A1E3H442_9RHIZ | Bacteria | Proteobacteria | Rhizobiales | 356 | 3(NWH) |  |  |
| G8PKB1_PSEUV | Bacteria | Proteobacteria | Pseudovibrio_sp | 911045 | 3(NWH) |  |  |
| W6REQ1_9RHIZ | Bacteria | Proteobacteria | Rhizobiales | 356 | 3(NWH) |  |  |
| K0PXY0_9RHIZ | Bacteria | Proteobacteria | Rhizobiales | 356 | 3(NWH) |  |  |
| A0A0Q6WB77_9RHIZ | Bacteria | Proteobacteria | Rhizobiales | 356 | 3(NWH) |  |  |
| K0VJ16_9RHIZ | Bacteria | Proteobacteria | Rhizobiales | 356 | 3(NWH) |  |  |
| Q2K560_RHIEC | Bacteria | Proteobacteria | Rhizobium_etli | 347834 | 3(NWH) |  |  |
| F2AEI7_RHIET | Bacteria | Proteobacteria | Rhizobium_etli | 29449 | 3(NWH) |  |  |
| A0A0B4X618_9RHIZ | Bacteria | Proteobacteria | Rhizobiales | 356 | 3(NWH) |  |  |
| J2B2J0_9RHIZ | Bacteria | Proteobacteria | Rhizobiales | 356 | 3(NWH) |  |  |
| A0A0Q8NKF1_9RHIZ | Bacteria | Proteobacteria | Rhizobiales | 356 | 3(NWH) |  |  |
| B9J859_AGRRK | Bacteria | Proteobacteria | Agrobacterium_  radiobacter | 311403 | 3(NWH) |  |  |
| N6V9E9_9RHIZ | Bacteria | Proteobacteria | Rhizobiales | 356 | 3(NWH) |  |  |
| A0A1B9RQ47_9RHIZ | Bacteria | Proteobacteria | Rhizobiales | 356 | 3(NWH) |  |  |
| U4VB73_9RHIZ | Bacteria | Proteobacteria | Rhizobiales | 356 | 3(NWH) |  |  |
| A6X526_OCHA4 | Bacteria | Proteobacteria | Ochrobactrum_  anthropi | 439375 | 3(NWH) |  |  |
| Q2YKI2_BRUA2 | Bacteria | Proteobacteria | Brucella_abortus | 359391 | 3(NWH) |  |  |
| A0A1A9FNH1_9RHIZ | Bacteria | Proteobacteria | Rhizobiales | 356 | 3(NWH) |  |  |
| X6FLI6_9RHIZ | Bacteria | Proteobacteria | Rhizobiales | 356 | 3(NWH) |  |  |
| G6YCW7_9RHIZ | Bacteria | Proteobacteria | Rhizobiales | 356 | 3(NWH) |  |  |
| M5F6F4_9RHIZ | Bacteria | Proteobacteria | Rhizobiales | 356 | 3(NWH) |  |  |
| A0A090FS72_9RHIZ | Bacteria | Proteobacteria | Rhizobiales | 356 | 3(NWH) |  |  |
| A0A117N4F1_RHILI | Bacteria | Proteobacteria | Rhizobium_loti | 381 | 3(NWH) |  |  |
| E8TJB6_MESCW | Bacteria | Proteobacteria | Mesorhizobium_  ciceri biovar biserrulae | 765698 | 3(NWH) |  |  |
| A0A1C2DND7_9RHIZ | Bacteria | Proteobacteria | Rhizobiales | 356 | 3(NWH) |  |  |
| A0A0Q6MK95_9RHIZ | Bacteria | Proteobacteria | Rhizobiales | 356 | 3(NWH) |  |  |
| Q98L66_RHILO | Bacteria | Proteobacteria | Rhizobium_loti | 266835 | 3(NWH) |  |  |
| A0A0Q7WGT6_9RHIZ | Bacteria | Proteobacteria | Rhizobiales | 356 | 3(NWH) |  |  |
| H0HST2_9RHIZ | Bacteria | Proteobacteria | Rhizobiales | 356 | 3(NWH) |  |  |
| A0A0H1ADK4_9RHIZ | Bacteria | Proteobacteria | Rhizobiales | 356 | 3(NWH) |  |  |
| A0A011VN94_9RHIZ | Bacteria | Proteobacteria | Rhizobiales | 356 | 3(NWH) |  |  |
| A0A135HW59_9RHIZ | Bacteria | Proteobacteria | Rhizobiales | 356 | 3(NWH) |  |  |
| Q11IE6_CHESB | Bacteria | Proteobacteria | Chelativorans_sp | 266779 | 3(NWH) |  |  |
| A0A0Q8BA79_9RHIZ | Bacteria | Proteobacteria | Rhizobiales | 356 | 3(NWH) |  |  |
| B9QYS4_LABAD | Bacteria | Proteobacteria | Labrenzia_alexandrii | 244592 | 3(NWH) |  |  |
| A0A0U3ET72_9RHOB | Bacteria | Proteobacteria | Rhodobacterales | 204455 | 3(NWH) |  |  |
| A0A1E3VDN3_9RHIZ | Bacteria | Proteobacteria | Rhizobiales | 356 | 3(NWH) |  |  |
| C3MGD7_SINFN | Bacteria | Proteobacteria | Sinorhizobium_fredii | 394 | 3(NWH) |  |  |
| A0A0Q6FMS2_9RHIZ | Bacteria | Proteobacteria | Rhizobiales | 356 | 3(NWH) |  |  |
| A0A1C7NU50_9RHIZ | Bacteria | Proteobacteria | Rhizobiales | 356 | 3(NWH) |  |  |
| A0A0Q7NZ70_9RHIZ | Bacteria | Proteobacteria | Rhizobiales | 356 | 3(NWH) |  |  |
| A0A0Q5WR82_9RHIZ | Bacteria | Proteobacteria | Rhizobiales | 356 | 3(NWH) |  |  |
| A0A0Q6SMM3_9RHIZ | Bacteria | Proteobacteria | Rhizobiales | 356 | 3(NWH) |  |  |
| A0A1B3M7W3_9RHIZ | Bacteria | Proteobacteria | Rhizobiales | 356 | 3(NWH) |  |  |
| A0A0D5LPH0_9RHIZ | Bacteria | Proteobacteria | Rhizobiales | 356 | 3(NWH) |  |  |
| A0A127CFR9_9RHIZ | Bacteria | Proteobacteria | Rhizobiales | 356 | 3(NWH) |  |  |
| A0A0F2PMI4_9RHIZ | Bacteria | Proteobacteria | Rhizobiales | 356 | 3(NWH) |  |  |
| A0A0N0VMF1_9RHOB | Bacteria | Proteobacteria | Rhodobacterales | 204455 | 3(NWH) |  |  |
| A0A0M7A3B9_9RHOB | Bacteria | Proteobacteria | Rhodobacterales | 204455 | 3(NWH) |  |  |
| A0A0F2RGF8_9PROT | Bacteria | Proteobacteria | Proteobacteria | 1224 | 3(NWH) |  |  |
| A0A0P1H409_9RHOB | Bacteria | Proteobacteria | Rhodobacterales | 204455 | 3(NWH) |  |  |
| A4EWR3_9RHOB | Bacteria | Proteobacteria | Rhodobacterales | 204455 | 3(NWH) |  |  |
| A3X3D9_9RHOB | Bacteria | Proteobacteria | Rhodobacterales | 204455 | 3(NWH) |  |  |
| A0A0N7LS90_9RHOB | Bacteria | Proteobacteria | Rhodobacterales | 204455 | 3(NWH) |  |  |
| A0A0P7YAL4_9RHOB | Bacteria | Proteobacteria | Rhodobacterales | 204455 | 3(NWH) |  |  |
| A0A058ZLB0_9RHOB | Bacteria | Proteobacteria | Rhodobacterales | 204455 | 3(NWH) |  |  |
| A0A176FFY8_9RHOB | Bacteria | Proteobacteria | Rhodobacterales | 204455 | 3(NWH) |  |  |
| A0A0T5P6I3_9RHOB | Bacteria | Proteobacteria | Rhodobacterales | 204455 | 3(NWH) |  |  |
| B9NQB2_9RHOB | Bacteria | Proteobacteria | Rhodobacterales | 204455 | 3(NWH) |  |  |
| A0A0L6CVN9_9RHOB | Bacteria | Proteobacteria | Rhodobacterales | 204455 | 3(NWH) |  |  |
| A0A0P1GNZ1_9RHOB | Bacteria | Proteobacteria | Rhodobacterales | 204455 | 3(NWH) |  |  |
| A0A0B5DZA8_9RHOB | Bacteria | Proteobacteria | Rhodobacterales | 204455 | 3(NWH) |  |  |
| A0A0S6WWY2_9SPHN | Bacteria | Proteobacteria | Sphingomonadales | 204457 | 3(NWH) |  |  |
| A0A1E4BUG8_9RHIZ | Bacteria | Proteobacteria | Rhizobiales | 356 | 3(NWH) |  |  |
| W4HQL7_9RHOB | Bacteria | Proteobacteria | Rhodobacterales | 204455 | 3(NWH) |  |  |
| Q0FJ41_PELBH | Bacteria | Proteobacteria | Pelagibaca_  bermudensis | 314265 | 3(NWH) |  |  |
| D0D788_9RHOB | Bacteria | Proteobacteria | Rhodobacterales | 204455 | 3(NWH) |  |  |
| X6KW59_9RHOB | Bacteria | Proteobacteria | Rhodobacterales | 204455 | 3(NWH) |  |  |
| A0A0T5NU08_9RHOB | Bacteria | Proteobacteria | Rhodobacterales | 204455 | 3(NWH) |  |  |
| A9DQF6_9RHOB | Bacteria | Proteobacteria | Rhodobacterales | 204455 | 3(NWH) |  |  |
| X7F5U0_9RHOB | Bacteria | Proteobacteria | Rhodobacterales | 204455 | 3(NWH) |  |  |
| A0A073IHA6_9RHOB | Bacteria | Proteobacteria | Rhodobacterales | 204455 | 3(NWH) |  |  |
| A0A196NZN4_9RHOB | Bacteria | Proteobacteria | Rhodobacterales | 204455 | 3(NWH) |  |  |
| Q160G3_ROSDO | Bacteria | Proteobacteria | Roseobacter_  denitrificans | 375451 | 3(NWH) |  |  |
| A0A0B4BF96_9RHOB | Bacteria | Proteobacteria | Rhodobacterales | 204455 | 3(NWH) |  |  |
| X7EMM6_9RHOB | Bacteria | Proteobacteria | Rhodobacterales | 204455 | 3(NWH) |  |  |
| Q28QF8_JANSC | Bacteria | Proteobacteria | Jannaschia_sp | 290400 | 3(NWH) |  |  |
| W5Y4H9_KOMXY | Bacteria | Proteobacteria | Komagataeibacter_  xylinus | 28448 | 3(NWH) |  |  |
| F3S623_9PROT | Bacteria | Proteobacteria | Proteobacteria | 1224 | 3(NWH) |  |  |
| Q5FP51_GLUOX | Bacteria | Proteobacteria | Gluconobacter_  oxydans | 290633 | 3(NWH) |  |  |
| K7TDX8_GLUOY | Bacteria | Proteobacteria | Gluconobacter_  oxydans | 442 | 3(NWH) |  |  |
| G6XH39_9PROT | Bacteria | Proteobacteria | Proteobacteria | 1224 | 3(NWH) |  |  |
| F7VEH9_9PROT | Bacteria | Proteobacteria | Proteobacteria | 1224 | 3(NWH) |  |  |
| A0A023D3E4_ACIMT | Bacteria | Proteobacteria | Acidomonas_  methanolica | 437 | 3(NWH) |  |  |
| W5YDT4_KOMXY | Bacteria | Proteobacteria | Komagataeibacter_  xylinus | 28448 | 3(NWH) |  |  |
| D5QHZ6_KOMHA | Bacteria | Proteobacteria | Komagataeibacter_  hansenii | 436 | 3(NWH) |  |  |
| A0A060QFM4_9PROT | Bacteria | Proteobacteria | Proteobacteria | 1224 | 3(NWH) |  |  |
| F9U5X0_9GAMM | Bacteria | Proteobacteria | Gammaproteobacteria | 1236 | 3(NWH) |  |  |
| H6SMU7_RHOPH | Bacteria | Proteobacteria | Rhodospirillum_  photometricum | 1084 | 3(NWH) |  |  |
| W8KL48_HALHR | Bacteria | Proteobacteria | Halorhodospira_  halochloris | 1052 | 3(NWH) |  |  |
| J1JZQ3_9RHIZ | Bacteria | Proteobacteria | Rhizobiales | 356 | 3(NWH) |  |  |
| A0A0S8A0C2_9GAMM | Bacteria | Proteobacteria | Gammaproteobacteria | 1236 | 3(NWH) |  |  |
| A0A0S2JEB6_9GAMM | Bacteria | Proteobacteria | Gammaproteobacteria | 1236 | 3(NWH) |  |  |
| D4ZKN8_SHEVD | Bacteria | Proteobacteria | Shewanella_violacea | 637905 | 3(NWH) |  |  |
| A0A191ZG87_9GAMM | Bacteria | Proteobacteria | Gammaproteobacteria | 1236 | 3(NWH) |  |  |
| D0L0J1_HALNC | Bacteria | Proteobacteria | Halothiobacillus_  neapolitanus | 555778 | 3(NWH) |  |  |
| H8YVF6_9GAMM | Bacteria | Proteobacteria | Gammaproteobacteria | 1236 | 3(NWH) |  |  |
| A0A1E2UYF3_9GAMM | Bacteria | Proteobacteria | Gammaproteobacteria | 1236 | 3(NWH) |  |  |
| F9ZRI1_ACICS | Bacteria | Proteobacteria | Acidithiobacillus_  caldus | 990288 | 3(NWH) |  |  |
| A0A179BJY2_ACIFR | Bacteria | Proteobacteria | Acidithiobacillus_  ferrooxidans | 920 | 3(NWH) |  |  |
| A0A060ULT0_9PROT | Bacteria | Proteobacteria | Proteobacteria | 1224 | 3(NWH) |  |  |
| B7J7X6_ACIF2 | Bacteria | Proteobacteria | Acidithiobacillus_  ferrooxidans | 243159 | 3(NWH) |  |  |
| A0A1C2I1R0_ACITH | Bacteria | Proteobacteria | Acidithiobacillus_  thiooxidans | 930 | 3(NWH) |  |  |
| A0A1A6C5M2_9GAMM | Bacteria | Proteobacteria | Gammaproteobacteria | 1236 | 3(NWH) |  |  |
| A7BZR6_9GAMM | Bacteria | Proteobacteria | Gammaproteobacteria | 1236 | 3(NWH) |  |  |
| Q0A9W5_ALKEH | Bacteria | Proteobacteria | Alkalilimnicola_ehrlichii | 187272 | 3(NWH) |  |  |
| S2L192_9GAMM | Bacteria | Proteobacteria | Gammaproteobacteria | 1236 | 3(NWH) |  |  |
| E1V6S4_HALED | Bacteria | Proteobacteria | Halomonas_elongata | 768066 | 3(NWH) |  |  |
| A0A098RKA2_9GAMM | Bacteria | Proteobacteria | Gammaproteobacteria | 1236 | 3(NWH) |  |  |
| A0A0F4RC52_9GAMM | Bacteria | Proteobacteria | Gammaproteobacteria | 1236 | 3(NWH) |  |  |
| A0A0X8HC85_9GAMM | Bacteria | Proteobacteria | Gammaproteobacteria | 1236 | 3(NWH) |  |  |
| Q1QUM3_CHRSD | Bacteria | Proteobacteria | Chromohalobacter_  salexigens | 290398 | 3(NWH) |  |  |
| A0A0P9C414_9GAMM | Bacteria | Proteobacteria | Gammaproteobacteria | 1236 | 3(NWH) |  |  |
| A0A017STS4_9DELT | Bacteria | Proteobacteria | Deltaproteobacteria | 28221 | 3(NWH) |  |  |
| S4Y799_SORCE | Bacteria | Proteobacteria | Sorangium_cellulosum | 56 | 3(NWH) |  |  |
| A9GTF2_SORC5 | Bacteria | Proteobacteria | Sorangium_cellulosum | 448385 | 3(NWH) |  |  |
| A0A0S8BYZ4_9DELT | Bacteria | Proteobacteria | Deltaproteobacteria | 28221 | 3(NWH) |  |  |
| A6GFD3_9DELT | Bacteria | Proteobacteria | Deltaproteobacteria | 28221 | 3(NWH) |  |  |
| A0LA75_MAGMM | Bacteria | Proteobacteria | Magnetococcus_  marinus | 156889 | 3(NWH) |  |  |
| X2HBR6_9GAMM | Bacteria | Proteobacteria | Gammaproteobacteria | 1236 | na |  | failed iqtree; removed from phylogenetic analysis |
| A7I3R4_CAMHC | Bacteria | Proteobacteria | Campylobacter_  hominis | 360107 | na |  | failed iqtree; removed from phylogenetic analysis |
| A0A085WJ43_9DELT | Bacteria | Proteobacteria | Deltaproteobacteria | 28221 | na |  | failed iqtree; removed from phylogenetic analysis |
| G8AFH9_AZOBR | Bacteria | Proteobacteria | Azospirillum_  brasilense | 192 | na |  | failed iqtree; removed from phylogenetic analysis |
| A0A0Q9EX93_9GAMM | Bacteria | Proteobacteria | Gammaproteobacteria | 1236 | na |  | failed iqtree; removed from phylogenetic analysis |
| A0A176I893_9GAMM | Bacteria | Proteobacteria | Gammaproteobacteria | 1236 | na |  | failed iqtree; removed from phylogenetic analysis |
| A0A080KBG6_9GAMM | Bacteria | Proteobacteria | Gammaproteobacteria | 1236 | na |  | failed iqtree; removed from phylogenetic analysis |
| A0A080K8U9_9GAMM | Bacteria | Proteobacteria | Gammaproteobacteria | 1236 | na |  | failed iqtree; removed from phylogenetic analysis |
| E8UEJ4_TAYEM | Bacteria | Proteobacteria | Taylorella_  equigenitalis | 937774 | na |  | failed iqtree; removed from phylogenetic analysis |
| R7I975_9BURK | Bacteria | Proteobacteria | Burkholderiales | 80840 | na |  | failed iqtree; removed from phylogenetic analysis |
| A0A0U2QRE8_9ALTE | Bacteria | Proteobacteria | Alteromonadaceae | 72275 | na |  | long branch; removed from final phylogenetic analysis |
| A0A091AC91_9DELT | Bacteria | Proteobacteria | Deltaproteobacteria | 28221 | na |  | long branch; removed from final phylogenetic analysis |
| Q39WX1_GEOMG | Bacteria | Proteobacteria | Geobacter_  metallireducens | 269799 | na |  | long branch; removed from final phylogenetic analysis |
| Q82PL7_STRAW | Bacteria | Tenericutes | Phytoplasma_sp | 59889 | 3(NWH) |  |  |
| U4KRG7_ACHPJ | Bacteria | Tenericutes | Acholeplasma_palmae | 1318466 | na |  | long branch; removed from final phylogenetic analysis |
| A0A061A9P6_9MOLU | Bacteria | Tenericutes | Mollicutes | 31969 | na |  | long branch; removed from final phylogenetic analysis |
| A9NGT9_ACHLI | Bacteria | Tenericutes | Acholeplasma_laidlawii | 441768 | na |  | long branch; removed from final phylogenetic analysis |
| U4KPB2_9MOLU | Bacteria | Tenericutes | Mollicutes | 31969 | na |  | long branch; removed from final phylogenetic analysis |
